# TurboID-based proximity labeling reveals that UBR7 is a regulator of N NLR immune receptor-mediated immunity

**DOI:** 10.1038/s41467-019-11202-z

**Published:** 2019-07-19

**Authors:** Yongliang Zhang, Gaoyuan Song, Neeraj K. Lal, Ugrappa Nagalakshmi, Yuanyuan Li, Wenjie Zheng, Pin-jui Huang, Tess C. Branon, Alice Y. Ting, Justin W. Walley, Savithramma P. Dinesh-Kumar

**Affiliations:** 1 Department of Plant Biology and The Genome Center, College of Biological Sciences, University of California, Davis, Davis, CA 95616 USA; 2 State Key Laboratory of Agro-Biotechnology and Ministry of Agriculture Key Laboratory of Soil Microbiology, College of Biological Sciences, China Agricultural University, Beijing, 100193 China; 3 Department of Plant Pathology and Microbiology, Iowa State University, Ames, IA 50011 USA; 4 Departments of Genetics, Stanford University, Stanford, CA 94305 USA; 5 Department of Chemistry, Stanford University, Stanford, CA 94305 USA; 6 Department of Biology, Stanford University, Stanford, CA 94305 USA; 7 Department of Chemistry, Massachusetts Institute of Technology, Cambridge, MA 02139 USA; 8 Chan Zuckerberg Biohub, San Francisco, CA 94158 USA

**Keywords:** Plant immunity, Proteomic analysis

## Abstract

Nucleotide-binding leucine-rich repeat (NLR) immune receptors play a critical role in defence against pathogens in plants and animals. However, we know very little about NLR-interacting proteins and the mechanisms that regulate NLR levels. Here, we used proximity labeling (PL) to identify the proteome proximal to N, which is an NLR that confers resistance to *Tobacco mosaic virus* (TMV). Evaluation of different PL methods indicated that TurboID-based PL provides more efficient levels of biotinylation than BioID and BioID2 in plants. TurboID-based PL of N followed by quantitative proteomic analysis and genetic screening revealed multiple regulators of *N*-mediated immunity. Interestingly, a putative E3 ubiquitin ligase, UBR7, directly interacts with the TIR domain of N. UBR7 downregulation leads to an increased amount of N protein and enhanced TMV resistance. TMV-p50 effector disrupts the N-UBR7 interaction and relieves negative regulation of N. These findings demonstrate the utility of TurboID-based PL in plants and the N-interacting proteins we identified enhance our understanding of the mechanisms underlying NLR regulation.

## Abstract (web summary)

Plant NLR receptors trigger immune signaling following recognition of pathogen effectors. Here, Zhang *et al*. optimize a TurboID-based proximity labeling approach and show that it can be used to identify interacting partners of N, an NLR that confers resistance to *Tobacco mosaic virus*.

## Introduction

Plants have a sophisticated immune system to defend against pathogen attack. RNA silencing is viewed as a primary antiviral defense system of plants^1^. Most plant viruses have RNA genomes and form double-stranded viral RNA (dsRNA) structures or dsRNA replicative intermediates, which can trigger RNA silencing‐based antiviral immunity. To counteract RNA silencing-based defense, plant viruses have evolved viral suppressors of RNA silencing (VSRs) which target different steps in the silencing pathway^2^, therefore, preventing the first layer of defense responses. Just as the ability of VSRs to suppress RNA silencing, non-viral pathogens such as bacteria, fungi, and oomycetes secrete effectors to inhibit the basal defense layer, called pathogen-associated molecular pattern (PAMP)-triggered immunity (PTI)^3^. However, during the co-evolutionary arms race between plant and pathogens, plants evolved a second layer of defense responses called effector-triggered immunity (ETI), which recognizes pathogen effectors and restricts pathogens to the infection site^4^. ETI is mostly mediated by intracellular nucleotide-binding leucine-rich repeat (NLR) class of immune receptors^4,5^. The NLRs in addition to recognizing non-viral pathogen effectors also recognize either VSRs or other viral-encoded proteins^6^.

A typical plant NLR either contains a Toll-interleukin-1-receptor (TIR) homology domain or a coiled-coil (CC) domain at the amino terminus^4,5^. Unlike mammalian NLRs that recognize PAMPs, plant NLRs recognize pathogen effectors/VSRs/viral proteins either directly or indirectly. This recognition activates immune signaling, which often leads to programmed cell death (PCD) at the site of pathogen infection and containment of pathogen to the infection site^4,5^. Although many plant NLRs that recognize different pathogen effectors have been identified, our knowledge of proteins that directly associate with NLRs to regulate immune signaling remains elusive. In particular, we have a limited understanding as to how NLR expression is regulated in order to avoid autoimmunity and detrimental effects on plant fitness^7^.

The N TIR-NLR is a well-studied viral immune receptor that confers resistance to *Tobacco mosaic virus* (TMV)^8^ and is a good model for understanding TIR-NLR-based immunity. The N NLR recognizes a 50 kDa helicase domain within the TMV replicase (p50), thereby triggering a hypersensitive response-type of programmed cell death (HR-PCD) that functions to restrict TMV to the infection site^9^. Recognition of TMV p50 requires chloroplast-localized N receptor interacting protein 1 (NRIP1)^10^. Although viral effector recognition occurs in the cytoplasm^10,11^, the N immune receptor interaction with the SQUAMOSA PROMOTER BINDING PROTEIN-LIKE 6 (SPL6) transcription factor post-TMV recognition is required to activate the immune response^12^. Besides these N interacting proteins and Hsp90 molecular chaperone^13^, components that constitute the N NLR immune receptor complex and other NLR complexes remain largely unknown. N is generally expressed at very low level and N protein accumulation increases in response to TMV infection or with expression of TMV p50 effector^12,14^. However, mechanistic basis of stabilization of N protein post-TMV infection or post-p50 expression is currently unknown.

Methods such as affinity purification coupled with mass spectrometry (AP-MS) have so far identified very few plant NLR interacting partners. This is because AP-MS fails to identify proteins that interact weakly or transiently with a target protein. Recently, enzyme-catalyzed proximity labeling (PL) approaches have been developed to overcome some of these drawbacks^15–17^. In PL approaches, an enzyme that can catalyze the biotinylation of endogenous proteins in a proximity-dependent manner is fused to a target protein of interest, which allows proximal and interacting proteins to be tagged in the presence of biotin. The biotinylated interacting proteins are captured by streptavidin-based affinity purification followed by identification of these proteins by MS^15,18–20^. Currently, prevalent PL methods are based on engineered ascorbate peroxidase (APEX)^18,19^ and a mutant *Escherichia coli* biotin ligase BirA^R118G^ (BioID)^15^. In contrast to AP-MS, which involves biochemical isolation of intact protein complexes, in PL, the labeling of proteins is executed in living cells in the native cellular environment. Thus, PL enables identification of weakly and transiently interacting proteins, which are typically lost during affinity purification experiments. Although APEX offers rapid tagging kinetics^18,19^, the utilization of toxic H_2_O_2_ during labeling, as well as high endogenous plant peroxidase activity, make it unsuitable for plants. The use of toxic H_2_O_2_ is bypassed in the BioID-based labeling system, but longer incubation time with biotin (16–24 h) and higher incubation temperature (37 °C) are required for efficient labeling^15^. Although a smaller biotin ligase, BioID2, from *Aquifex aeolicus* has been described, conditions required for biotinylation of interacting proteins are similar to those of BioID^21^. The conditions required for BioID and BioID2 are not optimal for in vivo PL in plants, which has resulted in limited deployment of BioID-based PL in plants^22–24^. Recently, Branon et al. evolved the *E. coli* biotin ligase BirA using yeast display and generated new promiscuous labeling variants TurboID and miniTurboID which allow rapid nontoxic proximity labeling in just 10 min^20^. While the utility of TurboID has been demonstrated in various animal models, there is currently no report of TurboID-based proximity labeling in plants.

In this study, we compared the activity and efficiency of different biotin ligases for proximity labeling in plants. Our results revealed that TurboID outperforms other biotin ligases in tagging biotin to the nearby proteins in plant cells at room temperature. We optimized the TurboID approach in the *Nicotiana benthamiana* model plant system and used it to identify interacting proteins of N, a TIR-NLR immune receptor. TurboID-based PL combined with isobaric tagging and mass spectrometry (MS) identified a number of interesting proteins that associate with N. Genetic screening and bimolecular fluorescence complementation (BiFC) analysis established that some of these interacting proteins are involved in *N*-mediated resistance. Finally, we describe characterization of one of the candidate interactors, UBR7, which is a putative E3 ubiquitin ligase. We demonstrate that UBR7 negatively regulates N protein level and *N*-mediated resistance to TMV.

## Results

### TurboID outperforms BioID and BioID2 for PL in plants

*N. benthamiana* is a widely used model for many aspects of plant biology, including host-pathogen interactions^25,26^. Thus, we optimized procedures for in vivo promiscuous protein biotinylation and enrichment of the biotinylated proteins in *N. benthamiana*, thereby enabling identification of the enriched interacting proteins by liquid chromatography–tandem mass spectrometry (LC–MS/MS).

**Fig. 1:**
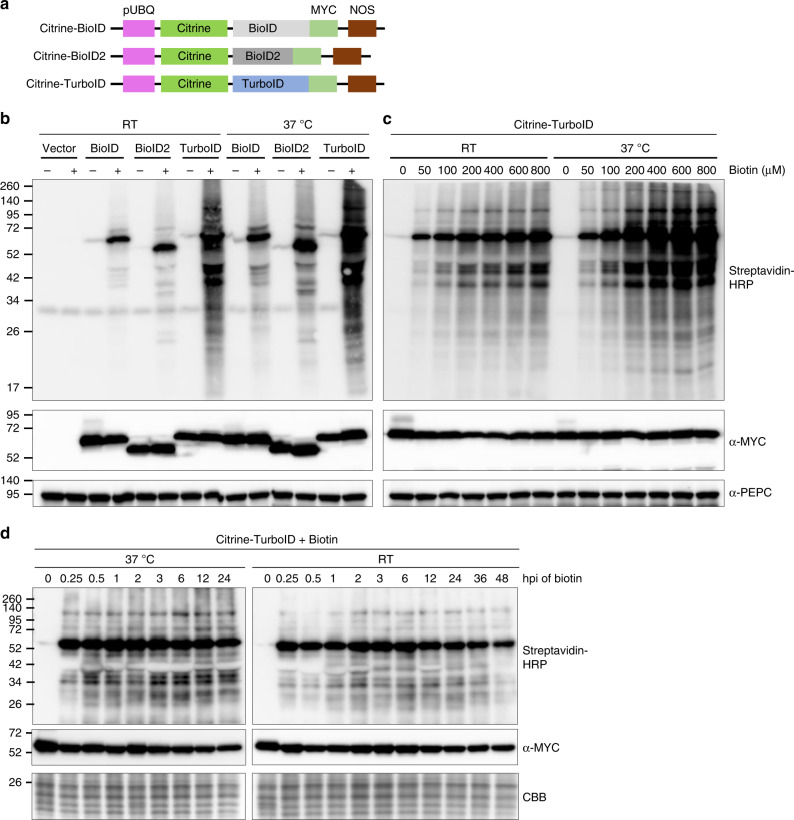
Comparison and characterization of promiscuous protein biotinylation by different biotin ligases in plant. **a** Diagram of the expression cassettes used for the expression of three biotin ligases. Citrine was fused to the N-terminus while a MYC tag was added to the C-terminus of BioID, BioID2 and TurboID. Expression was under the control of *Arabidopsis* ubiquitin-10 promoter (pUBQ) and nopaline synthase terminator (NOSt). **b** The biotin ligases promiscuously biotinylate endogenous proteins in plant cells with varying efficiencies. *N. benthamiana* leaves were agroinfiltrated with the agrobacterium containing citrine-BioID-3xMYC (BioID), citrine-BioID2-3xMYC (BioID2) and citrine-TurboID-3xMYC (TurboID), or the empty vector control (vector), respectively. 36 h post-agroinfiltration (hpi), medium containing the buffer (−) or 200 µM biotin (+) were infiltrated into the previously agroinfiltrated leaves. Infiltrated *N. benthamiana* plants were directly incubated at room temperature (RT) or in a 37 °C chamber. Western blot analysis was performed on tissue collected 12 h after infiltration of biotin. Streptavidin-HRP and anti-MYC antibodies were used for detection of biotinylated proteins (top panel) and different biotin ligases (middle panel), respectively. PEPC served as loading control for equal protein loading (bottom panel). The molecular weight size markers in kDa are indicated at the left of each panel. An additional repeat of this experiment is shown in Supplementary Fig. 1b. **c** Determination of the optimal biotin concentrations required for TurboID-based proximity labeling *in planta*. Different concentrations of biotin, as indicated above the panels, were infiltrated into the *N. benthamiana* leaves expressing the citrine-TurboID. These plants were then incubated in the 37 °C chamber or at room temperature (RT) for 8 h followed by western blot analysis as described in (**b**). PEPC served as loading control for equal protein loading (bottom panel). The molecular weight size markers in kDa are indicated at the left of each panel. Additional repeat of this experiment is shown in Supplementary Fig. 1c. **d** Effect of incubation time on TurboID-based proximity labeling in plants. *N. benthamiana* leaves expressing citrine-TurboID were infiltrated with 200 µM biotin, plants were then incubated under 37 °C or RT followed by collection of leaves at different time points as indicated above the panels. Western blot analysis was then carried out as described in Fig. 1b. Due to the instability of rbcL protein levels at different time points after biotin treatment (Supplementary Fig. 2), the protein bands below that of the rbcL in the Coomassie Brilliant Blue (CBB)-stained gel is shown as a loading control (bottom panel). The molecular weight size markers in kDa are indicated at the left of each panel. Source data are provided as a Source Data file

To characterize and compare the activity of different biotin ligases in plants, we fused citrine fluorescent protein and a MYC tag to the N- and C-terminus of BioID, BioID2, and TurboID, respectively (Fig. 1a). These fusion proteins were expressed in *N. benthamiana* plants using the *Agrobacterium*-mediated infiltration method. Time course analysis indicated that these proteins are efficiently expressed at 36 h post infiltration (hpi) and reached the highest expression level at 48 hpi (Supplementary Fig. 1a, note: MYC-fused BioID was used in the time course analysis). Therefore, 36 h after agroinfiltration, we then infiltrated 200 µM biotin into the leaves and incubated for an additional 12 h at either room temperature (RT) or 37 °C. Immunoblot analysis indicated that BioID and BioID2 have labeling activity at both temperatures (Fig. 1b, upper panel, and Supplementary Fig. 1b). However, as previously reported^15,21^, labeling activity of BioID and BioID2 is higher at 37 °C than at RT (Fig. 1b, upper panel, and Supplementary Fig. 1b, upper panel). Immunoblot analysis with anti-MYC antibody showed no obvious changes in the expression levels of these proteins under different temperatures (Fig. 1b, middle panel and Supplementary Fig. 1b, middle panel). Interestingly, among the three tested biotin ligases, Citrine-TurboID had higher biotinylation activity compared to Citrine-BioID or Citrine-BioID2, either at 37 °C or at RT (Fig. 1b, upper panel, and Supplementary Fig. 1b, upper panel). Despite moderately decreased biotinylation level for TurboID at RT compared to 37 °C, it still generates significantly more biotinylated protein bands at RT than BioID or BioID2 at 37 °C (Fig. 1b, upper panel). These results indicated that although BioID, BioID2, and TurboID can promiscuously biotinylate proteins in plant cells, TurboID has the highest activity and the ability to efficiently label proteins at temperatures conducive to *in planta* experiments.

To determine the optimal concentration of exogenous biotin required for efficient labeling in plant cells, we tested biotinylation efficiency in leaves expressing Citrine-TurboID with a biotin gradient. The saturation point for biotinylation by Citrine-TurboID at 37 °C in *N. benthamiana* was reached with 200 µM biotin (Fig. 1c, upper panel). At RT, a comparable self-biotinylation level of Citrine-TurboID was achieved with the addition of 200–600 µM biotin and further application of more biotin (800 µM) slightly increased the amount of biotinylated proteins (Fig. 1c, upper panel, and Supplementary Fig. 1c). Immunoblotting with an anti-MYC antibody confirmed similar expression levels of Citrine-TurboID in all extracts (Fig. 1c, middle panel, and Supplementary Fig. 1c). These results indicated that 200 µM biotin is sufficient for TurboID to biotinylate the majority of itself as well as proximate proteins. In addition, similar analyses showed that 100–200 µM of exogenously applied biotin at 37 °C is sufficient for BioID- and BioID2-mediated biotinylation in plants (Supplementary Fig. 1c).

We also analyzed the effects of different incubation times on TurboID-mediated protein biotinylation. Our results showed that protein biotinylation by TurboID could be accomplished rapidly within 15 min after application of 200 µM biotin, either at 37 °C or RT (Fig. 1d, upper panels). Further increases in incubation time resulted in no apparent increase in biotinylation, suggesting that the saturation of protein biotinylation occurs by 15 min. Immunoblotting with anti-MYC antibody confirmed comparable Citrine-TurboID expression levels in all extracts (Fig. 1d, middle panel and Supplementary Fig. 2). In contrast, for BioID and BioID2, a longer incubation time, up to 24 h, and a higher temperature (37 °C) are required to achieve maximum biotinylation (Supplementary Fig. 1d). These results indicated that TurboID enables rapid proximity labeling in plant cells under room temperature conditions. Together, our results revealed that TurboID-based proximity labeling performs better in plants than BioID and BioID2. The ability to perform PL using TurboID at RT is advantageous for studies in plants as it avoids inducing an abiotic stress response. Furthermore, experiments at RT are ideal for investigating NLRs because many plant NLRs are temperature sensitive and fail to activate a defense response at higher temperatures.

### Establishment of procedures for PL in *N. benthamiana* plants

We optimized TurboID-based proximity labeling in *N. benthamiana* to enable identification of protein interactions partners (Supplementary Fig. 3). *N. benthamiana* plants were infiltrated with agrobacterium containing the biotin ligase-fusion construct and at 36 hpi biotin was infiltrated and the plants were incubated at RT for 6–12 h to allow labeling by the biotin ligases. Agroinfiltrated leaves were then pooled and ground in liquid nitrogen, followed by protein extraction in RIPA lysis buffer (see methods for details). Since free biotin remains in the protein extract, a desalting procedure was conducted to remove the free biotin from the protein extracts. This step is critical as immunoblot analysis of the affinity-purified proteins detected very few biotinylated proteins without desalting compared to desalted samples (Supplementary Fig. 4).

### TurboID efficiently labels a known N interactor Hsp90

To determine the activity of different biotin ligases in labeling a specific interactor of a target protein, we tested the known interaction between the NLR immune receptor N and Hsp90^13^. We generated fusion constructs of BioID-HA, BioID2-HA, and TurboID-HA to the full-length N protein as well as Hsp90 fused to MYC tag (Supplementary Fig. 5a). We co-infiltrated Hsp90 with the different N fusion constructs as well as empty vector (EV) negative control into the *N. benthamiana* leaves (Supplementary Fig. 5a, b). At 36 hpi, 200 μM biotin, in a 10 mM MgCl_2_ solution was infiltrated and plants were incubated at RT. Leaf samples were then collected 3 and 6 h after biotin infiltration. Leaf samples were ground and 700 mg tissue was subjected to protein extraction and desalting as described above (Supplementary Figs. 3 and 5b). Western blot analysis was performed using Streptavidin-HRP, anti-HA or anti-MYC antibodies, respectively, which confirmed that the fusion proteins were expressed in various infiltrated leaves (Supplementary Fig. 5c, panels ii and iii). Increased biotinylation was observed in protein samples from infiltrated leaves expressing gN-TurboID-HA and Hsp90-MYC compared to gN-BioID-HA and Hsp90-MYC or gN-BioID2-HA and Hsp90-MYC (Supplementary Fig. 5c, panel i).

To determine if Hsp90 is labeled in these samples, the desalted protein samples were subjected to Streptavidin pull-down, as described above, followed by western blot analysis. Results showed the production of more biotinylated protein bands for the samples from gN-TurboID-HA and Hsp90-MYC co-infiltrated leaves than that from either gN-BioID-HA and Hsp90-MYC or gN-BioID2-HA and Hsp90-MYC co-infiltrated leaves (Supplementary Fig. 5c, panel iv). Increased *cis*-biotinylation of N was observed in gN-TurboID-HA samples compared to gN-BioID-HA or gN-BioID2-HA (Supplementary Fig. 5c, panel v). In addition, a substantial amount of Hsp90-MYC was detected in gN-TurboID-HA (Supplementary Fig. 5c, panel vi). To further confirm these results, Hsp90-MYC immunoprecipitation (IP) products were analyzed. We observed stronger Streptavidin-HRP signal for the Hsp90-MYC-specific band in the gN-TurboID-HA and Hsp90-MYC samples than in either the gN-BioID-HA and Hsp90-MYC or gN-BioID2-HA and Hsp90-MYC samples (Supplementary Fig. 5d, upper panel). We also detected a band corresponding to gN-TurboID-HA only in the gN-TurboID-HA and Hsp90-MYC samples (Supplementary Fig. 5d, upper panel). The equal amounts of the enriched Hsp90-MYC proteins in the IP products were confirmed by immunoblot analyses with MYC antibodies (Supplementary Fig. 5d, bottom panel). These results together with the results shown in Fig. 1b, revealed that TurboID-based PL outperforms BioID and BioID2-based PL, not only at self-biotinylation and chance biotinylation, but also at the specific biotinylation of a known target protein.

### Identification of N NLR interacting proteins using TurboID

**Fig. 2:**
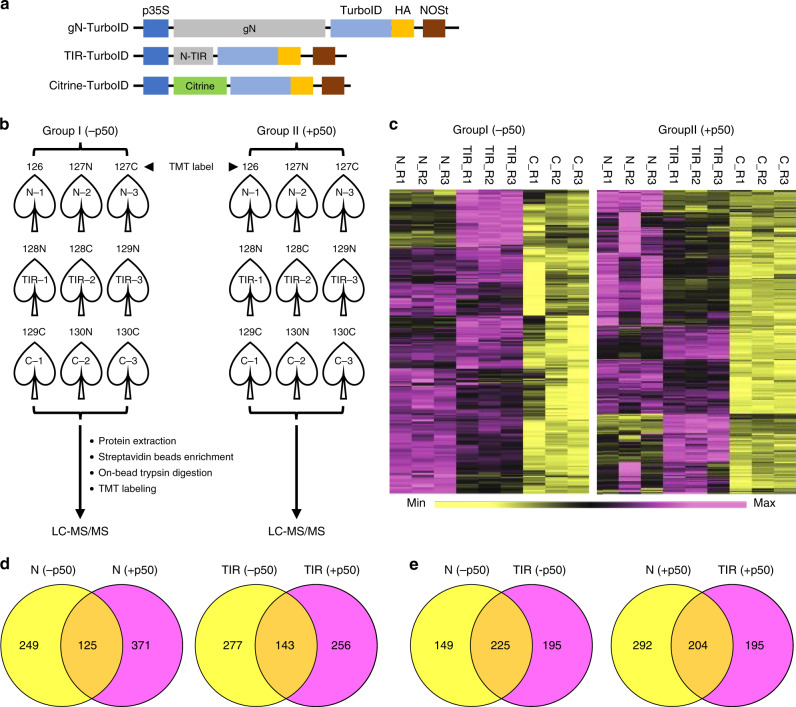
Identification of proximal and interacting proteins of N NLR immune receptor. **a** Schematic representation of the constructs used for the identification of proximal and interacting proteins of N. **b** Diagram of the experimental design and labeling conditions. TurboID fusions without p50 (Group I) or with p50 that is under the control of the β-estradiol inducible promoter (Group II) were co-infiltrated into *N. benthamiana* leaves. At 36 hpi, 200 µM biotin and 30 µM 17-β-estradiol were infiltrated into both groups of pre-infiltrated leaves. 12 h later, leaf cells were lysed and biotinylated proteins were enriched with streptavidin beads, digested by trypsin, and labeled with tandem mass tags (TMT). Each treatment consisted of three independent biological replicates. All 9 samples in Group I or Group II, respectively, were then independently combined within group and analyzed by LC-MS/MS. **c** Hierarchical clustering of the Group I and Group II significantly enriched interacting proteins. Enriched interactors exhibited a >1.5 fold enrichment over the Citrine control and a *q*-value less than 0.05. **d** Venn diagrams depicting the proteins that interact with gN (left) or N-TIR (right) in the absence or presence of p50. **e** Overlap in the proteins that interact with gN and/or N-TIR in the absence (left) or presence (right) of p50

Since TurboID-based PL outperforms other PL approaches, we used TurboID to identify the proximal and interacting proteins of N. In addition to using the full-length gN-TurboID-3xHA fusion described above, we also generated a construct with just the TIR domain of N fused to TurboID-3xHA (Fig. 2a). For these experiments, Citrine-fused TurboID served as the negative control (Fig. 2a). We confirmed that gN-TurboID-3xHA is functional by assessing its ability to induce HR-PCD when coexpressed with TMV p50 effector (Supplementary Fig. 6a). In contrast, the expression of N-TIR-TurboID-3xHA and Citrine-TurboID-3xHA with p50 did not result in HR-PCD, as expected (Supplementary Fig. 6a). Next, to identify proteins that associate with N in the absence and/or presence of p50 effector, we infiltrated the various TurboID-fusions (Fig. 2a), with or without co-infiltration of p50 fused to tagRFP under the control of *β*-estradiol inducible promoter^27^ (XVE::tRFP-p50), into *N. benthamiana* leaves. We used an inducible system to express p50 so that we could initiate *N*-mediated defense response at the time of addition of biotin for PL. About 36 hpi of the different TurboID-fusions, with or without XVE::tRFP-p50, biotin and estradiol were infiltrated to launch protein biotinylation concomitantly with the p50-mediated immune response. We confirmed, expression of TurboID-fusion proteins as well as the tRFP-p50 by immunoblotting using antibodies against HA (Supplementary Figs. 6b, middle panels, and Fig. 4c, panel ii) and tRFP (Supplementary Fig. 6b, lower panels), respectively. Immunoblot analysis using the HRP-conjugated streptavidin showed multiple biotinylated protein bands in protein extracts from different samples (Supplementary Fig. 6b, upper panels). We noted that *cis*-biotinylation of both the gN-TurboID and TIR-TurboID proteins were relatively weak compared to the control citrine-TurboID (also see Supplementary Figs. 5c, panel i and  7). This may be due to the distinct properties of the target proteins, which have varying effects on the *cis*-biotinylation by the fused biotin ligase. Consistent with this notion, a previous study reported that HopF2 fused to BioID showed weaker *cis*-biotinylation when compared to BioID alone^23^.

To quantify the relative abundance of PL proteins, we prepared and analyzed Group I samples without p50 and Group II samples with p50 (Fig. 2b). TurboID labeling was performed for 12 h by infiltration of biotin. Expression of tRFP-p50 was induced by the addition of estradiol at the same time as the addition of biotin. Although the TurboID-based PL could be completed in just 15–30 min, concomitant activation of the *N*-mediated defense response requires a longer time. Therefore, we employed 12 h of incubation time to coordinate the activation of *N*-mediated defense and labeling of N-interacting proteins. After 12 h, plant leaves were harvested, ground, and lysed. Biotinylated proteins were enriched from the different protein extracts according to the procedures shown in Supplementary Fig. 3 and an aliquot was analyzed by immunoblotting to confirm their usability for subsequent LC-MS/MS analysis (Supplementary Fig. 7). The enriched proteins were removed from the streptavidin beads using on-bead digestion and the resulting peptides were chemically labeled with isotopically distinct tandem mass tag (TMT) labels (Fig. 2b) (see “Methods” section for details)^28,29^. TMT labeled samples were pooled and 15 µg were analyzed by 2 dimensional (2D) LC-MS/MS. 2D-LC-MS/MS was performed using online strong cation exchange (SCX) as the first dimension and low pH reverse-phase as the second dimension to deliver peptides to a Q Exactive Plus mass spectrometer^30–32^. Finally, we used MaxQuant^33^ to identify peptides and quantify the relative amount of each affinity purified protein within Group I and within Group II.

We identified 3198 and 3262 proteins in Group I and Group II, respectively, that had TMT intensity values greater than zero (Supplementary Data 1 and 2). We examined reproducibility of the data and found high Pearson correlation values among the biological replicates of both Group I (*r* > 0.97) and Group II (*r* > 0.99) datasets. Next, we identified proteins that interact with either full-length gN or the N-TIR domain, relative to the Citrine control. To determine enrichment (i.e., interactors) we used the software package Perseus^34^ to calculate two-sample *t*-tests and perform permutation-based false discovery rate (FDR) correction (*q*-value). We designated proteins as enriched interactors if they had a *q*-value less than 0.05 and a greater than 1.5-fold enrichment over the Citrine control. These analyses revealed 374 (gN) and 420 (N-TIR) enriched interactors in the absence of p50 (Fig. 2c, Supplementary Fig. 8a, b and Supplementary Data 1). Additionally, 496 (gN) and 399 (N-TIR) proteins were identified as enriched interactors in the presence of p50 (Fig. 2c, Supplementary Fig. 8c, d and Supplementary Data 2). Furthermore, the interaction of numerous proteins with N or N-TIR is dependent on the presence of p50. Specifically, 249 (gN) and 277 (N-TIR) proteins interact in the absence of p50 whereas 371 (gN) and 256 (N-TIR) proteins were enriched only in the presence of p50 (Fig. 2d). Additionally, there are many proteins whose interaction with N is either dependent or independent of the TIR domain (Fig. 2e). Together, these data represented a robust set of putative N-interacting proteins for functional characterization.

### Functional analyses of N NLR interacting proteins

To determine whether the N NLR interacting proteins identified from TurboID-based proximity labeling function in the *N*-mediated resistance to TMV, we selected 10 proteins (Supplementary Table 1) from the MS dataset for the subsequent genetic screening. For this, we selected proteins that spanned the range of fold-enrichment values. Specifically, we selected proteins that were just over our 1.5 fold-enrichment cutoff to proteins among those exhibiting the most extreme enrichment. These proteins include Niben101Scf00410g02014.1 (*Nb*UBR7, mammalian homolog of a putative E3 ubiquitin ligase UBR7) and Niben101Scf03903g02005.1 (*Nb*PPR, pentatricopeptide repeat-containing protein), which showed highest fold change score in the MS data of Group I in the absence of p50 (Supplementary Data 1 and Supplementary Table 1). Interestingly, these proteins were not detected in the MS data of Group II in the presence of p50 effector (Supplementary Data 2 and Supplementary Table 1).

Recent evidence suggests that NLRs may function in pairs or in a network^35–37^. In this model, NLRs that recognize the pathogen (sensor NLR) require function of another NLR (helper NLR)^35–37^. Interestingly, our MS data identified five NLRs that interact with N. Among them, Niben101Scf09716g02001.1 (*Nb*R1B-14, putative late blight resistance protein homolog R1B-14) and Niben101Scf14194g00002.1 (*Nb*RGA1, putative disease resistance protein RGA1-like) showed >1.5 fold-enrichment in both Group I and Group II, and also in both N and N-TIR samples. In contrast, Niben101Scf06889g00007.1 (*Nb*RPP13L, putative disease resistance RPP13-like) showed >1.5 fold-enrichment only in Group II, whereas Niben101Scf02248g01001.1 (*Nb*R1A-3, late blight resistance protein homolog R1A-3) exhibited enrichment only in Group I. For Niben101Scf05619g00011.1 (NbRGA3, putative disease resistance protein RGA3), significant fold change was observed only N-TIR samples in Group II (Supplementary Data 1 and 2; Supplementary Table 1). In addition to these NLRs, we also included NRC2a (Niben101Scf02133g00003.1) NLR, which is below the significance cutoff level in our MS dataset (Supplementary Data 1 and 2; Supplementary Table 1), as a negative control. NRC2a, has been shown to function as a helper NLR^36,38^ for various sensor NLRs, but not for N.

We also included proteins like Niben101Scf16939g00004.1 (eukaryotic aspartyl protease family protein, *Nb*Asp), Niben101Scf02819g02011.1 (Topless-related protein 4, *Nb*TPL4), and transcription factor MYC2 identified from tobacco database search (*Nb*MYC2, *N. tabacum* ID: A0A1S4BAV2) for functional analyses because these proteins also showed significant fold change in the MS dataset (Supplementary Data 1 and 2; Supplementary Table 1) and they have been reported to be involved in the plant immune responses^39–41^.

To determine the function of selected proteins in *N*-mediated resistance to TMV, we used our well-established and widely used *Tobacco rattle virus* (TRV)-based virus-induced gene silencing (VIGS)^42,43^ system to knock-down the expression of selected genes in transgenic *N*-containing *N. benthamiana* plants^42^. TRV-N that is designed to silence the *N* gene, as well as the empty VIGS vector which we have previously described^42,43^, were also inoculated onto the plants to serve as the positive and negative controls, respectively. After ~10 days of silencing, plants were infected with TMV-U1 and then monitored for the resistance to TMV. Prior to TMV infection, silenced plants exhibited no obvious altered plant growth phenotype compared to the TRV-vector control (Supplementary Fig. 9).

**Fig. 3:**
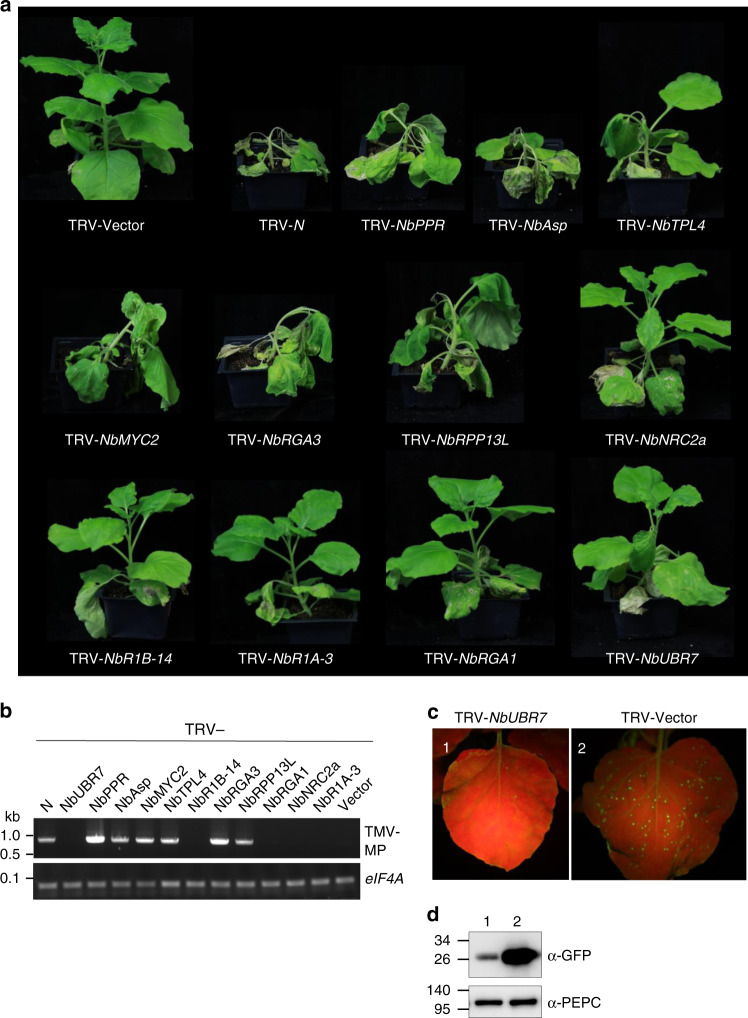
Genetic screening of the function of candidate N NLR immune receptor interacting proteins in *N*-mediated resistance to TMV. **a** Phenotypic observation of the TMV-U1-incoluted *N*-containing transgenic *N. benthamiana* plants after being silenced with various target genes as indicted. Various recombinant TRV vectors containing different gene fragments were inoculated onto the *N*-containing transgenic *N. benthamiana* plants. 10 days later, TMV-U1 was rub-inoculated onto the upper leaves. Photographs were taken at 7 days after TMV inoculation and representative results are shown. These silencing experiments were repeated 2–3 times. **b** Analysis of the TMV RNA corresponding to the movement protein coding region in the upper uninoculated leaves by RT-PCR (TMV-MP, top panel). eIF4A was used as an internal control to validate the equal amount of total RNA used for RT-PCR (bottom panel). **c** Silencing of *Nb*UBR7 enhances *N*-mediated resistance to TMV. TMV-U1-GFP was rub-inoculated onto vector control or the *Nb*UBR7-silenced *N*-containing transgenic *N. benthamiana* leaves. Photographs were taken under UV light at 5 days post inoculation and representative results are shown (upper panels). **d** The expression level of GFP in the inoculated leaves was examined by western blot analysis using antibodies against GFP (top panel). PEPC served as loading control for equal protein loading (bottom panel). The molecular weight size markers in kDa are indicated at the left of each panel. Source data are provided as a Source Data file

In the VIGS-vector control plants, TMV-U1 was contained to the infection site and the upper uninoculated leaves remained healthy (Fig. 3a). However, silencing of the *NbPPR*, *NbAsp*, *NbMYC2*, *NbTPL4*, *NbRGA3*, and *NbRPP13L* lead to the collapse of the inoculated leaves and eventual death of the whole plant, which is similar to that of the *N*-silenced plants (Fig. 3a). In contrast, plants silenced in *NbNRC2a*, *NbR1B-14*, *NbR1A-3*, *NbRGA1*, and *NbUBR7* showed similar phenotype to that of the VIGS-vector control (Fig. 3a). We monitored the presence of TMV in the upper uninoculated leaves by RT-PCR. Our results showed accumulation of TMV in the upper uninoculated leaves in *N*-, *NbPPR*-, *NbAsp*-, *NbMYC2*-, *NbTPL4*-, *NbRGA3*-, and *NbRPP13L*-slienced plants, but not in the VIGS-vector control plants or other genes silenced plants (Fig. 3b). The downregulation of target genes expression in the various recombinant TRV vector-inoculated plants was also confirmed by reverse transcription quantitative real-time PCR (RT-qPCR) (Supplementary Fig. 11). These results indicate that *NbPPR*, *NbAsp*, *NbMYC2*, *NbTPL4*, *NbRGA3* and *NbRPP13L* are required for *N*-mediated defense response to restrict TMV to the infection site.

Intriguingly, when TMV-U1 tagged with GFP (TMV-U1-GFP) was inoculated onto the *NbUBR7*-silenced leaves, the number of fluorescent spots was remarkably decreased in comparison to that of the VIGS-vector control plants (Fig. 3c). Immunoblot analysis showed a lower level of TMV-U1-GFP in the inoculated leaves of *NbUBR7*-silenced leaves compared to the VIGS-vector-inoculated control leaves (Fig. 3d). These results indicated that silencing of UBR7 enhances *N*-mediated resistance to TMV, and suggest that UBR7 functions as a negative regulator of N.

To further validate the identified interactors of N, we performed Bimolecular Fluorescence Complementation (BiFC) assay^44^ with some of the above-discussed candidate interactors. For this, we generated candidate proteins fused to C-terminal of citrine (candidate protein^YC^) and used our previously described gN fused to the N-terminal 155 amino acid residues of citrine (gN^YN^)^12^. We coexpressed gN^YN^ and candidate protein^YC^ in *N. benthamiana* plants followed by confocal microscopy. Co-expression of gN^YN^ with NbUBR7^YC^, NbPPR^YC^, NbTPR^YC^, NbRPP13L^YC^, NbRGA1^YC^ or NbRLK6^YC^ reconstituted citrine fluorescence but not when the NRC2a negative control was coexpressed with gN^YN^ (Supplementary Fig. 10a). We found no BiFC signal when gN^YN^ was coexpressed with NbR1B-14^YC^ (Supplementary Fig. 10a). Fusion protein expression was confirmed by western blot analyses (Supplementary Fig. 10b). These results indicate that 6 out of 7 of the tested candidate proteins interacted with N in the BiFC assay.

### NbUBR7 interacts with TIR domain of N in vivo and in vitro

**Fig. 4:**
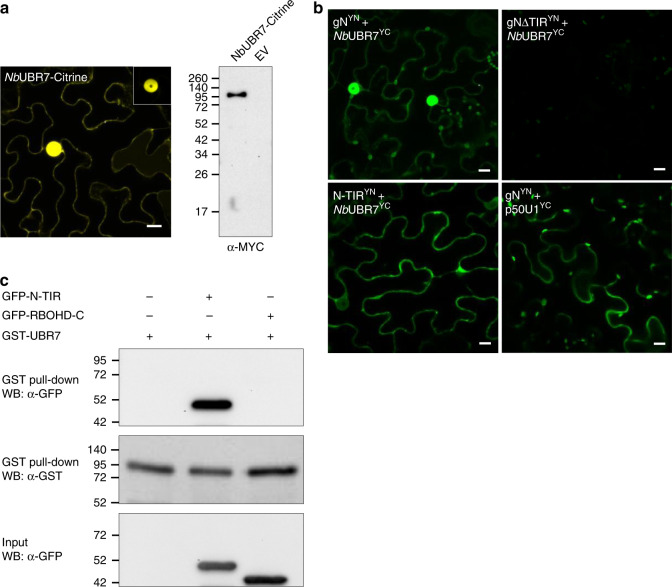
Analysis of the interaction between N and *Nb*UBR7 in vivo and in vitro. **a** Subcellular localization of *Nb*UBR7. *Nb*UBR7 was fused to citrine (*Nb*UBR7-citrine) followed by agroinfiltration. Confocal analysis was performed at 2 dpi. NbUBR7 is present in the cytoplasm and in the nucleus (left panel). The rectangular region on top right of the image indicated a lower gain value image to confirm the nuclear localization of *Nb*UBR7. Scale bar represents 10 µm. NbUBR7 expression was confirmed by western blot analysis using antibodies against MYC tag (right panel). Empty vector served as the control. The molecular weight size markers in kDa are indicated at the left. **b** BiFC analysis of the interaction between *Nb*UBR7 and N as well as its different domains. *Nb*UBR7 fused to C-terminus of citrine (*Nb*UBR7^YC^) was coexpressed with native promoter-driven full-length N (gN), TIR domain deleted N (gNΔTIR) or TIR domain alone (N-TIR) fused to N-terminus of citrine in the *N. benthamiana* leaves. Confocal analysis was performed at 2 dpi. gN^YN^ and p50U1^YC^ served as the positive control. Scale bars = 10 µm. **c** In vitro GST-pull down assay to examine the interaction between *Nb*UBR7 and the TIR domain of N. The purified GFP-tagged TIR domain of N or C-terminal region of the respiratory burst oxidase homolog D (RBOHD) were incubated with GST-tagged *Nb*UBR7. After being pulled-down with glutathione-sepharose beads, the proteins were detected by Western blot (WB) with anti-GFP or anti-GST antibodies. Source data are provided as a Source Data file

Since we know very little about negative regulators of NLRs, we next investigated the molecular mechanism underlying UBR7-mediated regulation of the N protein. UBR7 belongs to the UBR-box protein family that is highly conserved across mammals, flies, plants, and yeast^45,46^. *N. benthamiana* UBR7 is highly similar to the mammalian UBR7 and contains the typical UBR box (Supplementary Fig. 12). To determine the localization of *Nb*UBR7, we fused *Nb*UBR7 to citrine and performed confocal microscopy. Our results showed that the *Nb*UBR7 is a nucleocytoplasmic protein (Fig. 4a, left panel), which is similar to the previously described nucleocytoplasmic localization of the N protein^11,12^. The expression of *Nb*UBR7-citrine was also confirmed by immunoblot analysis (Fig. 4a, right panel). To further validate the interaction between *Nb*UBR7 and N, we used BiFC assay. Co-expression of gN^YN^ and UBR7^YC^ reconstituted the citrine fluorescence (Fig. 4b, top left panel), and the reconstituted citrine fluorescence signal was observed in the nucleus and in the cytoplasm (Fig. 4b, top left panel). Expression of gN^YN^ and *Nb*UBR7^YC^ was confirmed by immunoblot analysis (Supplementary Fig. 13a). We next tested the interaction between *Nb*UBR7 and a previously described mutant form of N, which has a deletion of the TIR domain (gNΔTIR)^12^. Our BiFC results showed that coexpression of gNΔTIR^YN^ and NbUBR7^YC^ failed to reconstitute the citrine fluorescence (Fig. 4b, top right panel). However, co-expression of TIR domain alone (TIR^YN^) with *Nb*UBR7^YC^ leads to the reconstitution of citrine fluorescence (Fig. 4b, bottom left panel). Expression of *Nb*UBR7^YC^, gNΔTIR^YN^, and TIR^YN^ was confirmed by immunoblot analysis (Supplementary Fig. 13a-b). These results indicated that NbUBR7 associates with the TIR domain of N.

To further confirm the interaction between N TIR domain and *Nb*UBR7, we performed GST pull-down assays using recombinant proteins purified from *E. coli*. Our results showed that GST-fused *Nb*UBR7 specifically pulled-down the GFP-fused TIR domain of N, but not the GFP-fused C-terminal region of the respiratory burst oxidase homolog D **(**RBOHD) control (Fig. 4c). Taken together, these results demonstrate that *Nb*UBR7 interacts with the TIR domain of N both in vivo and in vitro.

### *Nb*UBR7 modulates N stability and negatively regulates defense

**Fig. 5:**
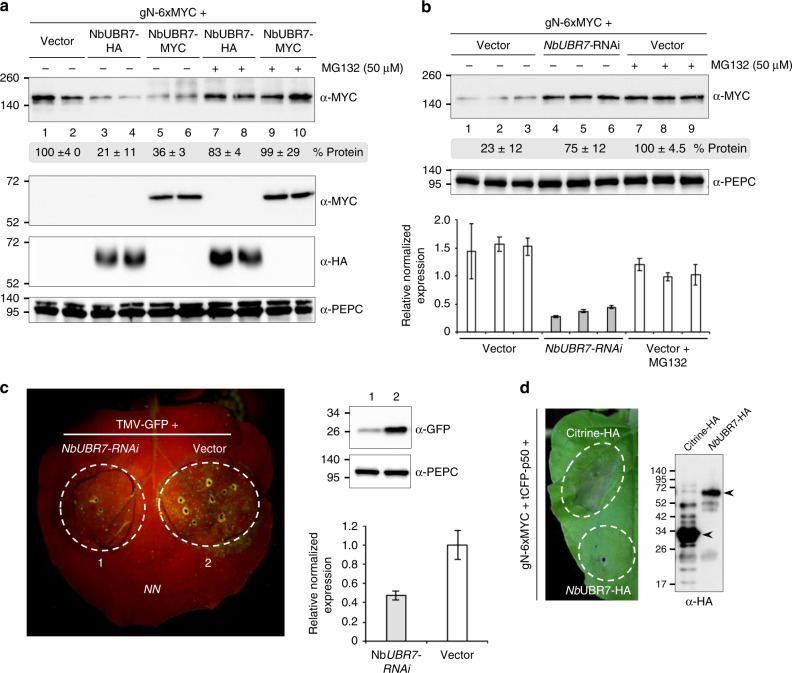
NbUBR7 modulates N protein level and functions as a negative regulator of *N*-mediated defense. **a** *Nb*UBR7 overexpression reduced the stability of N in a proteasome-dependent manner. Agrobacterium containing expression sets consisting of an empty vector control (vector) or the agrobacterium containing the expression cassette of *Nb*UBR7-HA or *Nb*UBR7-MYC were infiltrated into *N. benthamiana* leaves followed by infiltration of MYC-tagged gN 24 later. 36 h after infiltration of gN, 50 µM proteasome inhibitor MG132 (+MG132) or the equal concentration of DMSO (−MG132) were further infiltrated into the pre-infiltrated leaves. Infiltrated leaf tissues were collected 12 h after DMSO or MG132 treatment and analyzed by western blot analysis. Antibodies used for the western blot analysis were indicated on the right of each panel. Equal protein loading is assessed by the similar amounts of PEPC protein. Molecular weight size markers in kDa are indicated on the left. Two independent samples (*n* = 2) for each treatment were analyzed in parallel. Band intensity was measured by Image J software and normalized to the PEPC protein control. Numbers below the top panel indicate the relative quantification of the corresponding band intensity, of which the empty vector control group was set to 100% [“±” indicates standard deviation (SD) of the mean]. **b** Double stranded hairpin-mediated silencing of *Nb*UBR7 (*Nb*UBR7-RNAi) enhances the stability of N. Empty vector control (vector) or the agrobacterium containing the hairpin *Nb*UBR7 were infiltrated into the *N. benthamiana* leaves followed by infiltration of MYC-tagged N at 24 hpi. 36 h later, DMSO (-MG132) or MG132 (MG132) was then infiltrated into the pre-infiltrated leaves as described above. Infiltrated leaf tissues were collected 12 h after DMSO or MG132 treatment and analyzed by western blot. Three independent replicates (*n* = 3) were carried out for each treatment. Numbers below the top panel indicate the relative quantification of the corresponding band intensity, of which the MG132-treated empty vector control was set to 100% (“±” indicates SD). Equal protein loading is assessed by the similar amounts of PEPC protein (middle panel). Molecular weight size markers in kDa are indicated on the left. The downregulation of *Nb*UBR7 in the infiltrated leaves were also confirmed by RT-qPCR (bottom panel). Data from three biological replicates were combined and values are shown as mean ± SD. **c** RNAi of *Nb*UBR7 enhances the *N*-mediated resistance to TMV. Different regions of the leaf of *N*-containing transgenic *N. benthamiana* were first agroinfiltrated with hairpin *Nb*UBR7 (1) and the control empty vector (2), respectively. 24 h later, TMV-U1-GFP was agroinfiltrated into the previously infiltrated regions. Photographs were taken under UV light at 5 dpi and representative results are shown (left panel). Leaf samples from the region 1 and 2 were then harvested and subjected to western blot analysis using the antibodies against GFP or PEPC (top right panels). RT-qPCR was performed to confirm the downregulation of *Nb*UBR7 in the infiltrated region of the leaves (bottom right panel). Data from three biological replicates were combined and values are shown as mean ± SD. **d** Overexpression of *Nb*UBR7 inhibits p50 effector-induced HR-PCD. Different leaf regions were infiltrated with MYC-tagged N (gN-6xMYC), TagCFP-tagged p50 (tCFP-p50) together with HA-tagged *Nb*UBR7 (*Nb*UBR7-HA) or citrine (Citrine-HA) control. Photographs were taken at 6 dpi and representative results are shown (left panel). The expression of citrine-HA or *Nb*UBR7-HA was confirmed by Western blot analysis using anti-HA antibody (right panel). Arrowheads indicate the specific band of different HA fusions. Source data are provided as a Source Data file

To further characterize the functional role of *Nb*UBR7 in *N*-mediated resistance to TMV, HA- or MYC-tagged UBR7 together with N were coexpressed in *N. benthamiana* leaves. Immunoblot analysis showed that overexpression of either *Nb*UBR7-HA or *Nb*UBR7-MYC leads to significant reduction of N accumulation compared to the vector control (Fig. 5a, top panel). Interestingly, the reduced accumulation of N due to *Nb*UBR7 overexpression could be rescued with the addition of the proteasome inhibitor MG132, suggesting that the UBR7-mediated accumulation of N is proteasome dependent (Fig. 5a, top panel). Furthermore, when *Nb*UBR7 was silenced in the leaves by NbUBR7 hairpin construct (*NbUBR7*-RNAi), the N expression level was increased compared to the unsilenced leaves inoculated with an empty vector (Fig. 5b, top panel). Downregulation of *NbUBR7* was validated by RT-qPCR (Fig. 5b, bottom panel). Consistently, an increase in N accumulation was observed in the presence of MG132 (Fig. 5b, top panel), which is in-line with the results shown in Fig. 5a. Moreover, *NbUBR7*-RNAi in the *N*-containing transgenic *N. benthamiana* plants dramatically impaired the fluorescent intensity after rub-inoculation of TMV-U1-GFP (Fig. 5c, left panel). Immunoblot analysis confirmed the lower accumulation of TMV-U1-GFP in comparison to that of the control (Fig. 5c, right top panel). Downregulation of *Nb*UBR7 was confirmed by RT-qPCR (Fig. 5c, right bottom panel). To determine the effect on HR-PCD, N and the TMV p50 effector together with citrine or UBR7 were coexpressed in the *N. benthamiana* leaves. Our results showed that overexpression of the *Nb*UBR7 greatly compromised *N*-mediated HR-PCD to p50 compared to the citrine-HA control (Fig. 5d, left panel). Protein expression in these experiments was confirmed by immunoblot analysis (Fig. 5d, right panel). Together, these results demonstrate that *Nb*UBR7 regulates the stability of N protein and negatively regulates the *N*-mediated defense response.

### TMV p50 effector disrupts N-*Nb*UBR7 interaction

**Fig. 6:**
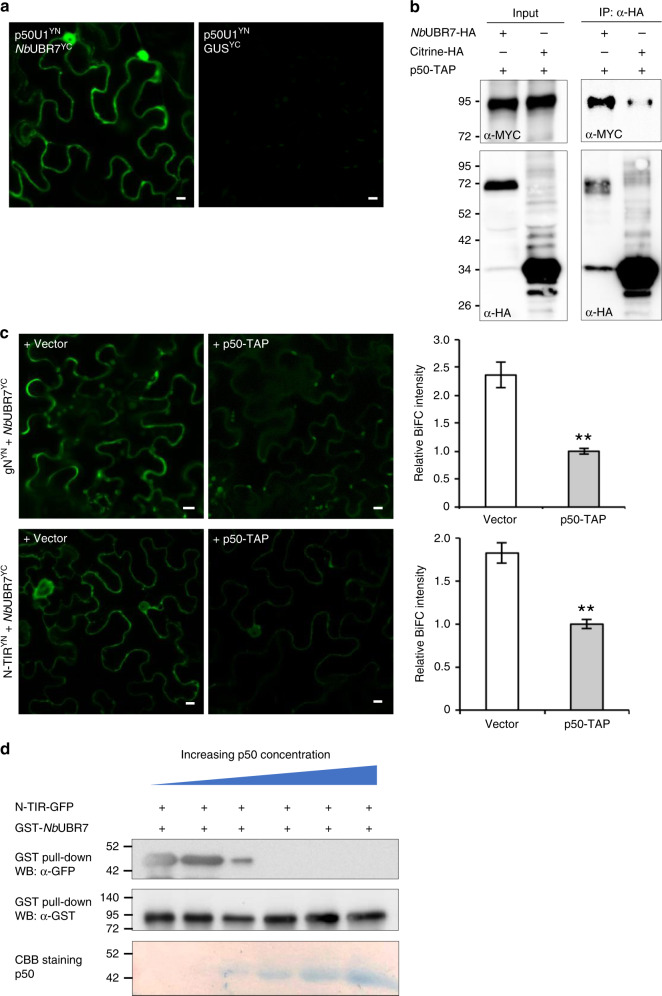
TMV p50 effector interferes with the interaction between *Nb*UBR7 and the TIR domain of N. **a** BiFC analysis of the interaction between p50U1 and *Nb*UBR7. The constructs used for co-infiltration were indicated on the upper left of each panel. GUS^YC^ served as a control for the specificity of associations involving p50U1^YN^. Scale bars = 10 µm. The expression of p50U1^YN^ and NbUBR7^YC^ were confirmed by western blot analysis (Supplementary Fig. 13b). **b** Co-IP analysis of the interaction between p50U1 and *Nb*UBR7. HA-tagged UBR7 or citrine was coexpressed with TAP-tagged p50 in the *N. benthamiana* leaves. Protein extracts from the infiltrated leaf tissues were incubated with anti-HA antibody-conjugated agarose beads. Protein extracts (input) or immunoprecipitated (IP) complexes were separated by SDS-PAGE and probed with anti-MYC or anti-HA antibodies. p50-TAP was co-precipitated with *Nb*UBR7-HA, but not with Citrine-HA. Molecular size markers in kDa are indicated on the left. **c** BiFC competition analysis showed the inhibitory effect of p50 on the interactions between *Nb*UBR7 and N or *Nb*UBR7 and TIR domain. BiFC constructs together with the empty vector control or the p50-TAP were coinfiltrated into the *N. benthamiana* leaves. Confocal analysis was performed at 2 dpi. Scale bars = 10 µm. Reconstituted citrine fluorescence intensity was quantified using the Image J software and p50-TAP-infitrated groups were used as the normalizer (=1). Error bars represent standard deviation from the mean (*n* = 3). Asterisk indicates statistically significant difference between vector and p50-TAP groups (Student’s *t*-test, **P *=* 0.004 for upper right panel and **P = 0.007 for bottom right panel). The expression of *Nb*UBR7^YC^ and p50-TAP were confirmed by western blot analysis (Supplementary Fig. 13c). **d** In vitro competitive GST pull-down assay showed the binding of *Nb*UBR7 to TIR domain was inhibited with the addition of an increasing amount of p50. GST-tagged *Nb*UBR7 or GFP-tagged TIR domain were expressed and purified from *E. coli*. These two proteins were pulled down with glutathione-agarose in the presence of an increasing concentration of p50 (0.1, 1, 2, 4, 8 µg). Pull-down samples were analyzed by western blot (WB) with anti-GST or anti-GFP antibodies. CBB staining of the increasing amounts of p50 protein is shown in the bottom panel. Source data are provided as a Source Data file

TMV infection or the expression of p50 has been shown to enhance the accumulation of N protein^11,12,14^. Since UBR7 is not present in our Group II MS dataset that was generated in the presence of p50 effector and our above-described data indicate that *Nb*UBR7 negatively regulate the stability of N, we investigated the relationship among N, p50, and *Nb*UBR7. We first performed BiFC analysis to test whether p50 interacts with *Nb*UBR7. Our results showed that co-expression of p50^YN^ and *Nb*UBR7^YC^ reconstituted citrine fluorescence compared to the control GUS^YC^ and p50^YN^ (Fig. 6a). Expression of the *Nb*UBR7^YC^ and p50^YN^ was confirmed by immunoblot analyses (Supplementary Fig. 13c). Co-immunoprecipitation (co-IP) analysis was carried out in *N. benthamiana* leaves to further confirm the interaction between p50 and *Nb*UBR7. TAP-tagged p50 efficiently co-IPed *Nb*UBR7-HA compared to the control citrine-HA (Fig. 6b), indicating that p50 associates with *Nb*UBR7 in vivo. Interestingly, BiFC analysis showed that p50 expression significantly impaired the intensity of reconstituted citrine fluorescence for either gN^YN^ with *Nb*UBR7^YC^ or the TIR^YN^ with *Nb*UBR7^YC^ (Fig. 6c, middle panels), compared to the empty vector control (Fig. 6c, left panels). Quantification of the BiFC fluorescent intensity further confirmed that p50 interfered with the interaction between the UBR7 and full-length N as well as with the TIR domain (Fig. 6c, right panels). Expression of the *Nb*UBR7^YC^ and p50-TAP in these experiments was confirmed by immunoblot analyses (Supplementary Fig. 13d). Furthermore, in vitro GST-pull down assays, with increasing amounts of p50, were performed to evaluate the impact of p50 on the interaction between the TIR domain and *Nb*UBR7. Interestingly, binding of TIR domain to *Nb*UBR7 was abolished with an increased amount of p50 (Fig. 6d). These results indicate that the TMV p50 effector disrupts UBR7-N interaction, resulting in enhanced stability of N and induction of defense response.

## Discussion

Biotin ligase-based PL has emerged as a powerful tool for probing target protein interactomes in a wide variety of species, including mammalian cells^15–17^, unicellular eukaryotes^47^, and some model organisms such as flies and worms^20^. Compared to traditional yeast two-hybrid (Y2H) and AP-MS methods, PL is ideal for identification of low-affinity, transient protein-protein interactions (PPIs), and/or insoluble protein structures in the native cellular environment^15–17^. Using PL, many components of subcellular organelles and regulators that function in a variety of cellular processes have been identified^16^. However, the application of PL in plants is at infancy with only three reports of BioID-based PL in plants^22–24^. Previous PL tools (BioID and BioID2) suffer from slow kinetics and a high required temperature for the in vivo labeling reaction. The recently developed TurboID-based PL bypassed many limitations of BioID system^20^. Thus, we tested whether the new TurboID system performs better than the BioID and BioID2 in plants. Also, we compared several parameters of these three biotin ligases including the temperature, the incubation time, and the amount of biotin. Our results indicated that both the temperature and the incubation time required for TurboID-based PL is remarkably superior to that of BioID and BioID2 in plants and highlight the great potential of TurboID for identification of PPIs in plant. We then used TurboID-based PL to identify new regulators of N NLR immune receptor-mediated immunity.

We also examined BioID, BioID2, and TurboID fusions to N in their ability to biotinylate Hsp90, a known N interacting protein^13^. Our results showed that the TurboID outperformed the other biotin ligases in its ability to PL Hsp90 (Supplementary Fig. 5). Although 15 min is sufficient for self- or *cis*-biotinylation and PL saturation of citrine-TurboID (Fig. 1d), greater PL was observed between N and Hsp90 at 6 h than at 3 h (Supplementary Fig. 5). Some of the reasons that could lead to differences in the optimal condition for the self-biotinylation and PL saturation are the expression level of the interactors and the time points for the occurrence of the interaction between the TurboID-fused protein and its interactors during a dynamic cellular event (e.g., *N*-mediated defense response during TMV infection). Thus, from a technical point, it is not possible to determine one optimal condition that will capture all interacting partners of a target protein of interest using a PL approach. Despite these caveats, our optimization of the procedure for PL with the Citrine-TurboID still provides an important basis for the future use of the TurboID-based PL in plants, as at it revealed the minimal time and biotin concentration required for the TurboID-based PL to occur at room temperature.

It should also be noted that higher activity and rapid proximity labeling of TurboID might lead to non-specific labeling during PL^15,20,21^. Although we cannot exclude the possibility that biotinylation of proteins over the long TurboID labeling periods may potentially impact protein function, biotin itself is not toxic^21^. For PL of those proteins with no need for conditional expression or long time to see their effects, a short period of time for PL, such as 15 min to 3 h after biotin infiltration, should be sufficient and preferred.

In our study, we used the XVE inducible system to conditionally express TMV-p50 with an aim to capture N interactors during the recognition of p50 by N (group II dataset). The XVE system rapidly responds to estradiol^27^, which was co-infiltrated with biotin. Therefore, there is a narrow time window for interaction with N, in the presence of biotin and absence of p50. Thus, the majority of labeling of N-proximal proteins in group II will occur when p50 is present, leading to many of the observed differences between group I and group II N-interactors in our study.

As previously described, specificity of the resulting proteomic dataset is improved by inclusion of appropriate controls designed to eliminate false-positives. For example, during the PL of N, we used citrine-fused to TurboID as a control for subsequent subtraction of the background proteins from the N protein interactors group. Furthermore, the advantage of multiplexing with isobaric tags is that we are able to quantify relative enrichment over the control, in the same MS run. Thus, even if N is partially cleaved from TurboID in vivo (and not degraded/released in vitro during extraction and heating), resulting in non-specific labeling by the “free” TruboID” (Supplementary Fig. 5c), biotinylated proteins that are not enriched relative to the control TurboID are filtered out in our analyses. Furthermore, it is known that the proximity labeling approaches (BioID/APEX) for subcellular and interaction proteomics yield non-specific hits and that quantitative proteomics approaches with controls are necessary to remove false positives^48^. In addition, previous proximity labeling studies based on either APEX or BioID/BioID2 approaches identified 9–36% positive enrichment over controls^49–51^. While it is challenging to directly compare false positive enrichment rates between studies using different methods of quantification (e.g., label-free verses isotopic labeling), reports from previous studies are generally consistent with our results that found 12–15% enriched interacting proteins, depending on the bait. Furthermore, as PL produces a “contour map” of labeling that can extend beyond the direct interaction sphere of the target protein^52^, enriched proteins may directly or indirectly interact with the target, or may just be in the vicinity without functional association. Hence, further genetic and biochemical analyses are required to validate and further study the proteomic hits. In our study, several candidate N interacting proteins, which spanned the range of fold-enrichment values in the MS dataset, were further validated using the BiFC approach. We found that 85% (6 of 7) of the tested candidates showed positive BiFC signal with N, in vivo, indicating the high efficiency of using TurboID-based PL for identifying bona fide interactors of a target protein. We also noted that RPP13L is enriched in the p50-treated samples, whereas BiFC signals between RPP13L and N was observed in the absence of p50. It is possible that RPP13L may be induced during p50 recognition or the N-RPP13L interaction may be stabilized in the presence of p50 and hence in the proximity labeling experiment, it was observed only in the presence of p50. In BiFC, enhanced level of expression of RPP13L is sufficient to interact with N. In summary, TurboID-based PL complements the traditional methods to identify the PPIs and provides an additional tool for robust and facile identification of interaction partners of a target protein in plants.

NLR immune receptors play a crucial role in mediating the defense response in both plants and animals, but the molecular mechanism underlying NLR-mediated immune signaling remains largely unknown^53^. Due to the tight control of NLR expression and dynamic nature of NLR-mediated immune responses, it has been difficult to identify the interacting proteins using traditional methods. Considering the advantage of PL in identifying PPIs, we used TurboID-based PL to identify the interacting proteins of a model plant NLR, N. Quantitative proteomic analysis of the enriched biotinylated proteins generated from TurboID-fused N identified a number of proteins that may associate with N. Our analyses identified Hsp90^13^, COP9 signalosome complex^54^, and protein disulfide isomerases (PDIs)^55^ which have been shown to be required for *N*-mediated resistance to TMV (Supplementary Data 1 and 2). Moreover, some proteins that have been reported to be associated with other NLR immune receptors or to take part in the immune signaling were also identified in our N PL. These proteins include the *Nb*TPL4^39^, *Nb*Nup88^56^, *Nb*MAP65^57^, *Nb*Asp^40^, and *Nb*MYC2^41^ (Supplementary Data 1 and 2; Supplementary Table 1). VIGS-based genetic screening further confirmed the functional role of *Nb*TPL4, *Nb*Asp, and *Nb*MYC2 in *N*-mediated resistance to TMV (Fig. 3). These results, on one hand, revealed the robustness of PL in probing the proteins associated with the N NLR or the components that constitute the *N*-mediated immune signaling network. On the other hand, these results suggest the evolutionary convergence of some signaling components that participate in various NLR-mediated defense responses^4^. For example, the nuclear pore complex component MOS7/Nup88 is required for nuclear accumulation of the suppressor of npr1-1 constitutive 1 (SNC1) NLR and subsequent defense activation^56^. Similarly, presence of N in the nucleus and association with SPL6 transcription factor in the nucleus post-TMV recognition is important to activate defense against TMV^11,12^, implicating a possible role for Nup88 in nuclear accumulation of N.

The absence of some known interactors in the PL dataset was observed when performing PL in mammalian cells^15^. Similarly, the NRIP1 and the SPL6, which associate with N in the previous studies^10,12^, were not identified in our PL dataset. Several factors may lead to the failure of the TurboID-based PL of these proteins such as steric hindrance or conformation masking of the primary amines for biotinylation in these known interactors, or the distance between N and these proteins beyond the labeling radius of the TurboID. Furthermore, N interaction with NRIP1 and SPL6 is known to occur at a specific window of time after TMV infection or p50 expression^10,12^, and hence performing PL at different time points after p50 expression could uncover these interactions. NRG1 NLR and EDS1 have been shown to be involved in *N*-mediated defense^42,58,59^, although physical association between these proteins has not been observed^59^. Significantly, the Hsp90 family protein that is known to associate with N^13^ is present in our MS dataset. These findings indicate that although TurboID-based interactome screening may not identify all partners of a target protein, it is able to identify some known and many previously unknown interactors of N.

Together, the datasets described in this work provide an overview of N associated signaling partners and enhance our understanding of the dynamic and intricate regulatory network during *N*-mediated defense response. Also, these results highlight the efficacy of using TurboID-based PL in identifying PPIs of NLR immune receptors.

Interestingly, our N interaction analyses identified several putative NLR proteins such as *Nb*R1B-14, *Nb*R1A-3, *Nb*RPP13L, *Nb*RGA1, and *Nb*RGA3. These findings are interesting considering recent emerging model indicating that NLRs may work in pairs. In this model, the sensor NLR recognizes the pathogen and the helper NLR is required for the function of a sensor NLR^35–37^. Since to date, NRG1 CC-NLR^59^ is the only NLR known to function downstream of N TIR-NLR, we evaluated the function of the NLR proteins identified from our PL experiments in *N*-mediated immunity. VIGS-based reverse genetic screening revealed that two putative NLRs, *Nb*RGA3 and *Nb*RPP13L, are required for *N*-mediated defense against TMV. In contrast, knock-down of the NLR NRC2a, which was not significantly enriched in our N PL dataset and was included as a negative control, did not affect *N*-mediated defense. These results suggest that NRC2a, which contributes to the immunity mediated by various sensor NLRs including Rx, Bs2, R8, and Sw5^36^, is not required for N NLR-mediated immunity. Our genetic screening revealed that only two NLRs (*Nb*RGA3 and *Nb*RPP13L) from p50-treated samples are required for the *N*-mediated defense. Lack of phenotype for the other NLRs could be that these NLRs may play a negative regulatory role in *N*-mediated defense. Thus, future studies should explore this possibility. In fact, functional roles of paired NLRs is complex. For example, the PigmS NLR suppresses PigmR NLR-mediated resistance by competitively attenuating PigmR homodimerization^60^ and RGA5 NLR inhibits RGA4 NLR-mediated cell death in the absence of pathogen infection^61^. In contrast, helper NLR, NRG1 plays a positive regulatory role in N NLR-mediated resistance against TMV^59^. These results further confirm the complex relationships in which different sensor NLRs require diverse helper NLRs to exert their functions. Our studies, for the first time, revealed that additional NLR proteins besides NRG1 are required for the *N*-mediated immune response. Further studies with these NLRs should provide insights into the NLR network functions during plant immune signaling.

Our N PL proteomic dataset, in the absence of p50 effector treatment, identified a mammalian homolog of a putative E3 ubiquitin ligase UBR7. UBR7 belongs to the UBR-box protein family that is highly conserved across mammals, flies, plants, and yeast^45,46^. To-date, UBR box protein family members have been shown to function in the N-end rule pathway, which has been shown to be part of the ubiquitin**-**proteasome system and regulate homeostasis of various physiological processes^46,62^. Seven different UBR box proteins, named UBR1 to UBR7, have been identified in mammals^45^^,^ However, only UBR1, UBR2, UBR4, and UBR5 have been shown to interact with the N-terminal degradation signals (N-degrons) and are classified as N-recognins, whereas UBR3, UBR6, and UBR7 do not recognize the canonical N-degron sequence^63^. In plants, only PRT1 and PRT6 (UBR1 homologs) have been identified as N-recognins and found to be involved in the N-end rule pathway^64,65^. Little information is available for other members of the UBR box proteins in plants^66^. Analysis of the *Arabidopsis* UBR7 homolog sequence revealed that it lacks several of the key residues required for N-degron recognition^67^. However, human UBR4 also lacks several of the key residues yet is still able to bind N-degrons^63^. Similarly, PRT1, the first N-recognin identified in plants, does not contain a UBR domain^65^.

Interestingly, *Nb*UBR7 was not identified in the N PL dataset generated in the presence of TMV p50 (Supplementary Data 2 and Supplementary Table 1), suggesting that p50 interferes with the interaction between N and UBR7 during *N*-mediated defense. Consistent with the proteomics data, our cellular, molecular and biochemical experiments demonstrated that the p50 effector interferes with the interaction between UBR7 and N. Interestingly, overexpression of NbUBR7 inhibited the cell death elicited by p50 in N plants (Fig. 5d), suggesting that the amount of p50 in the co-infiltrated leaves was insufficient to completely block the UBR7-N interaction and that UBR7 plays a dominant role under such conditions. This observation was further supported by in vivo BiFC assays in which the reconstituted fluorescence intensity generated by UBR7-N interaction was greatly reduced when the p50 was also expressed in the leaves (Fig. 6c). Furthermore, in vitro GST-pull down data revealed that p50 perturbs NbUBR7-N interaction in a dose-dependent manner (Fig. 6d). Taken together, these results suggest that the dynamic interaction occurs among N, p50, and UBR7 during the activation of plant immunity mediated by the N protein.

**Fig. 7:**
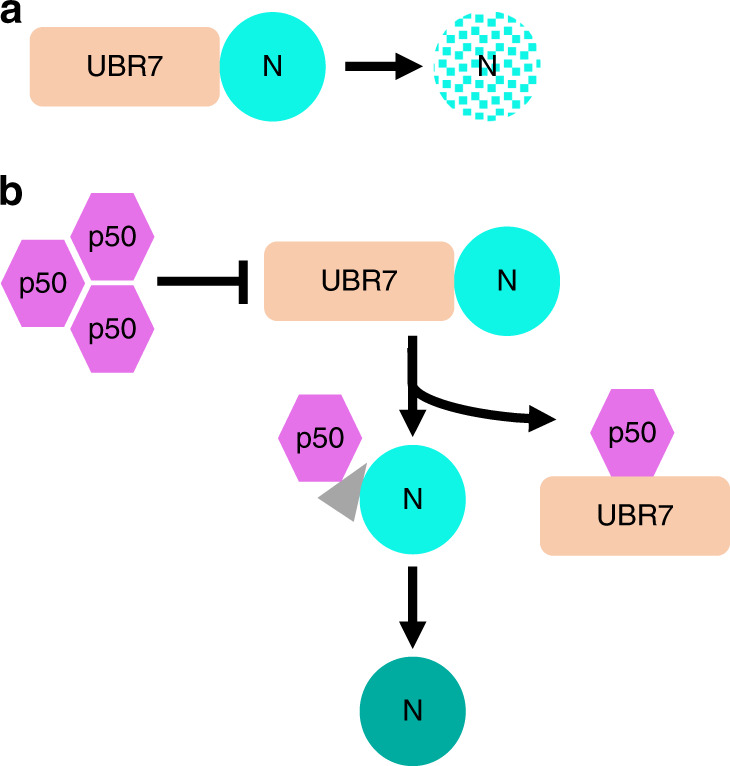
A model for the functional role of UBR7 in *N*-mediated resistance to TMV. **a** In uninfected naive condition, UBR7 interacts with the TIR domain of N resulting in the relatively low expression of N. **b** During TMV infection, the p50 effector disrupts the interaction between UBR7 and N by interacting with UBR7, thereby releasing the N from the N-UBR7 complex and leading to increased stability of N and activation of defense

Based on our findings, we propose a model for the functional role of UBR7 in the *N*-mediated immune signaling (Fig. 7). Under normal conditions, UBR7 interacts with N and reduces N stability, thereby maintaining a low level of N protein. When N containing plants encounter TMV, the p50 effector interacts with UBR7 and disrupts the interaction between N and UBR7. The disrupted interaction between N and UBR7 results in enhanced stability of N and activation of defense signaling. Further characterization of UBR7 will be important to understand how UBR7 regulates N stability and possible other roles of UBR7 in plant and mammalian systems.

In summary, we established the TurboID-based PL system in plants as a powerful proteomic tool for identification of the interaction partners of a target protein. Using the TurboID-based PL, a number of new regulators involved in the N NLR-mediated immune response were identified. In particular, we found that UBR7 interacts with N and functions as a negative regulator of *N*-mediated defense. Our results highlight the robustness of the TurboID-based PL in probing the interactome of NLR immune receptors and also enhance our understanding of the sophisticated regulatory network underpinning plant innate immunity.

## Methods

### Plant growth conditions

*N. benthamiana* plants were grown in a climate-controlled chamber with 16 h light and 8 h photoperiod at 22 °C.

### Construction of plasmids

The primers used for the construction of plasmids described below are listed in Supplementary Table 2 and DNA sequencing was performed to confirm all the plasmids. BioID (Addgene #53581) and citrine fragments were amplified by PCR and the resulting fragments were digested with *Stu*I/*Xma*I and *Avr*II/*Xma*I respectively and ligated into *Stu*I/*Avr*II digested pUBQ::MCS::3xHA vector to generate the plasmid pUBQ:Citrine-BioID-3xHA. pUBQ:Citrine-BioID2-3xHA was constructed using similar strategy except that the BioID2 fragment was generated by PCR using the Addgene #80899 plasmid as a template. pUBQ:Citrine-TurboID plasmid was constructed by replacing the BioID2 in the plasmid pUBQ:Citrine-BioID2-3xHA with PCR amplified TurboID fragment using the *Xma*I/*Avr*II restriction sites. pUBQ:BioID-3xHA:NOSt was generated by cloning PCR amplified BioID fragment into pUBQ::MCS::3xHA vector.

For the construction of p35S::gN-BioID, the PCR amplified BioID fragment was cloned into *Avr*II-digested p35S::gN-3HA plasmid. Similarly, the PCR amplified BioID2 and TurboID fragments were cloned into *Avr*II-digested p35S::gN-3HA plasmid respectively to generate the p35S::gN-BioID2 and p35S::gN-TurboID. For p35S::TIR-TurboID, the PCR amplified TIR and TurboID fragments were digested with *Kpn*I/*Xma*I and *Xma*I/*Avr*II respectively and ligated into *Kpn*I/*Avr*II digested p35S::gN-3HA plasmid. p35S::Citrine-TurboID was constructed using the similar strategy to that of p35S::TIR-TurboID.

To generate various VIGS constructs, 300 bp region of each gene was selected based on the SGN VIGS Tool (http://vigs.solgenomics.net) and the synthesized fragments (GENEWIZ) were cloned into TRV-RNA2 vector pYL156^42^.

To clone the full-length cDNA or genomic region containing exons and introns of several candidate genes for BiFC assay, reverse transcription (RT) was performed using the oligo d(T)primer to obtain the cDNA (see the RT-qPCR methods below for more details) or purified *N. benthamiana* genomic DNA was used as the template for PCR amplification of the target genes. The resulting fragments were recombined into pDONR207 vector (Invitrogen) by BP recombination followed by LR recombination into destination vectors with 3xHA-YC, resulting various YC-fused candidate genes.

To construct *Nb*UBR7-related constructs, the full-length *NbUBR7* cDNA was synthesized (GENEWIZ) and cloned into pDONR207 (Invitrogen) by BP recombination. The entry vector containing the *Nb*UBR7 was recombined into destination vectors with citrine, Y^C^, 3xHA or 3xMYC by LR recombination resulting in the plasmids *Nb*UBR7-citrine, *Nb*UBR7^YC^, *Nb*UBR7-3xHA, *Nb*UBR7-3xMYC. To generate *Nb*UBR7-RNAi vector, 638-937 nucleotide region from *Nb*UBR7 cDNA was amplified and recombined into pDONR207 vector and the resulting entry clone was recombined into pHELLSGATE 8^68^. To generate XVE::tRFP-p50-U1, the p50 region in the TMV clone was amplified and cloned into XVE vector^27^ digested with *Xho*I/*Apa*I.

For protein expression experiments, coding region of *Nb*UBR7 was PCR amplified and cloned into a GST-tag vector (Addgene #29707) linearized with *Ssp1*. N-TIR and RBOHD C-terminus region were cloned into His-GFP vector (Addgene #29663). p50 was cloned into pTYB2 vector (NEB, Catalog number N6702).

Other plasmids used in this study include gN^YN^, p50U1^YC^, N∆TIR^YN^, N-TIR^YN^, p50U1^YN^, GUS^YC^ have been described in previous studies^10–12^.

### Agroinfiltration

*Agrobacterium tumefaciens* strain GV3101 harboring different expression constructs were infiltrated into *N. benthamiana* leaves as described previously^10,12^. *Agrobacterium* cultures containing expression vectors for N or its truncation mutants were adjusted to OD_600_ = 2.1; other constructs to OD_600_ = 1.0. For coinfiltration assays, different cultures were mixed in a 1:1 or 1:1:1 proportion and infiltrated into four-week-old *N. benthamiana* leaves. During PL, 36 h after agroinfiltration, 200 µM biotin was infiltrated into the same leaf sectors. For the tRFP-p50 expression, 30 µM 17-β-estradiol were infiltrated into the *N. benthamiana* leaves to induce its expression. For -p50 treatment also 30 µM 17-β-estradiol was infiltrated to eliminate any effects from 17-β-estradiol treatment.

### TurboID sample preparation for MS analysis

For each TurboID-fused construct (gN-TurboID-3xHA, N-TIR-TurboID-3xHA, and Citrine-TurboID-3xHA), three biological replicates were performed and analyzed via MS. Specifically, leaves of four-week old *N. benthamiana* plants were infiltrated with agrobacteria harboring a TurboID construct. 36 h later, infiltration solution containing 200 µM biotin and 10 mM MgCl_2_ was infiltrated into the same leaf tissues. Four infiltrated leaves from each plant were then harvested after 12 h incubation and frozen in liquid nitrogen. Frozen leaf material was ground to a fine powder in a mortar and protein extraction was performed by addition of 1 mL RIPA lysis buffer [50 mM Tris-HCl (pH 7.5), 500 mM NaCl, 1 mM EDTA, 1% NP40 (v/v), 0.1% SDS (w/v), 0.5% sodium deoxycholate (w/v), 1 mM DTT, 1 tablet of cOmplete™ Protease Inhibitor Cocktail (Roche, Catalog number 11697498001] to 700 mg leaf powder. After vortex-mixing, the tubes were immediately centrifuged at 16,500 *g* for 10 min, upper soluble fraction was then run through the Zeba™ Spin Desalting Column (Thermo Fisher Scientific, Catalog number 89893) to remove the excess biotin in lysates. 100 µL lysate was then taken out from the desalted extracts and used to estimate the protein concentration by using the Pierce™ BCA Protein Assay Kit (Thermo Fisher Scientific, Catalog number 23225).

To enrich biotinylated proteins from the protein extracts, 200 µL of streptavidin-coated magnetic beads (Dynabeads™ MyOne™ Streptavidin C1, Invitrogen, Catalog number 65001) were washed twice with RIPA lysis buffer, the desalted lysates containing ~6 mg protein were then incubated with the equilibrated beads on a rotator overnight at 4 °C. The beads were sequentially washed once with 1 mL buffer I (2% SDS in water), once with buffer 2 [50 mM HEPES (pH 7.5), 500 mM NaCl, 1 mM EDTA, 0.1% deoxycholic acid (w/v), 1% Triton X-100], and once with buffer 3 [10 mM Tris-HCl, pH 7.4, 250 mM LiCl, 1 mM EDTA, 0.1% deoxycholic acid (w/v), 1% NP40 (v/v)]. To completely remove the potential detergent, the beads were washed twice in 50 mM Tris-HCl, pH 7.5 and six more times in 50 mM ammonium bicarbonate, pH 8.0. Finally, the beads were resuspended in 1 mL 50 mM ammonium bicarbonate. To confirm the successful enrichment of the biotinylated proteins, ten percent of the suspension was taken out for Western blot analysis and the rest of the beads were flash-frozen in liquid nitrogen and stored at −80 °C or sent immediately on the dry ice for LC-MS/MS analysis.

### On-bead trypsin digestion of biotinylated proteins

Biotinylated proteins enriched with streptavidin beads were processed into peptides via on bead digestion and analyzed by LC-MS/MS according to the previously described methods^20,30,32^. The samples were incubated for 15 min at 65 °C in 50 mM ammonium bicarbonate with Tris (2-carboxyethyl) phosphine hydrochloride (TCEP-HCl) to a final concentration of 1 mM. Proteins were then digested with 0.5 µg trypsin (Roche, Cat. No. 03708969001) and 0.1 µg LysC (Wako Chemicals, Catalog number 125-05061) overnight at 37 °C. A second digestion was then carried out using 0.5 µg trypsin and 0.05 µg LysC for 3 h at 37 °C. Iodoacetamide (IAM) was added to the digested peptides to a final concentration of 2.5 mM and the samples were incubated at 37 °C for 30 min in the dark. Peptides were separated from the streptavidin beads using a magnet and acidified by adding formic acid to a pH of ~2–3. Peptides were then desalted with 50 mg Sep-Pak C18 cartridges (Waters). Eluted peptides were dried using a SpeedVac and resuspended in water. Peptide amount was then estimated using the Pierce Quantitative Colorimetric Assay (Thermo Fisher Scientific, Catalog number 23275).

### TMT labeling

An equal volume of each TurboID sample was used for labeling with TMT10plex™ isobaric label reagents (Thermo Fisher Scientific, Catalog number 90110)^31^. To achieve a labeling efficiency of >99%, the labeling reactions were performed as follows: equal volume aliquots of recovered peptides were dried using a SpeedVac and resuspended in 25 µL of 200 mM HEPES (pH 8.5). To resuspend the TMT labeling reagent 41 µL of acetonitrile was added to each tube of TMT (0.8 mg), vortexed, and incubated at RT for 5 min. Ten µL of TMT labeling solution was then added to each tube of peptides, pipetted up and down for several times, and vortexed to mix. After 2 h incubation at RT, 2 µL of 5% hydroxylamine was added to each tube, vortexed, and incubated at RT for 15 min to quench the reaction of labeling. Following quenching, an equal volume from each TMT labeling reaction was pooled for analysis by 2D-LC-MS/MS.

### Liquid chromatography

An Agilent 1260 quaternary HPLC was used to deliver a flow rate of ~600 nL/min via a splitter. All columns were packed in house using a Next Advance pressure cell and the nanospray tips were fabricated using fused silica capillary that was pulled to a sharp tip via a laser puller (Sutter P-2000). 15 µg of pooled TMT labeled peptides were loaded onto a 10 cm capillary column packed with 5 µM Zorbax SB-C18 (Agilent), which was connected using a zero dead volume 1 µm filter (Upchurch, M548) to a 5 cm long strong cation exchange (SCX) column packed with 5 µm PolySulfoethyl (PolyLC). The SCX column was then connected to a 20 cm nanospray tip packed with 2.5 µM C18 (Waters). The 3s were joined and mounted on a Nanospray Flex Ion Source (Thermo Scientific) for on-line nested peptide elution. A new set of columns was used for each sample (Group I and Group II). Peptides were eluted from the loading column onto the SCX column using a 0–80% acetonitrile (ACN) gradient over 60 min. Peptides were then fractionated online from the SCX column using the following series of ammonium acetate salt steps: 25, 40, 50, 60, 70, 90, 100, 130, and 1000 mM. For these analyses, buffers A (99.9% H_2_O, 0.1% formic acid), B (99.9% ACN, 0.1% formic acid, C (100 mM ammonium acetate, 2% formic acid), and D (2 M ammonium acetate, 2% formic acid) were utilized. For each salt step, a 150 min gradient program comprised of a 0–5 min increase to the specified ammonium acetate concentration, 5–10 min hold, 10–14 min at 100% buffer A, 15–120 min 10–35% buffer B, 120–140 min 35–80% buffer B, 140–145 min 80% buffer B, and 145–150 min buffer A was employed.

### Mass spectrometry

The samples were analyzed using a Thermo Scientific Q-Exactive Plus high-resolution quadrupole-Orbitrap mass spectrometer. Data-dependent acquisition was obtained using Xcalibur 4.0 software in positive ion mode with a spray voltage of 2.00 kV and a capillary temperature of 275 °C and an RF of 60. MS1 spectra were measured at a resolution of 70,000, an automatic gain control (AGC) of 3e6 with a maximum ion time of 100 ms, and a mass range of 400–2000 m/z. Up to 15 MS2 with a fixed first mass of 100 were triggered at a resolution of 35,000. An AGC of 1e5 with a maximum ion time of 50 ms, an isolation window of 1.5 m/z, and a normalized collision energy of 32 were used. Charge exclusion was set to unassigned, 1, 5–8, and >8. MS1 that triggered MS2 scans were dynamically excluded for 25 s.

### MS data analysis

The raw data were analyzed using MaxQuant version 1.6.1.0^33^. Spectra were searched, using the Andromeda search engine^69^, against either the *N*. *benthamiana* sequence v1.0.1 proteome file entitled “Niben101_annotation.proteins.wdesc.fasta” that was downloaded from the SOL Genomics website (ftp://ftp.solgenomics.net/genomes/Nicotiana_benthamiana) or the *Nicotiana tabacum* (UP000084051) downloaded from Uniprot. The proteome files were complemented with reverse decoy sequences and common contaminants by MaxQuant. Carbamidomethyl cysteine was set as a fixed modification while methionine oxidation and protein N-terminal acetylation were set as variable modifications. The sample type was set to “Reporter Ion MS2” with “TMT10plex” selected for both lysine and N-termini. TMT batch-specific correction factors were configured in the MaxQuant modifications tab (TMT Lot No. TB260979). Digestion parameters were set to “specific” and “Trypsin/P;LysC”. Up to two missed cleavages were allowed. A false discovery rate, calculated in MaxQuant using a target-decoy strategy^70^, less than 0.01 at both the peptide spectral match and protein identification level was required. The match between runs feature of MaxQuant was not utilized.

To normalize the data, TMT intensity values were median centered so that the total TMT intensity for each sample was equal. Statistical analyses and hierarchical clustering of the proteomics data was performed using Perseus^34^. No imputation for missing values was performed. Statistical analyses to uncover enriched interacting proteins was done via two-sample *t*-tests coupled with permutation-based false discovery rate (FDR) correction. Proteins were called as significantly enriched interactors if they had a *q*-value ≤ 0.05 and a fold enrichment >1.5 (N/citrine or TIR/citrine). Hierarchical clustering was performed using Euclidian distance and Complete linkage on the significantly enriched proteins following z-score row normalization. The clustered enriched proteins were then visualized as a Heat Map in Perseus. Finally, the unions and intersections of the different enriched proteins sets were visualized as Venn diagrams drawn using the Pangloss Venn Diagram generator (http://www.pangloss.com/seidel/Protocols/venn.cgi).

### VIGS assay

VIGS assays were performed using our previously described method in transgenic *N*-containing *N. benthamiana* plants^42^. When TRV-PDS infiltrated plants showed uniform photobleaching phenotype in the upper leaves (~12 days post infiltration), upper leaves from various VIGS construct-inoculated plants were mechanically inoculated with TMV-U1 virus sap or TMV-U1-GFP virus sap. The plants were monitored for the development of HR cell death and systemic infection up to 14 days post-TMV infection. GFP fluorescence in inoculated leaf (IL) and upper uninoculated leaves was observed under UV light. VIGS assay was repeated three times using up to a total of 5 plants per VIGS construct.

### RT-qPCR and RT-PCR

RT-qPCR analysis was performed according to a previous report with minor modifications^42^. Total RNA from VIGS plants was extracted using TRIzol reagent (Invitrogen). First strand cDNA was prepared from 1 µg total RNA using the oligo d(T)primer and SuperScript II reverse transcriptase (Invitrogen). qPCR was performed using iTaq Universal SYBR Green Supermix (Bio-Rad) in the CFX96 Touch Real-Time PCR Detection System (Bio-Rad). *eIF4A* or *PP2A* was used as the internal control to normalize the data. The fold change in mRNA levels was determined using the ΔΔCt method. For RT-PCR analysis of the TMV-U1 infection, primer SP6289 was used for the synthesis of first strand cDNA followed by PCR amplification of the movement protein region of TMV-U1. The primers used for RT-qPCR and RT-PCR analysis are listed in Supplementary Table 3.

### Western blot analysis

Proteins were separated on 8% in-house prepared SDS-PAGE gels. In the case of Hsp90 experiments, proteins were separated on 4–15% gradient Mini-PROTEAN TGX Precast Gels (Bio-Rad, Catalog number 4561084). Western blot analysis of the protein extracts was performed according to the procedures described previously^10,12^. Briefly, plant tissue expressing the protein(s) of interest was collected and ground in liquid nitrogen. Total protein extracts were prepared followed by SDS–PAGE, proteins were then transferred to Immobilon-P PVDF membrane (Millipore, Catalog number IPVH00010) using the Trans-Blot Turbo Transfer System (Bio-Rad). Membranes were blocked for 1.5 h in 5% fat-free milk in PBST (for immunoblot analysis) or 2.5% BSA in PBST (for streptavidin-HRP) and then incubated with the appropriate primary antibodies or streptavidin-HRP, followed by incubation with corresponding secondary antibodies. Antibodies used for western blot include mouse anti-c-MYC (Santa Cruz, Catalog number sc-40; 1:1000 dilution), rabbit anti-tRFP (Evrogen, Catalog number AB233; 1:1000 dilution), rabbit anti-t[CGY]FP (Evrogen, Catalog number EVN-AB121; 1:1000 dilution), rat anti-HA (Roche, Catalog number 11867423001; 1:1000 dilution), rabbit anti-PEPC (Abcam, Catalog number ab34793; 1:1000 dilution), Streptavidin-HRP (Abcam, Catalog number ab7403; 1:20,000 dilution), anti-HA-HRP (Roche, Catalog number 12013819001; 1:1000 dilution), anti-MYC-HRP (Sigma-Aldrich, Catalog number 16-213; 1:1000 dilution), and anti-mouse (Sigma-Aldrich, Catalog number A4416; 1:5000 dilution), anti-rat (Santa Cruz, Catalog number sc-2065; 1:5000 dilution) or anti-rabbit peroxidase (Sigma-Aldrich, Catalog number A0545; 1:5000 dilution). Bands were visualized by using SuperSignal™ West Pico PLUS Chemiluminescent Substrate (ThermoFisher Catalog number 34577) or the Bio-Rad Clarity™ Western ECL Substrate (BioRad, Catalog number 1705060) according to the manufacturer’s instructions. Chemiluminescent signals were acquired by using a ChemiDoc™ Touch Imaging System (Bio-Rad). Quantification of the band signal intensity was performed with the ImageJ software.

### Confocal microscopy

Live plant tissue imaging was performed on a Zeiss LSM710 confocal microscope (Carl Zeiss) equipped with Zeiss Zen software. Tissue samples were cut from *N. benthamiana* leaves at approximately 46 hpi. Citrine and RFP fluorescence was visualized under 514 nm and 543 nm, respectively with an argon laser. Images within a panel were taken using the same confocal settings and processed together with Imaris 7.4.2 software (Bitplane).

### Immunoprecipitation assay

Immunoprecipitation assays with Hsp90 or co-immunoprecipitation of NbUBR7 and p50 was performed according to previously described method^10,12^. Briefly, *N. benthamiana* leaves agroinfiltrated with different expression vectors were harvested and ground in liquid nitrogen as described above. Leaf powders were transferred to a 1.5 mL centrifuge tube and mixed with 3 volumes of co-immunoprecipitation buffer [100 mM NaCl, 20 mM Tris (pH 7.5), 1 mM EDTA (pH 8.0), 0.1% Triton X-100, 10% glycerol, 5 mM DTT, 2 mM NaF, 1 mM PMSF] and cOmplete™ Protease Inhibitor Cocktail (Roche). The extracts were centrifuged and upper supernatant was pre-cleared with protein G sepharose beads (Amersham Bioscience). The resultant supernatants were incubated with anti-c-MYC Agarose Affinity Gel antibody (Sigma-Aldrich, Catalog number A7470) or monoclonal Anti-HA−Agarose antibody produced in mouse (Sigma-Aldrich, Catalog number A2095) at 4 °C for 4 h on a rotating wheel. The beads were washed 3 times with co-immunoprecipitation buffer containing 200 mM NaCl and boiled with 2 X loading buffer (Invitrogen). Samples were separated on an SDS-PAGE gel followed by western blot analysis.

### Purification of recombinant proteins and in vitro pull down

Recombinant proteins from *E. coli* were purified as previously described with some modifications^71^. BL21 strain of *E. coli* was used for protein expression. 1 L of 2 X YT media (RPI Corp) was used for GST-NbUBR7, His-GFP-N-TIR, His-GFP-RBOHD-C, and pTYB2-p50 expression. Cells were harvested using centrifugation and resuspended in lysis buffer [30 mM Tris (pH 7.5) and 120 mM NaCl]. Cells were lysed using microfluidizer and lysates were incubated with 2 mL of GST beads for GST-NbUBR7, 2 mL of cobalt beads for His-GFP-N-TIR or His-GSP-RBOHD-C and 2 mL of chitin beads for pTYB2-p50 for 3 h at 4 °C. Proteins were eluted and further purified using size exclusion chromatography with HiLoad 16/600 Superdex column equilibrated with 30 mM Tris (pH 7.5) and 120 mM NaCl.

In vitro pull-down assay was performed by incubating 1 µg of GST-NbUBR7 with 3 µg of His-GFP-N-TIR or His-GFP-RBOHD-C protein. For competition assay, 1 µg of GST-NbUBR7 was pre-incubated with increasing quantities of p50 protein on ice for 1 h before adding 1 µg of His-GFP-N-TIR and further incubated for 2 h at 4 °C with moderate shaking. Pull-down proteins with GST beads was probed with anti-GFP antibody to detect bound protein.

### Reporting summary

Further information on research design is available in the Nature Research Reporting Summary linked to this article.

## Supplementary information

Supplementary Information

Description of Additional Supplementary Files

Supplementary Data 1

Supplementary Data 2

Reporting Summary

## Data Availability

Source data for Fig. 3 are provided in the paper in Supplementary Data 1 and 2. The original MS proteomics raw data, as well as the MaxQuant output files, may be downloaded from MassIVE (http://massive.ucsd.edu) using the identifier: MSV000083018 and MSV000083019. The source data underlying Figs. 1b, c, d, 3b, d, 4a, c, 5a, b, c, d, 6b, d and Supplementary Figs. 1a, b, c, d, 4b, 5c, d, 6b, 7, 9b and 13 are provided as a Source Data File.

## Source data

Source Data
