# Author Correction: TurboID-based proximity labeling reveals that UBR7 is a regulator of N NLR immune receptor-mediated immunity

**DOI:** 10.1038/s41467-021-26441-2

**Published:** 2021-10-21

**Authors:** Yongliang Zhang, Gaoyuan Song, Neeraj K. Lal, Ugrappa Nagalakshmi, Yuanyuan Li, Wenjie Zheng, Pin-jui Huang, Tess C. Branon, Alice Y. Ting, Justin W. Walley, Savithramma P. Dinesh-Kumar

**Affiliations:** 1 Department of Plant Biology and The Genome Center, College of Biological Sciences, University of California, Davis, Davis, CA 95616 USA; 2 State Key Laboratory of Agro-Biotechnology and Ministry of Agriculture Key Laboratory of Soil Microbiology, College of Biological Sciences, China Agricultural University, 100193 Beijing, China; 3 Department of Plant Pathology and Microbiology, Iowa State University, Ames, IA 50011 USA; 4 Departments of Genetics, Stanford University, Stanford, CA 94305 USA; 5 Department of Chemistry, Stanford University, Stanford, CA 94305 USA; 6 Department of Biology, Stanford University, Stanford, CA 94305 USA; 7 Department of Chemistry, Massachusetts Institute of Technology, Cambridge, MA 02139 USA; 8 Chan Zuckerberg Biohub, San Francisco, CA 94158 USA

**Keywords:** Plant immunity, Proteomic analysis

Correction to: *Nature Communications* 10.1038/s41467-019-11202-z, published online 19 July 2019.

The original version of the Supplementary Information associated with this Article contained an error in Supplementary Fig. 8 in which panel c was inadvertently duplicated from panel b. This panel was a graphical summary of data presented in Supplementary Data 2 which was correct at the time of publication.

The HTML has been updated to include a corrected version of the Supplementary Information.

## Supplementary information

Updated Supplementary Information

