# Supplementary material for: TurboID-based proximity labeling reveals that UBR7 is a regulator of N NLR immune receptor-mediated immunity
Source: Nat Commun. 2019 Jul 19;10:3252. doi: 10.1038/s41467-019-11202-z (PMC6642208; doi:10.1038/s41467-019-11202-z)
Supplement: Supplementary file 1 — Supplementary Information [file 41467_2019_11202_MOESM1_ESM.pdf]

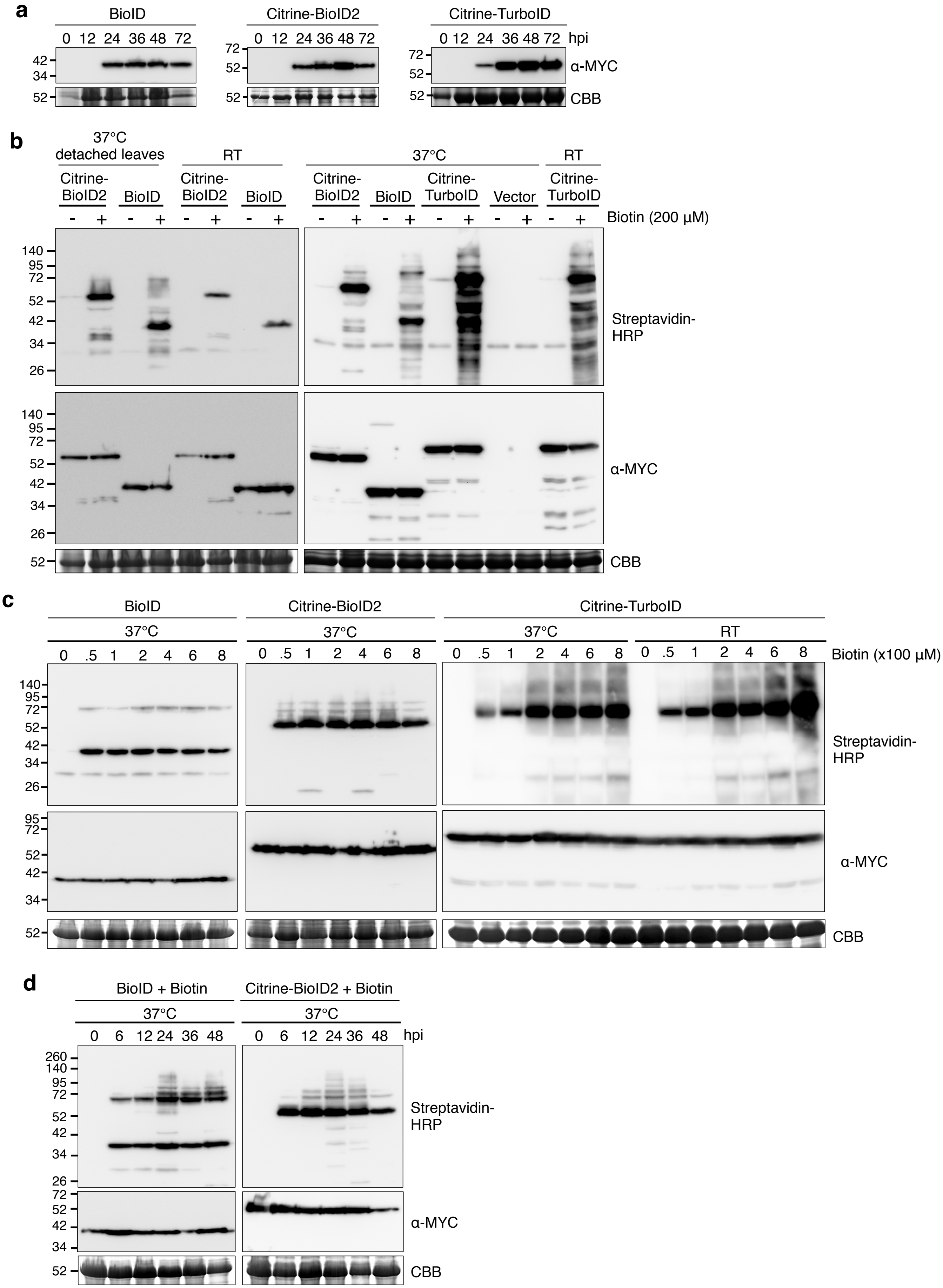
a
BioID
Citrine-BioID2
Citrine-TurboID
0 12 24 36 48 72 hpi
α-MYC
CBB
b
37°C detached leaves
RT
37°C
RT
Citrine-BioID2
BioID
Citrine-TurboID
Vector
Biotin (200 μM)
Streptavidin-HRP
α-MYC
CBB
c
BioID
Citrine-BioID2
Citrine-TurboID
37°C
RT
0 .5 1 2 4 6 8
Biotin (x100 μM)
Streptavidin-HRP
α-MYC
CBB
d
BioID + Biotin
Citrine-BioID2 + Biotin
37°C
0 6 12 24 36 48 hpi
Streptavidin-HRP
α-MYC
CBB

Supplementary Figure 1. Characterization of promiscuous protein biotinylation by BioID, BioID2 and TurboID in plants. (**a**) Time course analysis of protein expression in *N. benthamiana* leaves agroinfiltrated with the expression cassette of BioID, citrine-BioID2 or citrine-TurboID. Leaves were harvested at different time points (hpi) as indicated above the panels. Anti-MYC antibody was used for detection of different biotin ligases (top panels). Molecular size markers in kDa are indicated on the left. CBB-stained gel is shown as a loading control (bottom panels). (**b**) Comparison of the activity of three different biotin ligases under RT and 37°C. Different biotin ligases promiscuously biotinylate endogenous protein in plant cells with varying efficiencies. *N. benthamiana* leaves were agroinfiltrated with the agrobacterium containing BioID-3xMYC (BioID), citrine-BioID2-3xMYC (Citrine-BioID2) and citrine-TurboID-3xMYC (Citrine-TurboID), or the empty vector control (vector). 36 hours post-agroinfiltration (hpi), medium containing the buffer (-) or 200 μM biotin (+) were infiltrated into the previously agroinfiltrated leaves. Leaves were detached from some of the infiltrated *N. benthamiana* plants and incubated in a moist box under 37°C, other infiltrated *N. benthamiana* plants were directly incubated at room temperature (RT) or in a 37°C chamber. Western blot analysis was performed at 12 h after infiltration of biotin. Streptavidin-HRP and anti-MYC antibody were used for detection of biotinylated proteins (top panels) and different biotin ligases (middle panels), respectively. Coomassie Brilliant Blue (CBB)-stained gel is shown as a loading control (bottom panels). The molecular weight size markers in kDa are indicated at the left of each panel. (**c**) Determination of the optimal biotin concentrations used for BioID, BioID2 or TurboID-based proximity labeling in plant. 36 hours post agroinfiltration of BioID, BioID2 or TurboID constructs, different concentrations of biotin indicated above the panels were infiltrated into the same leaf sectors and the plants were incubated at 37°C. 12 hours post-biotin infiltration, tissue was harvested and Western blot analyses was performed using streptavidin-HRP to detect biotinylated proteins (top panels) and anti-MYC antibody to detect BioID, BioID2 and TurboID biotin ligases (middle panels). Coomassie Brilliant Blue (CBB)-stained gel is shown as a loading control (bottom panels). The molecular weight size markers in kDa are indicated at the left. **(d)** Determination of the optimal incubation time used for BioID or BioID2-based proximity labeling in plants. Post-biotin infiltration, tissue was collected at different time points as indicated above the panels and Western blot analyses was performed as described in supplementary Figure 1b. Source data are provided as a Source Data file.

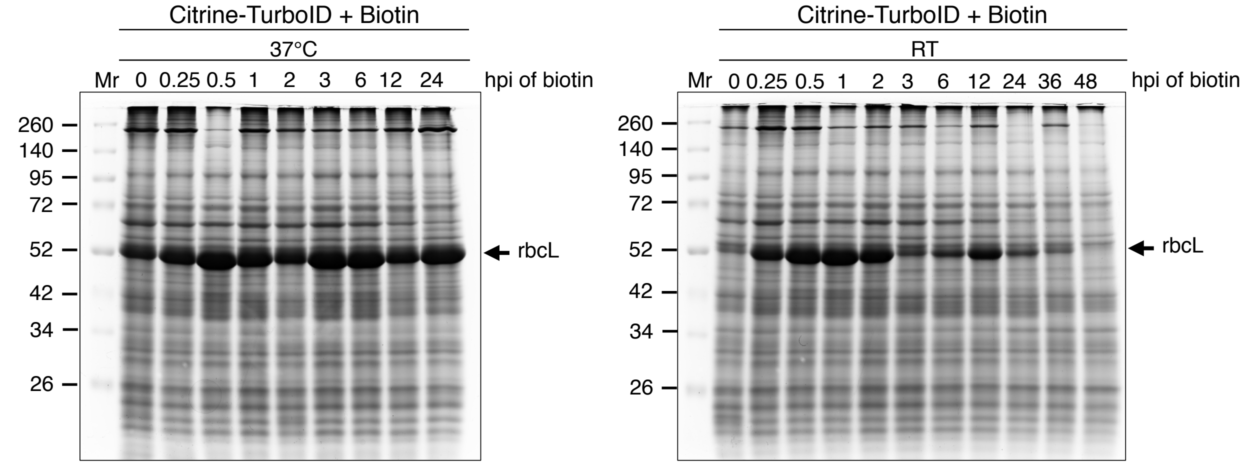

Supplementary Figure 2. Coomassie brilliant blue-stained gel images corresponding to that shown in Figure 1d. rbcL, large subunit of RuBisCO. Molecular size markers in kDa are indicated on the left.

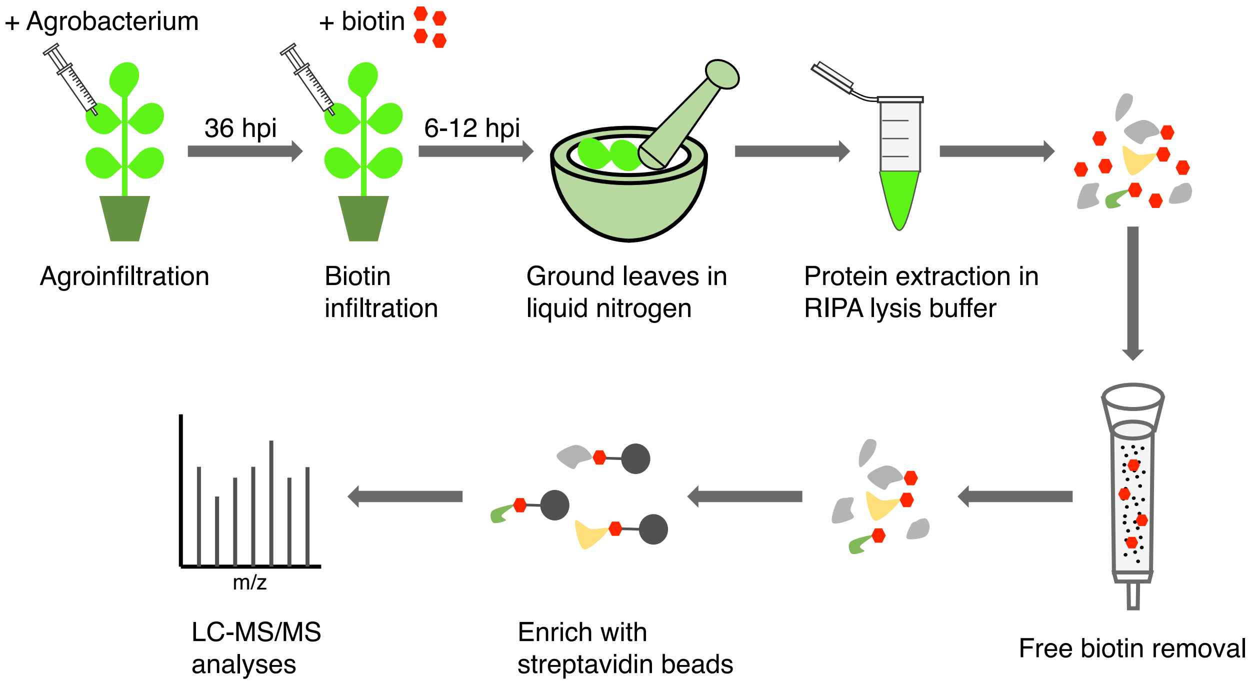

Supplementary Figure 3. Schematic representation of TurboID-based proximity labeling method in *N. benthamiana*. *N. benthamiana* leaves were agroinfiltrated with agrobacterium containing the TurboID-fusion constructs followed by addition of biotin to initiate the biotinylation of the endogenous proteins. After 6-12 hours incubation at RT, infiltrated leaves were ground in liquid nitrogen and cells were lysed in the RIPA lysis buffer. Free biotin was removed from the protein extracts by desalting and the biotinylated proteins were then enriched with streptavidin-conjugated beads. These candidate proteins can be identified by mass spectrometry.

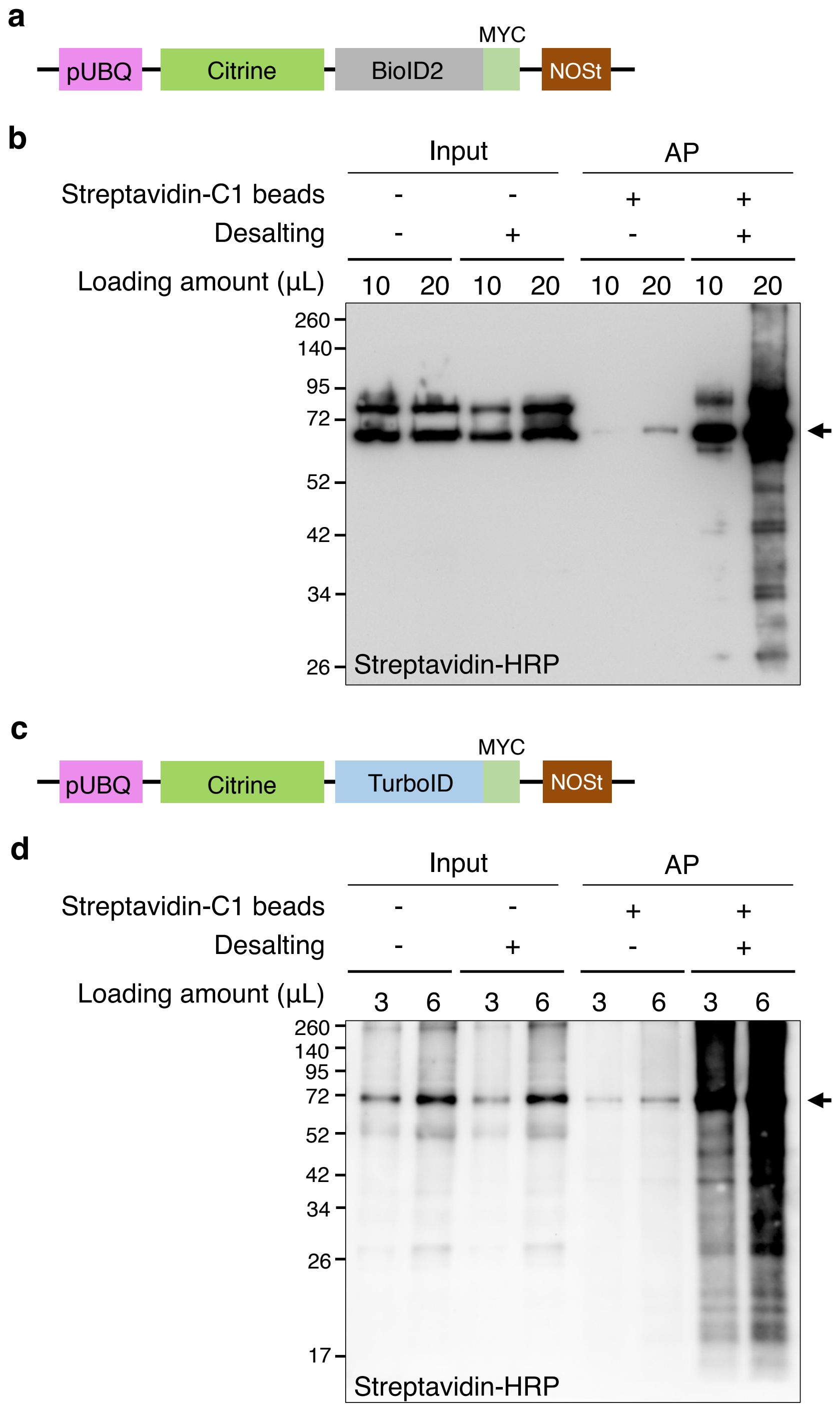

Supplementary Figure 4. Immunoblot analysis of the enriched biotinylated proteins with or without desalting treatment. Protein extracts from the leaves agroinfiltrated with the construct shown in (**a**) or (**c**) were subjected to desalting treatment followed by affinity purification using streptavidin-conjugated beads or directly affinity-purified without desalting. Different amounts of proteins (μL) prior to (input) or after the affinity purification (AP) were loaded onto the SDS-PAGE followed by Western blot analysis using HRP-conjugated streptavidin (**b** and **d**). pUBQ, Arabidopsis ubiquitin-10 promoter; NOSt, nopaline synthase terminator. "–" and "+" indicates without or with desalting treatment. The molecular weight size markers in kDa are indicated on the left of the panel. Arrow indicates the self-biotinylated protein bands of citrine-BioID2 (**b**) or citrine-TurboID (**d**). Source data are provided as a Source Data file.

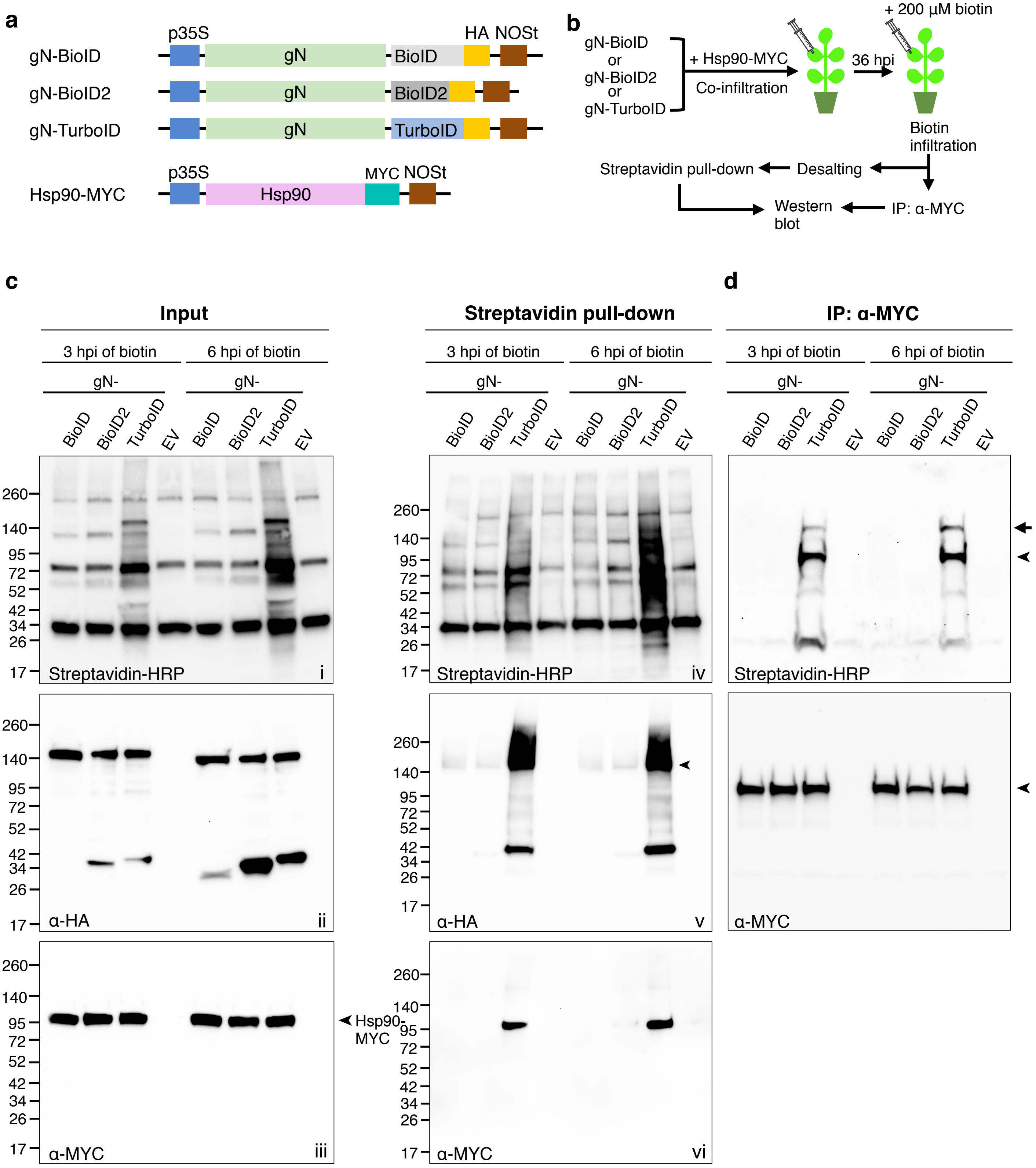
a
p35S
HA
NOSt
gN-BioID
gN
BioID
gN-BioID2
BioID2
gN-TurboID
TurboID
Hsp90-MYC
Hsp90
MYC
b
gN-BioID
or
gN-BioID2
or
gN-TurboID
+ Hsp90-MYC
Co-infiltration
36 hpi
+ 200 μM biotin
Biotin
infiltration
Desalting
Streptavidin pull-down
IP: α-MYC
Western
blot
c
Input
Streptavidin pull-down
3 hpi of biotin
6 hpi of biotin
gN-
BioID
BioID2
TurboID
EV
Streptavidin-HRP
α-HA
α-MYC
Hsp90-
MYC
i
ii
iii
iv
v
vi
d
IP: α-MYC
260
140
95
72
52
42
34
26
17

Supplementary Figure 5. TurboID performs better in promiscuously labeling a known interactor of N NLR immune receptor. (**a**) Schematic representation of the constructs used for comparison of proximity labeling activity of different biotin ligases. BioID-HA, BioID2-HA or TurboID-HA were fused with the N immune receptor, respectively. Hsp90 fused to MYC tag served as a known interactor of N NLR. (**b**) Experimental workflow for the analysis of Hsp90 biotinylation by BioID, BioID2 or TurboID-fused N. (**c**) Streptavidin pull-down analysis of the Hsp90 biotinylation by BioID-, BioID2- or TurboID-fused N. *Agrobacteria* containing empty vector (EV) infiltrated into the leaves served as a negative control. Panels i to iii: Western blot analysis of the total input proteins with Streptavidin-HRP (panel i), anti-HA (panel ii) and anti-MYC antibody (panel iii). Western blot analysis of the Streptavidin pull-down products from leaf samples collected 3 h and 6 h after biotin treatment probed with Streptavidin-HRP (panel iv), anti-HA (panel v) and anti-MYC antibody (panel vi). The molecular weight size markers in kDa are indicated at the left of each panel. Arrow head in panel v indicates the expected N-BioID, N-BioID2 or N-TurboID bands. (**d**) Immunoprecipitation analysis of the Hsp90 biotinylation by using anti c-MYC agarose beads. Western blot analysis of the IP products using Streptavidin-HRP (top panel) and anti-MYC antibody (bottom panel). Arrow and arrow head indicate the expected N-TurboID and Hsp90 bands, respectively. The molecular weight size markers in kDa are indicated at the left of each panel. Source data are provided as a Source Data file.

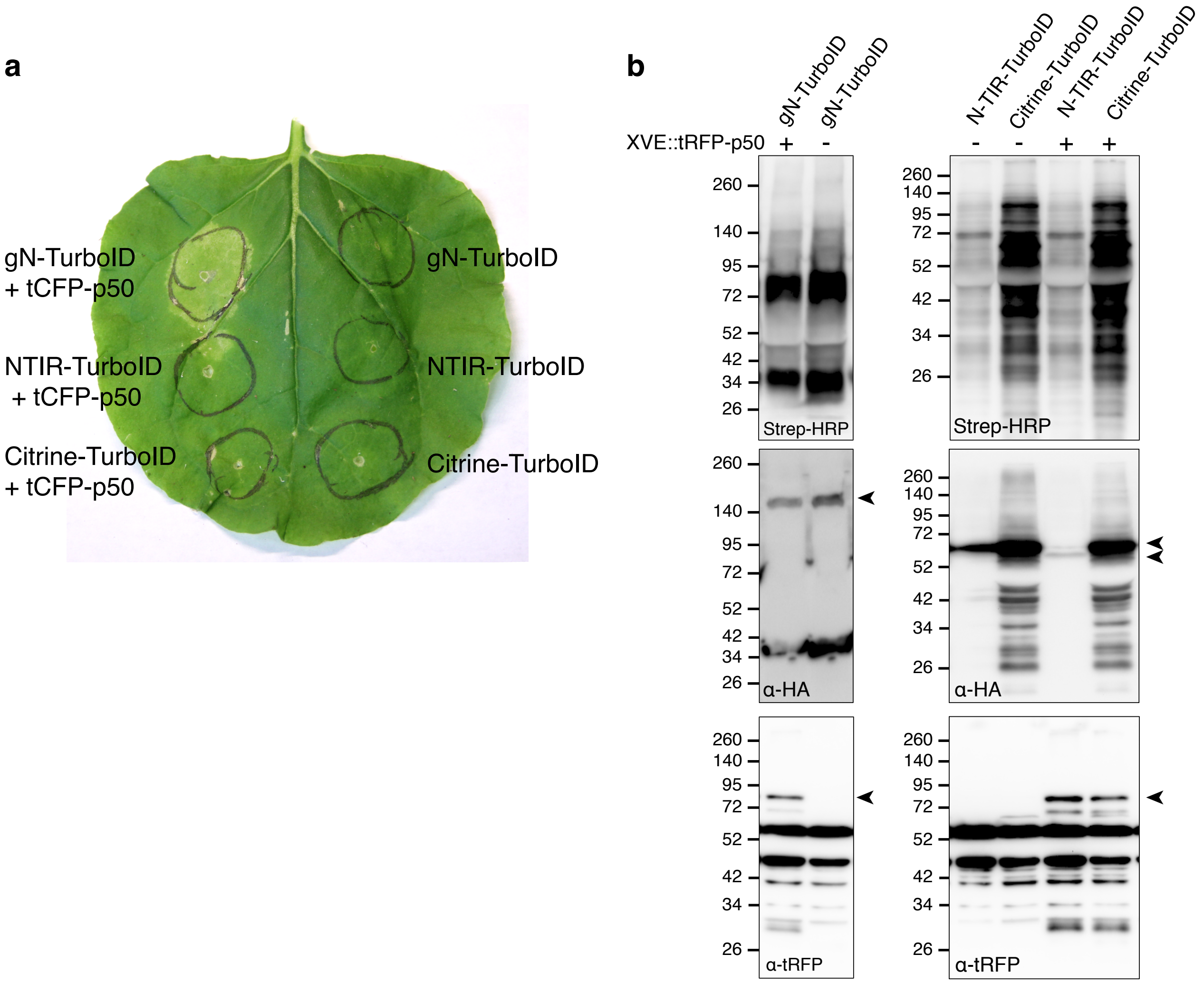

Supplementary Figure 6. Characterization of various TurboID-fusions used for identification of proximal and interacting proteins of N. (**a**) Analysis of the ability of various TurboID-fusions to induce HR-PCD in the presence or absence of p50 effector. Full length gN or TIR domain constructs were coinfiltrated with or without tCFP-p50. Photograph of the infiltrated leaf was taken 4 dpi. (**b**) Western blot analysis of the biotinylation and protein expression in the leaves agroinfiltrated with various TurboID-fusions. Streptavidin-HRP and anti-HA antibody were used for detection of biotinylated proteins (top panels) and different biotin ligases (middle panels) in the agroinfiltrated leaves, respectively. Estradiol-induced expression of TagRFP-fused p50 effector was assessed by Western blot analysis using anti-tagRFP antibody (bottom panels). "-" and "+" indicate the absence or presence of tagRFP-p50. Arrowheads indicate the specific band of the TurboID fusion protein. The molecular weight size markers in kDa are indicated on the left of each panel. Source data are provided as a Source Data file.

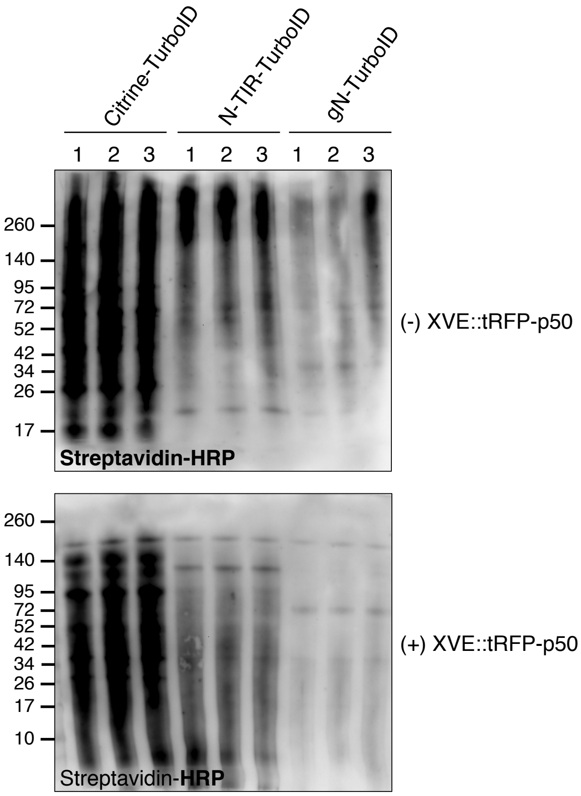

Supplementary Figure 7. Western blot analysis of the enriched protein samples used for LC-MS/MS analysis. Two groups of samples were prepared by agroinfiltration of various TurboID-fusions alone (without XVE:tRFP-p50) or together with the p50 effector (XVE::tRFP-p50). For each sample, there are three independent replicates (1, 2 and 3). Streptavidin-HRP was used for detection of biotinylated proteins in different samples. The molecular weight size markers in kDa are indicated on the left of each panel. Source data are provided as a Source Data file.

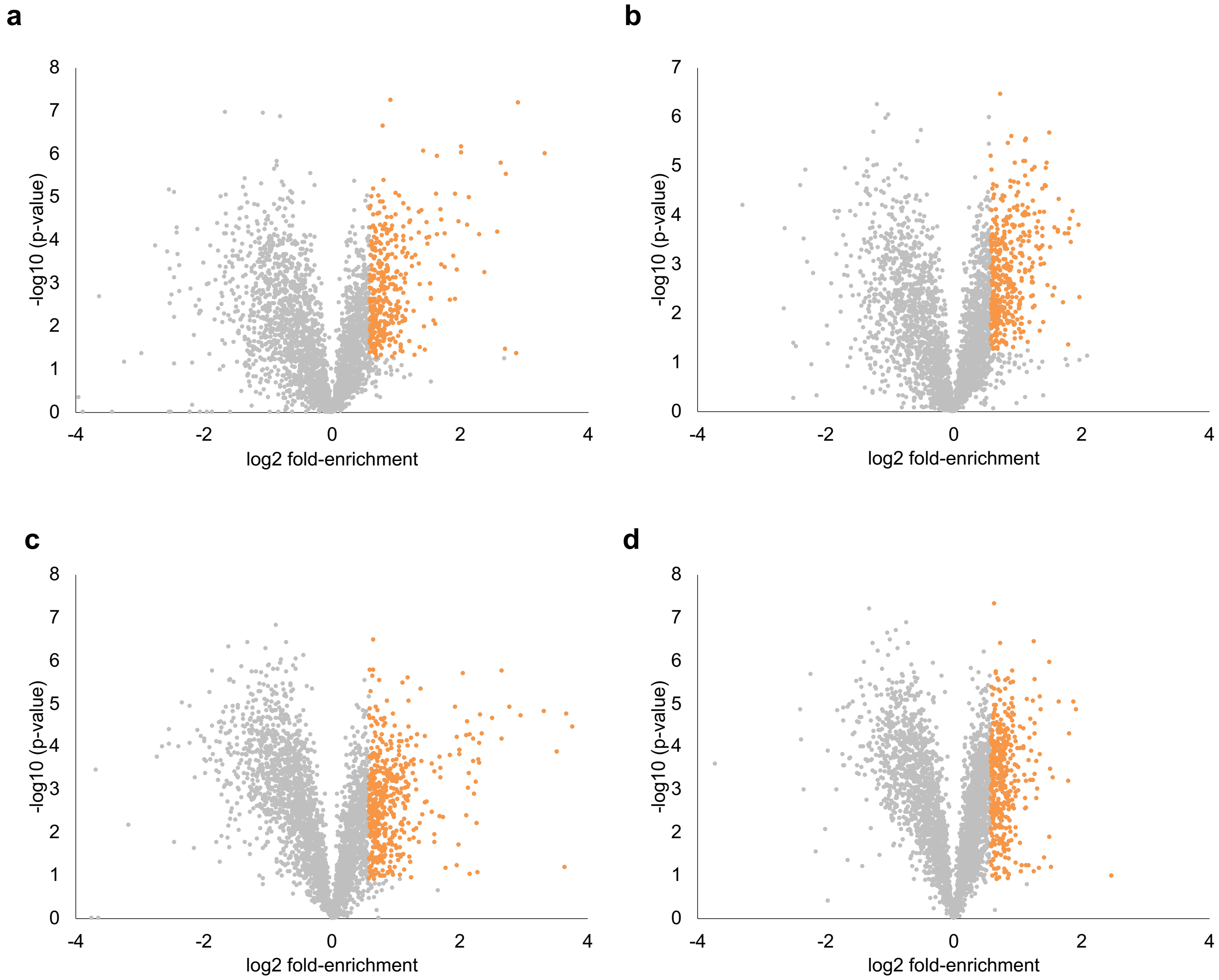

Supplementary Figure 8. Volcano plots showing significantly enriched interactions. (a) N/Citrine (-p50) **(b)** TIR/Citrine (-p50) **(c)** N/Citrine (+p50) **(d)** TIR/Citrine (+p50). We designated proteins as significantly enriched if they had a q-value less than 0.05 and a greater than 1.5-fold enrichment over the Citrine control. Significantly enriched interactors are shown in orange.

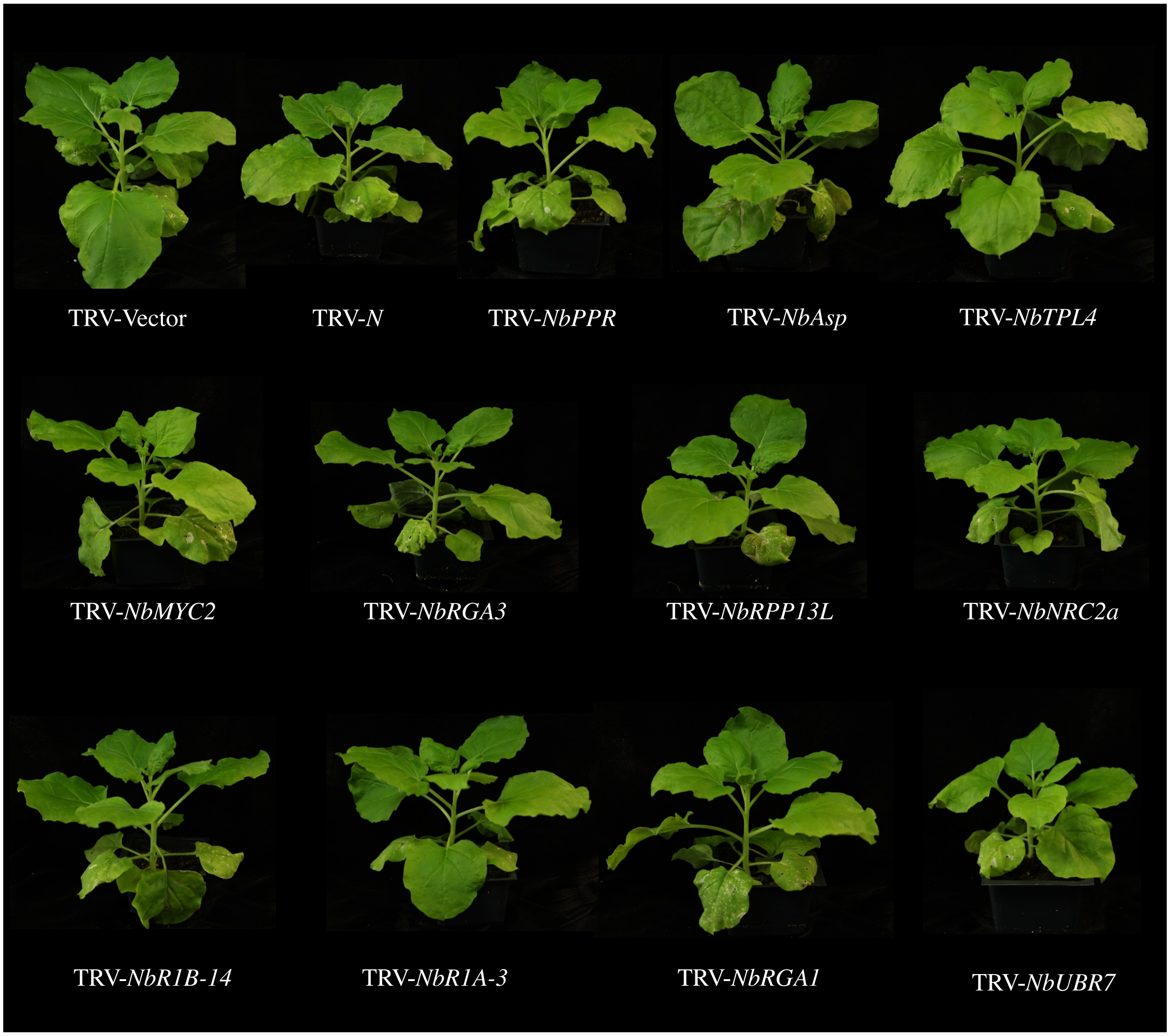

Supplementary Figure 9. Phenotypic observation of the *N*-containing transgenic *N. benthamiana* plants after being silenced with various target genes before infection with TMV. Various recombinant TRV vectors containing different gene fragments were inoculated onto the *N*-containing transgenic *N. benthamiana* plants. Photographs were taken at 14 days post-infiltration and representative pictures are shown. Source data are provided as a Source Data file.

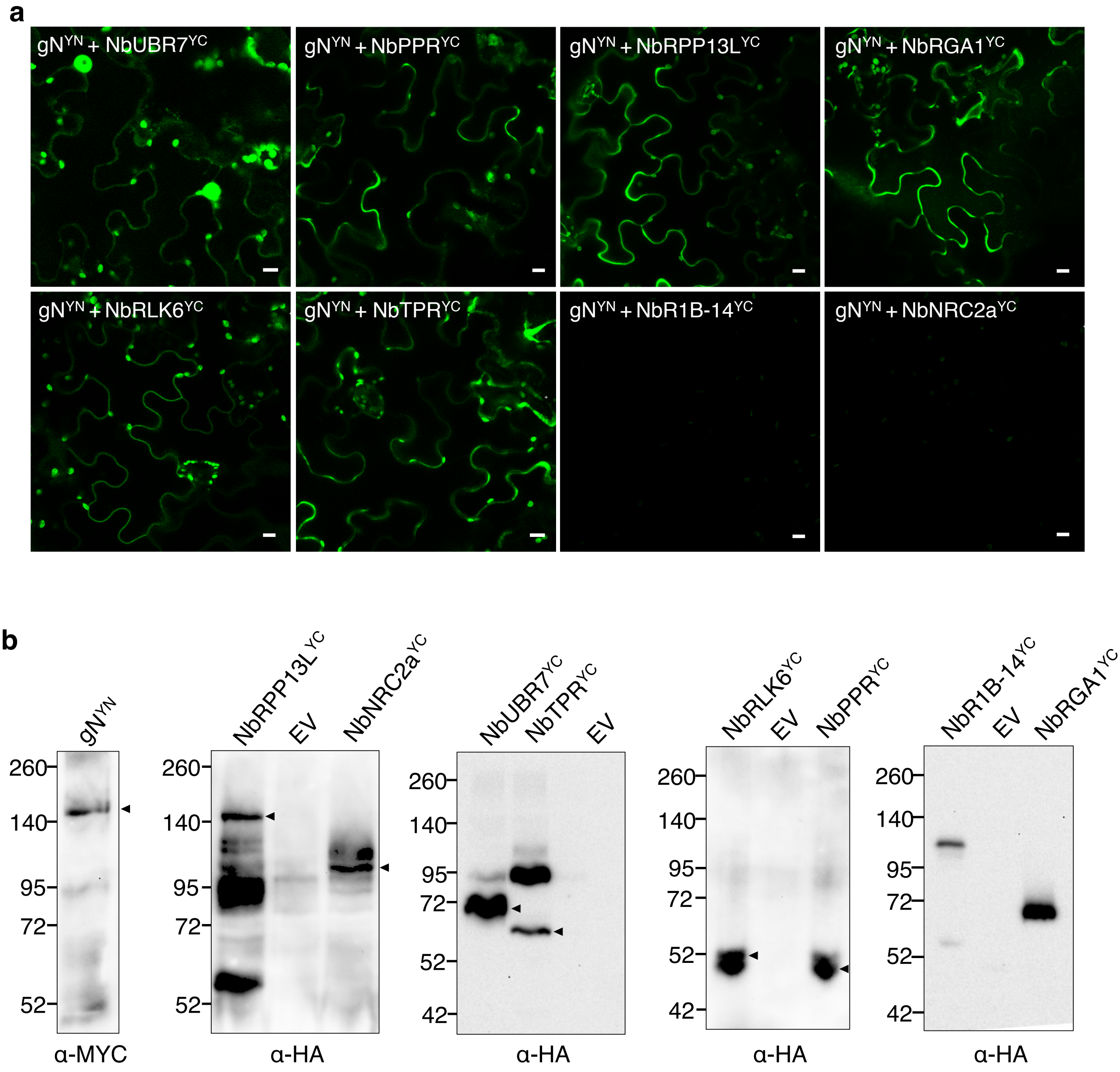

Supplementary Figure 10. Assessment of interaction between TurboID-based PL identified candidates and N NLR using BiFC assay. (a) The identified candidate proteins from the MS dataset were fused to C-terminus of citrine (Candidate protein^{YC}) and co-expressed with N fused to N-terminus of citrine (gN^{YN}) in *N. benthamiana* plants. YC fused to NbNRC2a served as a negative control. Confocal analysis was performed at 46 hpi. All tested candidates except NbR1B-14 showed reconstituted citrine fluorescent signal. Scale bars represent 10 μm. (**b**) Western blot analysis of the protein expression in the infiltrated leaves. Samples used for Western blot were indicated on top of the panels and the antibodies used are indicated below the panels. The molecular weight size markers in kDa are indicated at the left. EV, empty vector.

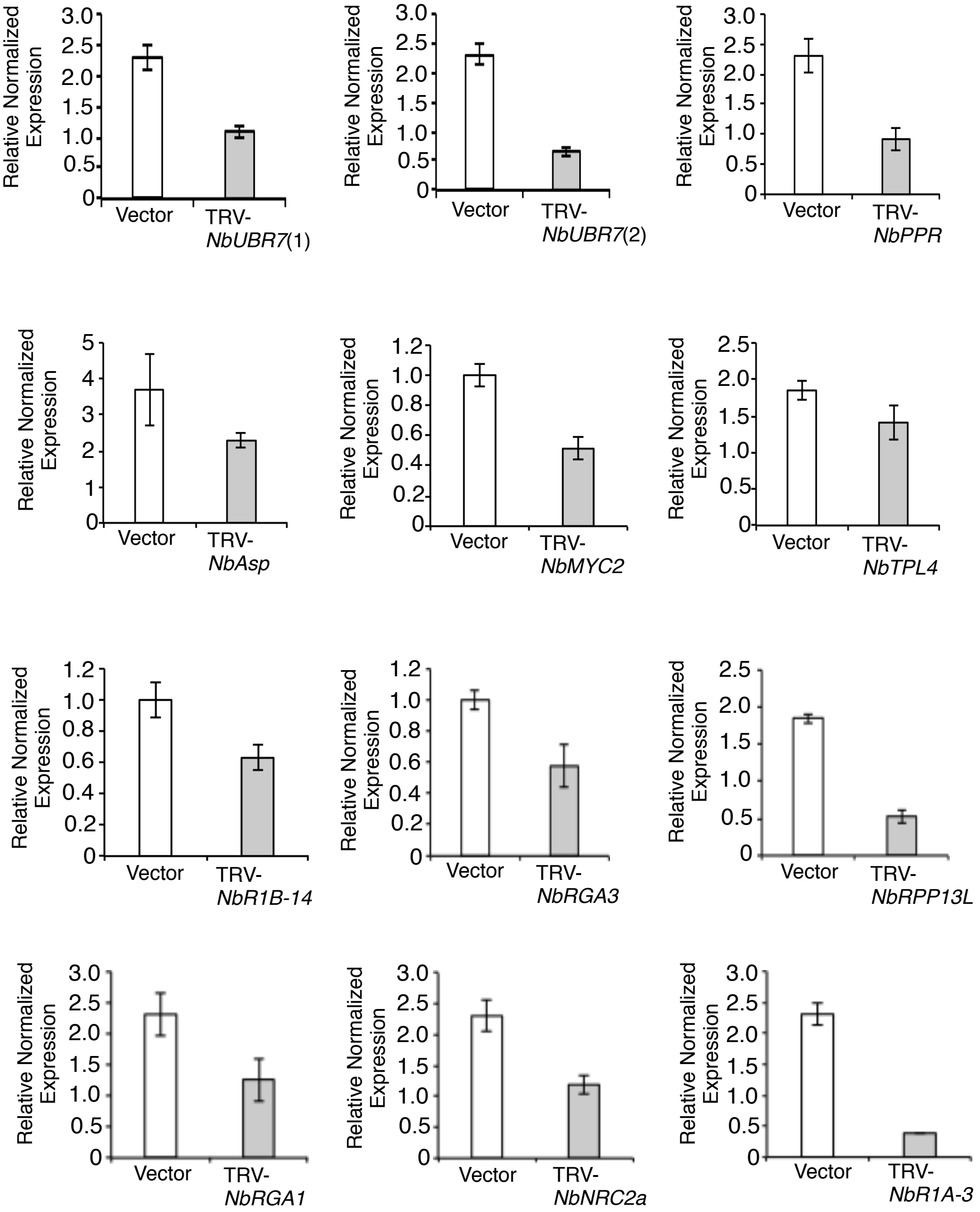

Supplementary Figure 11. Analysis of the effect of silencing on target gene expression in various recombinant TRV vector-inoculated *N*-containing transgenic *N. benthamiana* plants by RT-qPCR. RT-qPCR was performed using gene specific primers, on RNA extracted from plants 10 days post-inoculation with different recombinant TRV vectors. For the analysis of *NbMYC2*, *NbTPL4*, *NbRGA3* and *NbRPP13L* expression, *NbPP2a* was used as a reference, whereas *NbeIF4A was used* as a reference for the analysis of other genes. VIGS vector-inoculated plants served as the control. Data from three biological replicates were combined and values are shown as mean ± SD (n = 3).

```
HsUBR7     1 -MAGAEGAAGRQSELEPVVSLVDVLEEDEELENEACAVLGGSDSEKCSYSQGSVKRQALY
NbUBR7     1 -MADA-----FEEEGDQTVSINEYLEDIEEQELEADLVLGGDEGKECTYSKGYMKRQAIF
AtUBR7     1 MASGV-----FEDEAEGTITINEYIERLDAEELAADLVLGGDEGDECTFPKGYMKRQAIF

HsUBR7    60 ACSTCTPEGEEPAGICLACSYECHGSHKLFELYTKRNFRCDCGNSKFKNLECKLLPDKAK
NbUBR7    55 SCLTCTPDGN--AGVCTACSLSCHDGHEILELWTKRNFRCDCGNSKFGEFFCKLDASKDV
AtUBR7    56 SCITCTPEGN--AGICTACCLSCHDGHELLELWTKRNFRCDCGNSKFGTLACKLLPSKDI

HsUBR7   120 VNSGNKYNDNFFGLYCICKRPYPDPEDEIPDEMIQCVVCEDWFHGRHLGAIPPES-----
NbUBR7   113 ENTENSYNHNFKGSYCTCCRPYPDPDVDDQLENLQCCICEDWFHEEHLGLESSD----MV
AtUBR7   114 ENSENSYNHNFKGLYCTCDRPYPDPNVEEQVEMIQCCLCEDWFHEEHLGLTPSDSVGSQI

HsUBR7   175 -------GDFQEMVCQACMKRCSFLWAYAAQLAVTKISTEDD------GLVRNIDGIGDQ
NbUBR7   169 PRDENGEPQFEDLICQGCAAICSFLKLYPHSIFASVQQHTATNSSKDKEVVE-------D
AtUBR7   174 PRDEESEPIYEDFICQNCSPACSFLTLYPENLWVVAKVDSTGSANACSETIELDKNHMDS

HsUBR7   222 EVIKPENGEHQDSTLKED-----------VPEQGKDDVREVKVEQNS----EPCAGSSSE
NbUBR7   222 APL-----------TVGSSEE---------LNNGSSSIETP-VSEDS--LKKDFTGKAV-
AtUBR7   234 EPGQPENGTDAEKSVVGKCSETISDSEPGQPENGTEAEKSV-VQKCSEKIDESEAGQPEN

HsUBR7   267 S--DLQTVFKNES-----LNAESKSGCKLQ--ELKAKQLIKKDTATYWPLNWRSKLCTCQ
NbUBR7   258 ---GENLVT-NTILNQYNQIAVPSTKCFLGLNLLEAPICLEKSKPMFLSNDWREILCRCT
AtUBR7   293 STEAEKFVVRKCSEKIDGSENVPAAGCVIRTDLNSCPE--FEKKPLFLTKNWRNILCRCE

HsUBR7   318 DCMKMYGDLDVLFLTDEYDTVLAYENKGKIAQATDRS---DPLMDTLSSMNRVQQVELIC
NbUBR7   314 NCTEFYKQKGLAFLLEKEDTIAEYEKMAKQKRAQHEQ---ERSAEMLNKLGHVGKMEVLT
AtUBR7   351 KCLEMYKQRKVSYLLDAEDTIVEYEKKAKEKRTEKLEKQEGEALDLLNNLDHVSKVELLH

HsUBR7   375 EYNDLKTELKDYLKRFADEGTVVKREDIQQFFEEFQSKKRRRVDGMQYYCS
NbUBR7   371 GMADLKDEISNYLASFDPSK-PVTSADVHKIFENLAQKRRRTT--------
AtUBR7   411 GIKDFQDGLQGLMESAGPSK-AITSADIEQMFSKLKNKRKRME--------
```

Supplementary Figure 12. Comparison of UBR7 proteins. Alignment of *N. benthamiana* UBR7 (*Nb*UBR7) and *A. thaliana* UBR7 (*At*UBR7) proteins with human UBR7 (*Hs*UBR7) protein generated using ClustalW2. Shading was performed in BoxShade. Numbers on the left indicate the positions of the amino acid residues. Identical residues are shaded in black and similar residues are shaded in gray. Line region shows UBR box region.

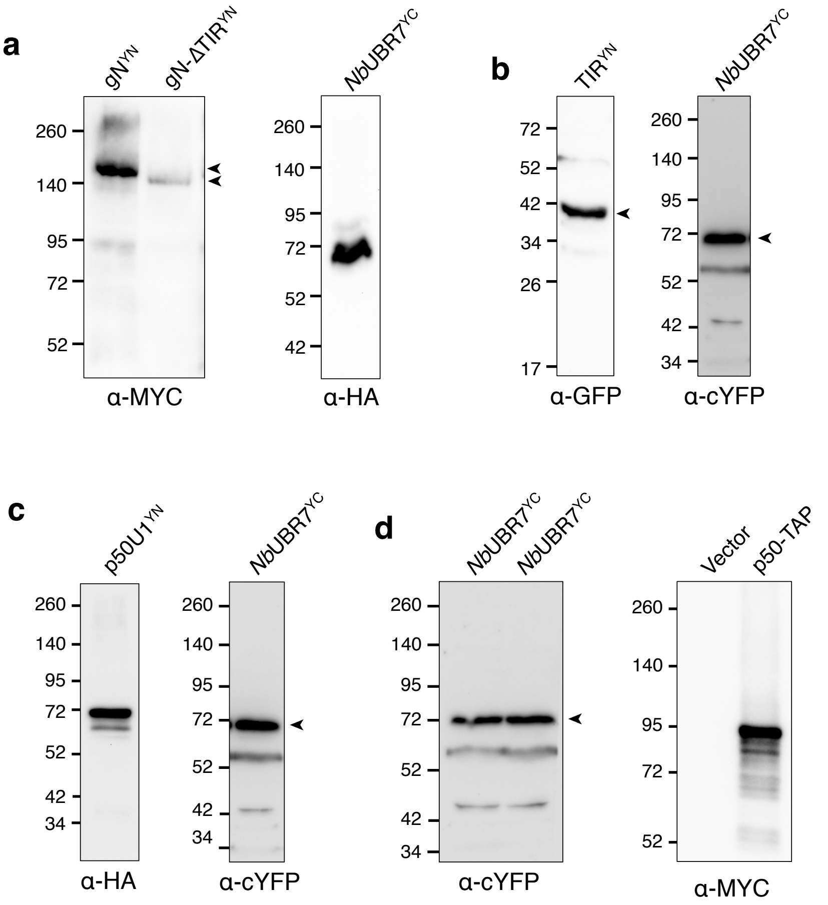

Supplementary Figure 13. Western blot analysis to confirm the protein expression.
a) Expression of gN^{YN} and gN-ΔTIR^{YN} were confirmed by antibodies against MYC tag and $NbUBR7^{YC}$ confirmed by antibodies against HA tag. **b**) Expression of TIR^{YN} and $NbUBR7^{YC}$ were confirmed using antibodies against GFP and C-terminus of YFP respectively.
c) Expression of $p50U1^{YN}$ and $NbUBR7^{YC}$ were confirmed using antibodies against HA tag and C-terminus of YFP respectively. **d**) $NbUBR7^{YC}$ and p50-TAP were confirmed using antibodies against C-terminus of YFP and MYC tag respectively. Source data are provided as a Source Data file.

Supplementary Table 1. N proximal proteins selected for genetic screening and BiFC assay

Gene ID	Annotation	Name	-p50				+p50			
			Fold change N/Citrine	Fold change TIR/Citrine	q-value N/Citrine	q-value TIR/Citrine	Fold change N/Citrine	Fold change TIR/Citrine	q-value N/Citrine	q-value TIR/Citrine
Niben101Scf03903g02005.1	Pentatricopeptide repeat-containing protein	*Nb*PPR	7.35	3.91	0.020	0.060				
Niben101Scf00410g02014.1	PHD finger protein-related	*Nb*UBR7	6.59	2.34	0.000	0.000				
Niben101Scf16939g00004.1	Aspartyl protease family protein	*Nb*Asp	5.19	2.94	0.001	0.003	4.96	1.68	0.001	0.001
Niben101Scf09716g02001.1	putative late blight resistance protein homolog R1B-14	*Nb*R1B-14	1.78	1.62	0.007	0.028	1.50	1.37	0.001	0.001
Niben101Scf14194g00002.1	putative disease resistance protein RGA1-like	*Nb*RGA1	1.52	3.64	0.004	0.000	1.71	2.82	0.001	0.000
Niben101Scf05619g00011.1	putative disease resistance protein RGA3	*Nb*RGA3					0.46	2.25	0.040	0.019
Niben101Scf06889g00007.1	putative disease resistance RPP13-like	*Nb*RPP13L					1.73	2.05	0.001	0.000
Niben101Scf02248g01001.1	late blight resistance protein homolog R1A-3	*Nb*R1A-3	1.47	2.06	0.023	0.004				
Niben101Scf03894g04001.1	MYC2 (based on the tobacco database search ID: A0A1S4BAV2)	*Nb*MYC2	3.83	2.33	0.00	0.00	4.13	1.64	0.00	0.00
Niben101Scf02819g02011.1	Topless related protein 4-like	*Nb*TPL4	1.91	3.10	0.003	0.001				
Niben101Scf01334g04008.1	Receptor-like protein kinase (Pto interacting kinase; Pti1)	*Nb*RLK6	1.89	1.15	0.052	0.541	1.66	2.53	1.00	0.02
Niben101Scf06958g00007.1	Tetratricopeptide repeat (TPR)	*Nb*TPR	2.55	1.63	0.001	0.001	2.16	1.25	0.001	0.0005

Supplementary Table 2. Primers used for the construction of plasmids.

Primer Name	Oligonucleotide sequence (5′–3′)[a]	Description[b]
SP6577	*ATCTA*aggcctATGAAGGACAACACCGTGCCCCTGAAG	BioID 5' *StuI*
SP6581	*GATGA*gagctcCTGCAGCCCGGGGGATCCACTAGT	BioID 3' *SacI*
SP8359	*ATCTA*cctaggATGTTCAAGAACCTGATCTGGCTGAAG	BioID2 5' *Avr*II
SP7012	*GTAGA*cctaggGCTTCTTCTCAGGCTGAACTCGCC	BioID2 3' *Avr*II
SP7011	*ATCTA*cccgggTTCAAGAACCTGATCTGGCTGAAG	BioID2 5′ *Xma*I
SP7012	*GTAGA*cctaggGCTTCTTCTCAGGCTGAACTCGCC	BioID2 3′ *Avr*II
SP7211	*TCATC*cctaggATGAAGGACAACACCGTGCCCCTG	BioID2 5′ *Avr*II
SP6583	*GTAGA*cctaggCTTCTCTGCGCTTCTCAGGGAGAT	BioID2 3′ *Avr*II
SP7027	*ATCTA*aggcctGTGAGCAAGGGCGAGGAGCTGTTC	Citrine 5′ *Stu*I
SP3034	*GCCAT*cccgggCTTGTACAGCTCGTCCATGCCGAGAG	Citrine 3′ *Xma*I
SP7269	*ATCTA*cccgggAAAGACAATACTGTGCCTCTGAAG	TurboID 5′ *Xma*I
SP7270	*GATGA*cctaggCTTTTCGGCAGACCGCAGACTGATTTC	TurboID 3′ *Avr*II
SP7287	*ATCTA*cctaggATGAAAGACAATACTGTGCCTCTGAAGC	TurboID 5′ *Avr*II
SP7402	*ATCTA*ggtaccATGGCATCTTCTTCTTCTTCTTCTAG	NTIR 5′ *Kpn*I
SP6043	*TAGAT*cccgggTCCCCAGATCCCCATAATCCGAAC	NTIR 3′ *Xma*I
SP3038	*TCTGA*ggtaccATGGTGAGCAAGGGCGAGGAGCTGTTC	Citrine 5′ *Kpn*I
SP3034	*GCCAT*cccgggCTTGTACAGCTCGTCCATGCCGAGAG	Citrine 3′ *Xma*I
SP7611	*ATCTA*tctagaATGGCAGATGTAGCAGCAGATGTAG	VIGS: *NbR1B-14* 5′ *Xba*I
SP7612	*GATGA*gagctcAAACCACTGAGCAAATTTATTTTTATC	VIGS: *NbR1B-14* 3′ *Sac*I
SP7613	*ATCTA*tctagaGTAAGTGAGAAAATATTGAAGCAAATTC	VIGS: *NbRGA3* 5′ *Xba*I
SP7614	*GATGA*gagctcAGTGGATAAATTCCCTGGGACTTC	VIGS: *NbRGA3* 3′ *Sac*I
SP7615	*ATCTA*tctagaTTATTATCTGTTGATAAAAATGGTAAAAG	VIGS: *NbRPP13* 5′ *Xba*I
SP7616	*GATGA*gagctcGCCGTAACAAACCAGCAACTGTCTTTAG	VIGS: *NbRPP13* 3′ *Sac*I
SP7617	*ATCTA*tctagaTCAAAGAGCAGAATTTGCCAGAGAAAG	VIGS: *NbRGA1* 5′ *Xba*I
SP7618	*GATGA*gagctcTATTAGAATTCCCTTTCAACCATG	VIGS: *NbRGA1* 3′ *Sac*I
SP7619	*ATCTA*tctagaATGCATCGGAGAACAATAAAGAAAG	VIGS: *NbNRC2a* 5′ *Xba*I
SP7620	*GATGA*gagctcGTTGAAATCATTGAGATCTTGAAG	VIGS: *NbNRC2a* 3′ *Sac*I
SP7621	*ATCTA*tctagaTGGCGTCCATTGGGTTTACGTCGG	VIGS: *NbR1A-3* 5′ *Xba*I
SP7622	*GATGA*gagctcGAGGATCTGAATTGGCATAAGTAG	VIGS: *NbR1A-3* 3′ *Sac*I
SP7623	*ATCTA*tctagaCGAAGGACAAGGAGGTGGTAGAAG	VIGS: *NbUBR7* 5′ *Xba*I
SP7624	*GATGA*gagctcTACACCTACAGAGAATTTCTCTCC	VIGS: *NbUBR7* 3′ *Sac*I
SP7625	*ATCTA*tctagaGCAGATGCAGTTACTTATATCAAAG	VIGS: *NbMYC2* 5′ *Xba*I
SP7626	*GATGA*gagctcAATCTGAAACTCCATTTCTCTAAAC	VIGS: *NbMYC2* 3′ *Sac*I
SP7629	*ATCTA*tctagaTGAAGCTATGCAGATTTTTTATGAAATG	VIGS: *NbPPR* 5′ *Xba*I
SP7630	*GATGA*gagctcATGTCGGAGCATATTGCATACATTTTAATC	VIGS: *NbPPR* 3′ *Sac*I
SP7631	*ATCTA*tctagaCGAGCTAAATTCCCAATATATTAC	VIGS: *NbAsp* 5′ *Xba*I
SP7632	*GATGA*gagctcAAAGAATGGAAGACTCAAAAGTTTTTG	VIGS: *NbAsp* 3′ *Sac*I
SP7655	*ATCTA*tctagaACATGGGATCCAGGGTTGATTATG	VIGS: *NbTPR4* 5′ *Xba*I
SP7656	*GATGA*gagctcACTCATACTAGTTCCAGCAGTTGAAC	VIGS: *NbTPR4* 3′ *Sac*I
SP7702	ggggacaagtttgtacaaaaaagcaggctccATGGCTGATGCATTTGAAGAGGAAG	*NbUBR7*, 5′
SP7703	ggggaccactttgtacaagaaagctgggtaAGTCGTACGCCTGCGCTTCTGGGC	*NbUBR7*, 3′
SP7704	ggggacaagtttgtacaaaaaagcaggctccATGGAAAACTTAATCTCTACTACTTC	*NbMYC2* 5′
SP7705	ggggaccactttgtacaagaaagctgggtaATTATCAATACCACATAATCTTGTC	*NbMYC2* 3′
SP7706	ggggacaagtttgtacaaaaaagcaggctccATGGGCTGCTTCGGTTGTTGTGATG	*NbRLK6* 5′
SP7707	ggggaccactttgtacaagaaagctgggtaCACGTTTGATGTTTCACTAGGTGC	*NbRLK6* 3′
SP7708	ggggacaagtttgtacaaaaaagcaggctccATGTCAAGGTATTCCACATTCGGG	*NbPPR* 5′
SP7709	ggggaccactttgtacaagaaagctgggtaCAGTGATTCATGCCTTAACAAGAC	*NbPPR* 3′
SP7742	ggggacaagtttgtacaaaaaagcaggctccATGGAAGGATTCAAAGTAGATAAATG	*NbTPR* 5′
SP7743	ggggaccactttgtacaagaaagctgggtaATCGAAGTATGCCGCTTCCAATGC	*NbTPR* 3′
SP7887	ggggacaagtttgtacaaaaaagcaggctccATGGCAGATGTAGCAGTAAAATTCC	*NbR1B-14* 5′, genome DNA
SP7888	ggggaccactttgtacaagaaagctgggtaCAATCCGAGATCTGGAGGAAATACAG	*NbR1B-14* 3′
SP7694	ggggacaagtttgtacaaaaaagcaggctccATGGAGATTGGCTTAGCAGTTGGAG	*NbRPP13L* 5′, genome DNA
SP7890	ggggaccactttgtacaagaaagctgggtaCCGGTATTCCTCATCGACTTTTATG	*NbRPP13L* 3′
SP7891	ggggacaagtttgtacaaaaaagcaggctccATGGTACCAGAATATTTTCTCTTC	*NbRGA1* 5′, genome DNA
SP7892	ggggaccactttgtacaagaaagctgggtaTCTCACCTACATCAAAAGTTGATGAG	*NbRGA1* 3′

SP7893	ggggacaagtttgtacaaaaaagcaggctccATGCATCGGAGAACAATAAAGAAAG	*NbNRC2a* 5′, genome DNA
SP7894	ggggaccactttgtacaagaaagctgggtaGAGATCGGGAGGGAATATAGAGAGC	*NbNRC2a* 3′
SP7989	ggggacaagtttgtacaaaaaagcaggctCGAAGGACAAGGAGGTGGTAGAAG	*NbUBR7*, 5′ entry for dsRNA
SP7990	ggggaccactttgtacaagaaagctgggtaTACACCTACAGAGAATTTCTCTCC	*NbUBR7*, 3′ entry for dsRNA
N-TIR-GFP_F	CACCATGAAAACCTGTACTTCCAATCCAATATGGCATCTTCTTCTTCTTCTTCTAGATG	N-TIR-GFP
N-TIR-GFP_R	GCGATCGCGGATCCGTTATCCACTTCCAATTTACTCTAGTAAGGATTCTATTTTCTCTA	N-TIR-GFP
RBOHD-GFP_F	GGAAAACCTGTACTTCCAATCCAATAAGGACATCATCAACAACATGAAAG	RBOHD-C-GFP
RBOHD-GFP_R	CGCGGATCCGTTATCCACTTCCAATCTAGAAGTTCTCTTTGTGGAAGTCA	RBOHD-C-GFP
GST-*Nb*UBR7_F	CTGTACTTCCAATCCAATATGGCTGATGCATTTGAAG	GST-*Nb*UBR7
GST-*Nb*UBR7_R	TTATCCACTTCCAATTTTAAGTCGTACGCCTGCG	GST-*Nb*UBR7

[a]The restriction sites or the attB/attP sites are shown in lower case. Italicized uppercase letters indicate an additional random sequence of 5 bp to ensure efficient DNA cleavage by the restriction enzyme.
[b]5′ and 3′ indicate the primer corresponds to or is complementary to the target gene, the restriction enzymes used for digestion as well as the purpose of the fragments were also shown.

Supplementary Table 3. Primers used for RT-qPCR or RT-PCR analysis.

Primer Name	Oligonucleotide sequence (5′–3′)	Description[a]
SP8026	GATAATCTTAAGGAACTACCAGCTG	*NbR1B-14* 5′
SP8027	GTTGTTTCTTCTTTAGTATTGCTC	*NbR1B-14* 3′
SP8028	TGTCGCTACCAGTTTGGCTTCAAC	*NbRGA3* 5′
SP8029	TCCAACCAATTAAGGGCAATGAGG	*NbRGA3* 3′
SP8064	TCAGAGATTGAAGAGTGGAGAC	*NbRPP13L* 5′
SP8065	TTGGGAAATACTGCACAATAGG	*NbRPP13L* 3′
SP8032	CGTTTAGAAATGGACAAGAGAGAC	*NbRGA1* 5′
SP8033	TCATCAGTTGTTGCATATAACATAC	*NbRGA1* 3′
SP8034	AGCATCAGCTGATCACTTTCCAAG	*NbNRC2a* 5′
SP8035	TTGGTTGAGTTTTGCAACTCCATC	*NbNRC2a* 3′
SP8036	GCAGATGCAGTAGTGAATTTTCTG	*NbR1A-3* 5′
SP8037	GTCTAAGAACCCTTTTAGGTGTTG	*NbR1A-3* 3′
SP8038	CTGATGCATTTGAAGAGGAAGGTG	*NbUBR7* 5′
SP8039	CTTTACTATAGGTGCACTCCTTGC	*NbUBR7* 3′
SP8042	GAAATACGCACAGCACGCGAAGTG	*NbPPR* 5′
SP8043	CTCCTCATCTCAGAAAACAACCTC	*NbPPR* 3′
SP8044	ACACCACCAATGAGTCTCCTCCTG	*NbAsp* 5′
SP8045	GTTGTTGTTGGTGTTGGGGTGGTG	*NbAsp* 3′
SP8058	GTCTGATGCTTCAAGATGTCGTG	*NbMYC2* 5′
SP8059	ATTATCAATACCACGTAATCTTG	*NbMYC2* 3′
SP8066	GCTTCGCCTTGGTTTGTAG	*NbTPL4* 5′
SP8067	TTGTTCAGTGTCGCCTTTTC	*NbTPL4* 3′
qRT-NbeIF4A-F	GCTTTGGTCTTGGCACCTACTC	*NbeIF4A* 5′
qRT-NbeIF4A-R	TGCTCGCATGACCTTTTCAA	*NbeIF4A* 3′
SP8060	GACCCTGATGTTGATGTTCGCT	*NbPP2A* 5′
SP8061	GAGGGATTTGAAGAGAGATTTC	*NbPP2A* 3′
SP6288	*ATCTA*gcatgcATGGCTCTAGTTGTtaaAGGAAAAG	TMV30BGFP MP 5′
SP6289	*GTAGA*gagctcaAAACGAATCCGATTCGGCGACAGTAG	TMV30BGFP MP 3′

[a]5′ and 3′ indicate the primer corresponds to or is complementary to the target gene.
