# Supplementary material for: TurboID-based proximity labeling reveals that UBR7 is a regulator of N NLR immune receptor-mediated immunity
Source: Nat Commun. 2019 Jul 19;10:3252. doi: 10.1038/s41467-019-11202-z (PMC6642208; doi:10.1038/s41467-019-11202-z)
Supplement: Supplementary file 2 — Description of Additional Supplementary Files [file 41467_2019_11202_MOESM2_ESM.pdf]

Description of Additional Supplementary Files

File Name: Supplementary Data 1
Description: Identification of proteins that interact with full length N or the TIR domain of the N protein in the absence of p50 effector using TurboID-based proximity labeling.

File Name: Supplementary Data 2
Description: Identification of proteins that interact with full length N or the TIR domain of the N protein in the presence of p50 effector using TurboID-based proximity labeling.
