# Supplementary material for: TurboID-based proximity labeling reveals that UBR7 is a regulator of N NLR immune receptor-mediated immunity
Source: Nat Commun. 2019 Jul 19;10:3252. doi: 10.1038/s41467-019-11202-z (PMC6642208; doi:10.1038/s41467-019-11202-z)
Supplement: Supplementary file 5 — Reporting Summary [file 41467_2019_11202_MOESM5_ESM.pdf]

nature research

Corresponding author(s): DBPR and #NCOMMS-18-35069A

Last updated by author(s): May 21, 2019

# Reporting Summary

Nature Research wishes to improve the reproducibility of the work that we publish. This form provides structure for consistency and transparency in reporting. For further information on Nature Research policies, see Authors & Referees and the Editorial Policy Checklist .

## Statistics

For all statistical analyses, confirm that the following items are present in the figure legend, table legend, main text, or Methods section.

| n/a | Confirmed | |
|---|---|---|
| ☐ | ☒ | The exact sample size (*n*) for each experimental group/condition, given as a discrete number and unit of measurement |
| ☐ | ☒ | A statement on whether measurements were taken from distinct samples or whether the same sample was measured repeatedly |
| ☐ | ☒ | The statistical test(s) used AND whether they are one- or two-sided<br>*Only common tests should be described solely by name; describe more complex techniques in the Methods section.* |
| ☒ | ☐ | A description of all covariates tested |
| ☐ | ☒ | A description of any assumptions or corrections, such as tests of normality and adjustment for multiple comparisons |
| ☐ | ☒ | A full description of the statistical parameters including central tendency (e.g. means) or other basic estimates (e.g. regression coefficient) AND variation (e.g. standard deviation) or associated estimates of uncertainty (e.g. confidence intervals) |
| ☐ | ☒ | For null hypothesis testing, the test statistic (e.g. *F*, *t*, *r*) with confidence intervals, effect sizes, degrees of freedom and *P* value noted<br>*Give P values as exact values whenever suitable.* |
| ☒ | ☐ | For Bayesian analysis, information on the choice of priors and Markov chain Monte Carlo settings |
| ☐ | ☒ | For hierarchical and complex designs, identification of the appropriate level for tests and full reporting of outcomes |
| ☐ | ☒ | Estimates of effect sizes (e.g. Cohen's *d*, Pearson's *r*), indicating how they were calculated |

*Our web collection on statistics for biologists contains articles on many of the points above.*

## Software and code

Policy information about availability of computer code

| | |
|---|---|
| Data collection | No custom code was used. |
| Data analysis | No custom code was used. |

For manuscripts utilizing custom algorithms or software that are central to the research but not yet described in published literature, software must be made available to editors/reviewers. We strongly encourage code deposition in a community repository (e.g. GitHub). See the Nature Research guidelines for submitting code & software for further information.

## Data

Policy information about availability of data

All manuscripts must include a data availability statement. This statement should provide the following information, where applicable:

- Accession codes, unique identifiers, or web links for publicly available datasets
- A list of figures that have associated raw data
- A description of any restrictions on data availability

Source data for Figure 2 are provided in the paper in Supplementary Data 1 and Supplementary Data 2. The original MS proteomics raw data as well as the MaxQuant output files may be downloaded from MassIVE (http://massive.ucsd.edu) using the identifier: MSV000083018 and MSV000083019.

N. benthamiana sequence v1.0.1 proteome file entitled “Niben101_annotation.proteins.wdesc.fasta” that was downloaded from the SOL Genomics website (ftp://ftp.solgenomics.net/genomes/Nicotiana_benthamiana) or the Nicotiana tabacum (UP000084051)

# Field-specific reporting

Please select the one below that is the best fit for your research. If you are not sure, read the appropriate sections before making your selection.

- [x] Life sciences
- [ ] Behavioural & social sciences
- [ ] Ecological, evolutionary & environmental sciences

For a reference copy of the document with all sections, see nature.com/documents/nr-reporting-summary-flat.pdf

# Life sciences study design

All studies must disclose on these points even when the disclosure is negative.

| | |
|---|---|
| Sample size | Three biological replicate samples were used for proteomics experiments. This is routinely used in the field of omics. For VIGS analyses, five plants per VIGS construct for each biological replicate was used. We and others have routinely used 3-5 plants per replicate for VIGS. |
| Data exclusions | No data exclusion |
| Replication | All replicates were successful. |
| Randomization | TMT labeling was performed as suggested by the manufacturer and the scheme has been included in Figure 2 |
| Blinding | Not blinded. All proteomics samples were prepared from known target proteins and target genes used in VIGS were identified from database searches. |

# Reporting for specific materials, systems and methods

We require information from authors about some types of materials, experimental systems and methods used in many studies. Here, indicate whether each material, system or method listed is relevant to your study. If you are not sure if a list item applies to your research, read the appropriate section before selecting a response.

## Materials & experimental systems

| n/a | Involved in the study | |
|---|---|---|
| [ ] | [x] | Antibodies |
| [x] | [ ] | Eukaryotic cell lines |
| [x] | [ ] | Palaeontology |
| [x] | [ ] | Animals and other organisms |
| [x] | [ ] | Human research participants |
| [x] | [ ] | Clinical data |

## Methods

| n/a | Involved in the study | |
|---|---|---|
| [x] | [ ] | ChIP-seq |
| [x] | [ ] | Flow cytometry |
| [x] | [ ] | MRI-based neuroimaging |

## Antibodies

**Antibodies used**

Mouse monoclonal anti-c-Myc (Santa Cruz; Cat#SC40)
Rabbit polyclonal anti-tagRFP antibody (Evrogen; EVN-AB234)
Rabbit polyclonal anti-Tag(CGY)FP antibody (Evrogen;EVN-AB121)
Rat anti-HA (Roche; Cat#11867423001)
Rabbit polyclonal anti-PEPC (Abcam; Cat#ab34793)
Streptavidin-HRP (Abcam;Cat#ab7403)
Rat monoclonal anti-HA-HRP [Roche (3F10); Cat#12013819001]
Mouse monoclonal anti-MYC-HRP (Sigma-Aldrich, Cat#16-213)
Goat anti-mouse IgG (Sigma-Aldrich; Cat#A4416)
Goat anti-rat IgG (Santa Cruz; Cat#sc-2065)
Goat Anti-rabbit IgG (Sigma-Aldrich; Cat#A0545)
Mouse monoclonal anti-GST (Santa Cruz; Cat#SC374171)
Rabbit polyclonal anti-GFP (Abcam; ab290)
Rabbit polyclonal anti C-YFP (Agrisera; AS111775)
mAb-anti HA-agarose antibody (Sigma-Aldrich; A2095)

**Validation**

Validations are available from the manufacturer and we also validated by overexpressing fusion proteins followed by Western blot analyses.
