# Supplementary material for: TurboID-based proximity labeling reveals that UBR7 is a regulator of N NLR immune receptor-mediated immunity
Source: Nat Commun. 2019 Jul 19;10:3252. doi: 10.1038/s41467-019-11202-z (PMC6642208; doi:10.1038/s41467-019-11202-z)

Fig. 1b

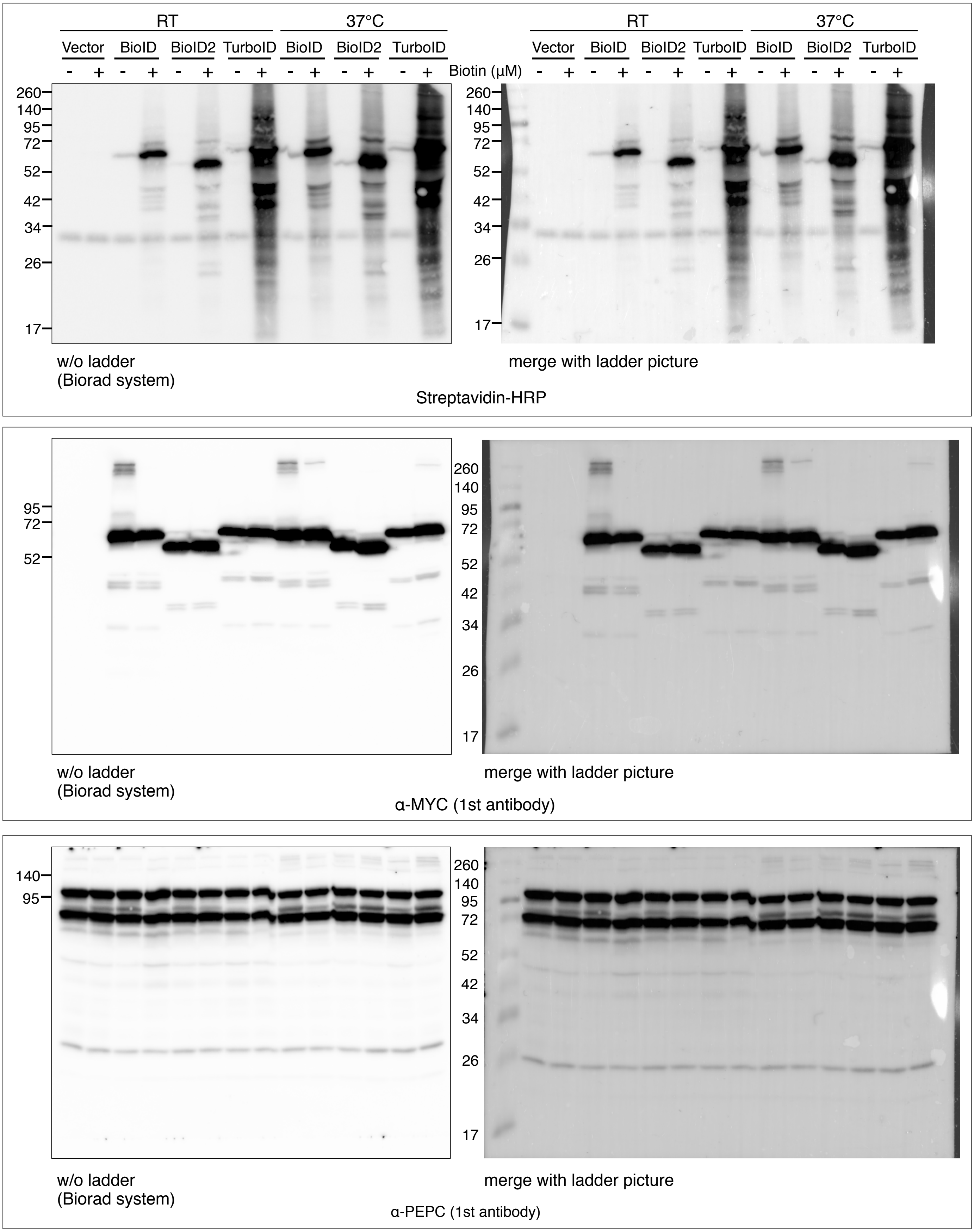
RT
37°C
Vector
BioID
BioID2
TurboID
BioID
BioID2
TurboID
Biotin (μM)
- + - + - + - + - + - + - +
260
140
95
72
52
42
34
26
17
w/o ladder
(Biorad system)
merge with ladder picture
Streptavidin-HRP
w/o ladder
(Biorad system)
merge with ladder picture
α-MYC (1st antibody)
w/o ladder
(Biorad system)
merge with ladder picture
α-PEPC (1st antibody)

Fig. 1c

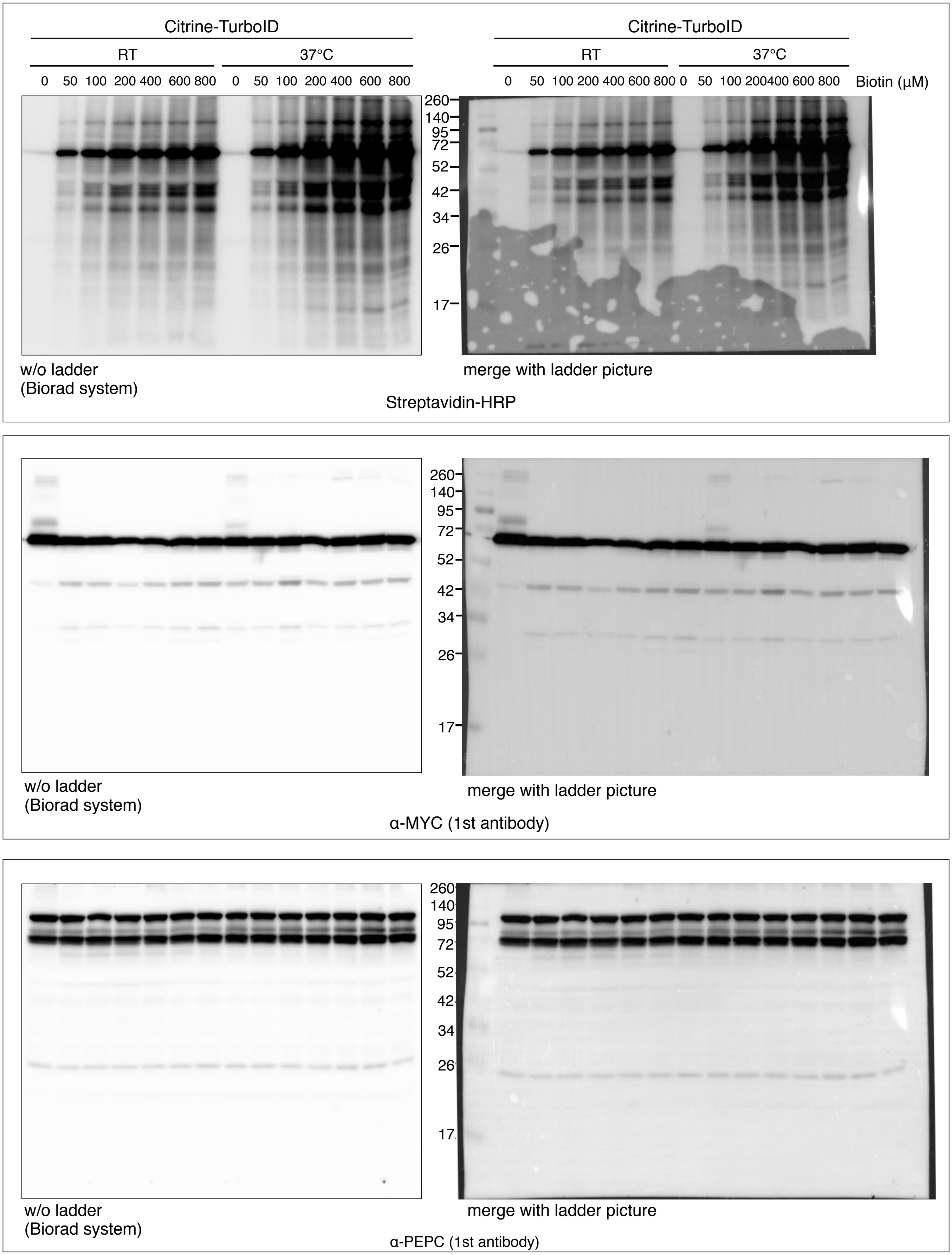
Citrine-TurboID
RT
37°C
0 50 100 200 400 600 800 0 50 100 200 400 600 800
Citrine-TurboID
RT
37°C
0 50 100 200 400 600 800 0 50 100 200400 600 800 Biotin (µM)
260
140
95
72
52
42
34
26
17
w/o ladder
(Biorad system)
merge with ladder picture
Streptavidin-HRP
260
140
95
72
52
42
34
26
17
w/o ladder
(Biorad system)
merge with ladder picture
α-MYC (1st antibody)
260
140
95
72
52
42
34
26
17
w/o ladder
(Biorad system)
merge with ladder picture
α-PEPC (1st antibody)

Fig. 1d

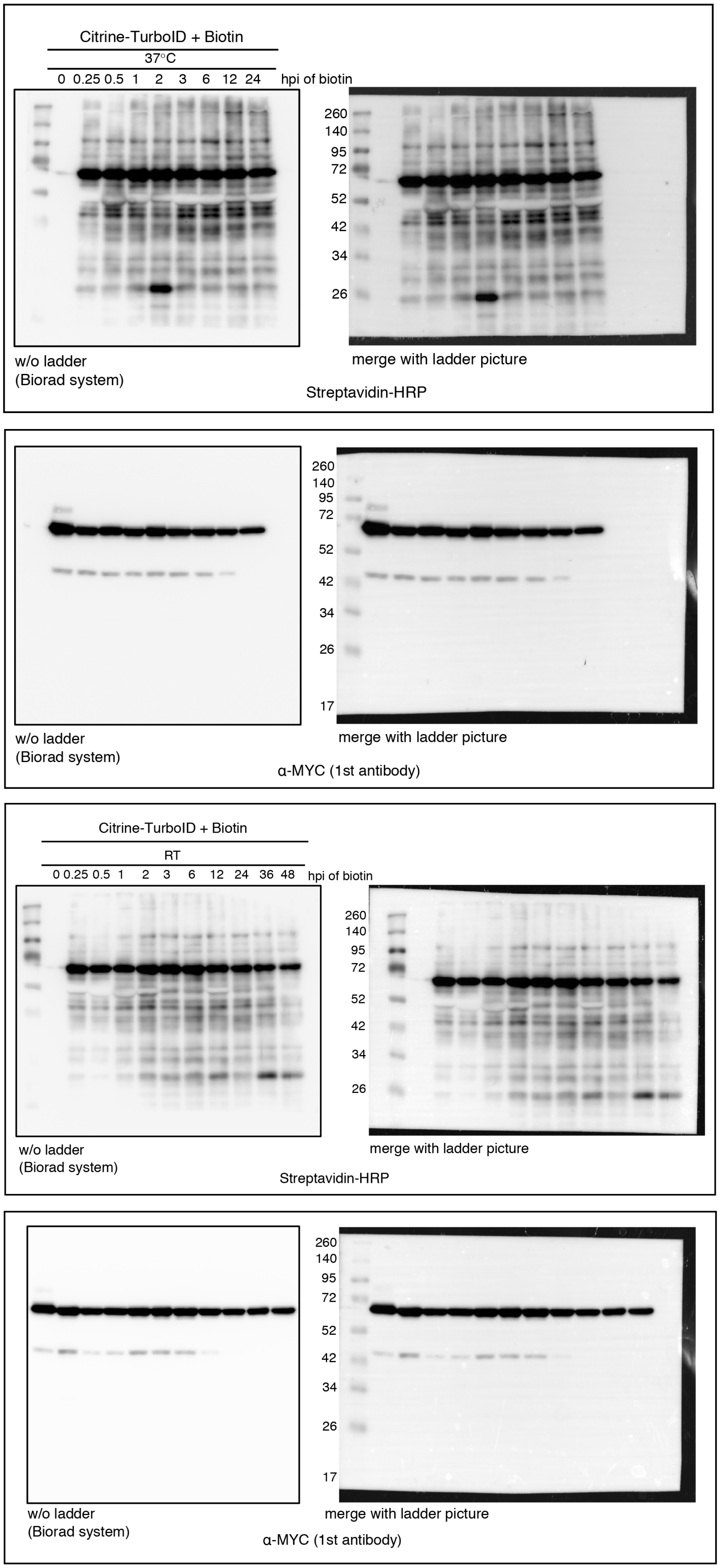
Citrine-TurboID + Biotin
37°C
0 0.25 0.5 1 2 3 6 12 24 hpi of biotin
260
140
95
72
52
42
34
26
w/o ladder
(Biorad system)
merge with ladder picture
Streptavidin-HRP
260
140
95
72
52
42
34
26
17
w/o ladder
(Biorad system)
merge with ladder picture
α-MYC (1st antibody)
Citrine-TurboID + Biotin
RT
0 0.25 0.5 1 2 3 6 12 24 36 48 hpi of biotin
260
140
95
72
52
42
34
26
w/o ladder
(Biorad system)
merge with ladder picture
Streptavidin-HRP
260
140
95
72
52
42
34
26
17
w/o ladder
(Biorad system)
merge with ladder picture
α-MYC (1st antibody)

Fig. 3b

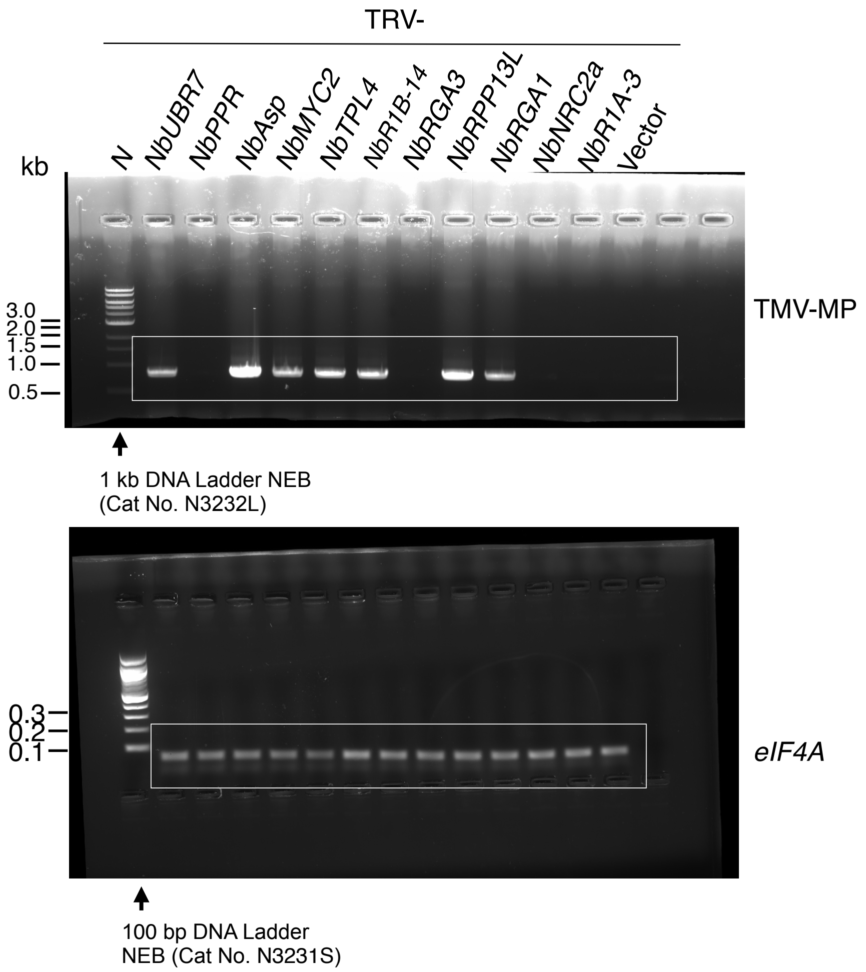
TRV-
N
NbUBR7
NbPPR
NbAsp
NbMYC2
NbTPL4
NbR1B-14
NbRGA3
NbRPP13L
NbRGA1
NbNRC2a
NbR1A-3
Vector
kb
3.0
2.0
1.5
1.0
0.5
TMV-MP
1 kb DNA Ladder NEB
(Cat No. N3232L)
0.3
0.2
0.1
eIF4A
100 bp DNA Ladder
NEB (Cat No. N3231S)

Fig. 3d

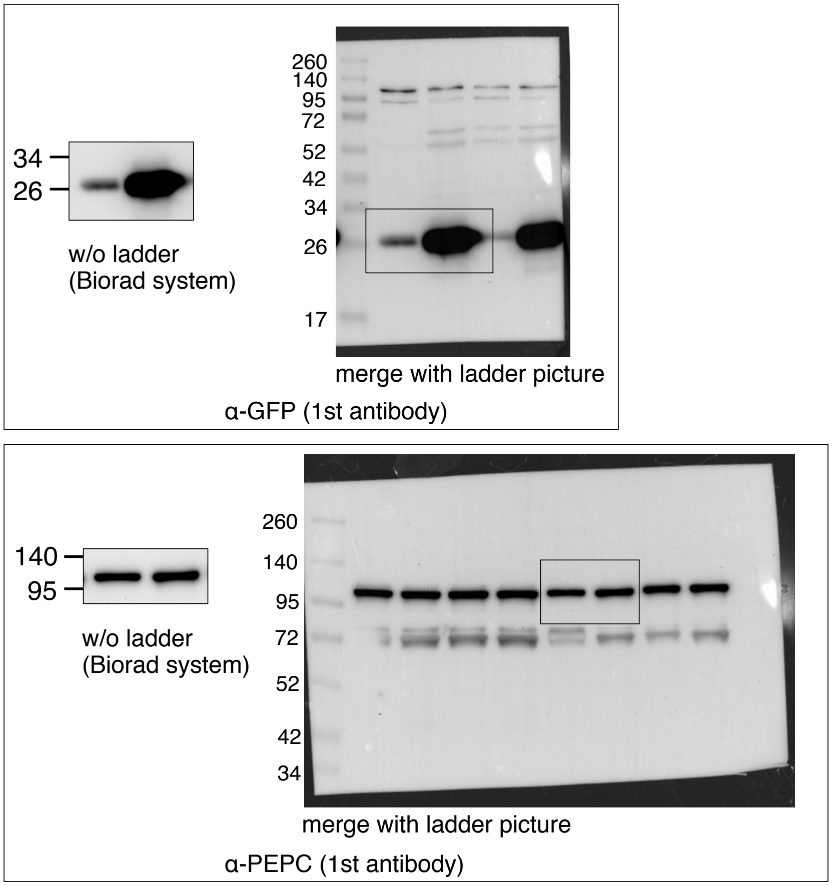
34
26
w/o ladder
(Biorad system)
260
140
95
72
52
42
34
26
17
merge with ladder picture
α-GFP (1st antibody)
140
95
w/o ladder
(Biorad system)
260
140
95
72
52
42
34
merge with ladder picture
α-PEPC (1st antibody)

Fig. 4a

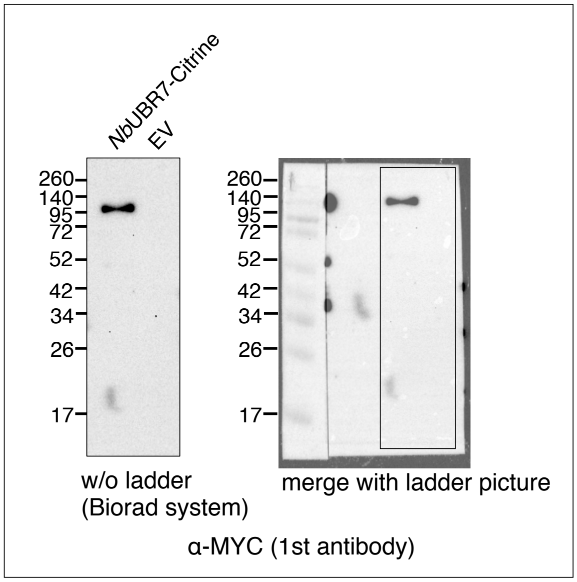

NbUBR7-Citrine
EV
260
140
95
72
52
42
34
26
17
w/o ladder
(Biorad system)
merge with ladder picture
α-MYC (1st antibody)

Fig. 4c

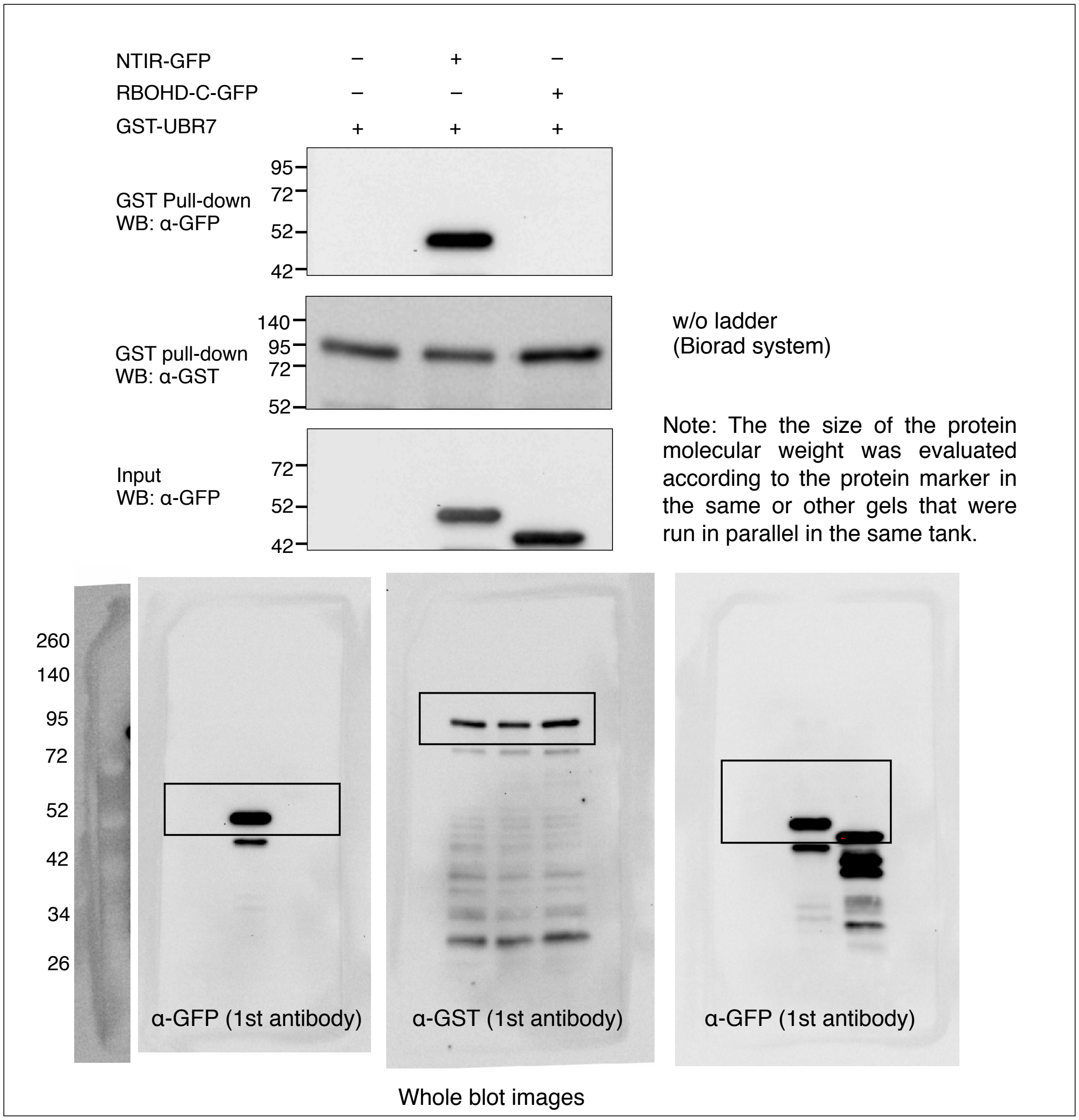

NTIR-GFP – + –
RBOHD-C-GFP – – +
GST-UBR7 + + +
GST Pull-down
WB: α-GFP
95
72
52
42
GST pull-down
WB: α-GST
140
95
72
52
Input
WB: α-GFP
72
52
42
w/o ladder
(Biorad system)
Note: The the size of the protein molecular weight was evaluated according to the protein marker in the same or other gels that were run in parallel in the same tank.
260
140
95
72
52
42
34
26
α-GFP (1st antibody)
α-GST (1st antibody)
α-GFP (1st antibody)
Whole blot images

Fig. 5a

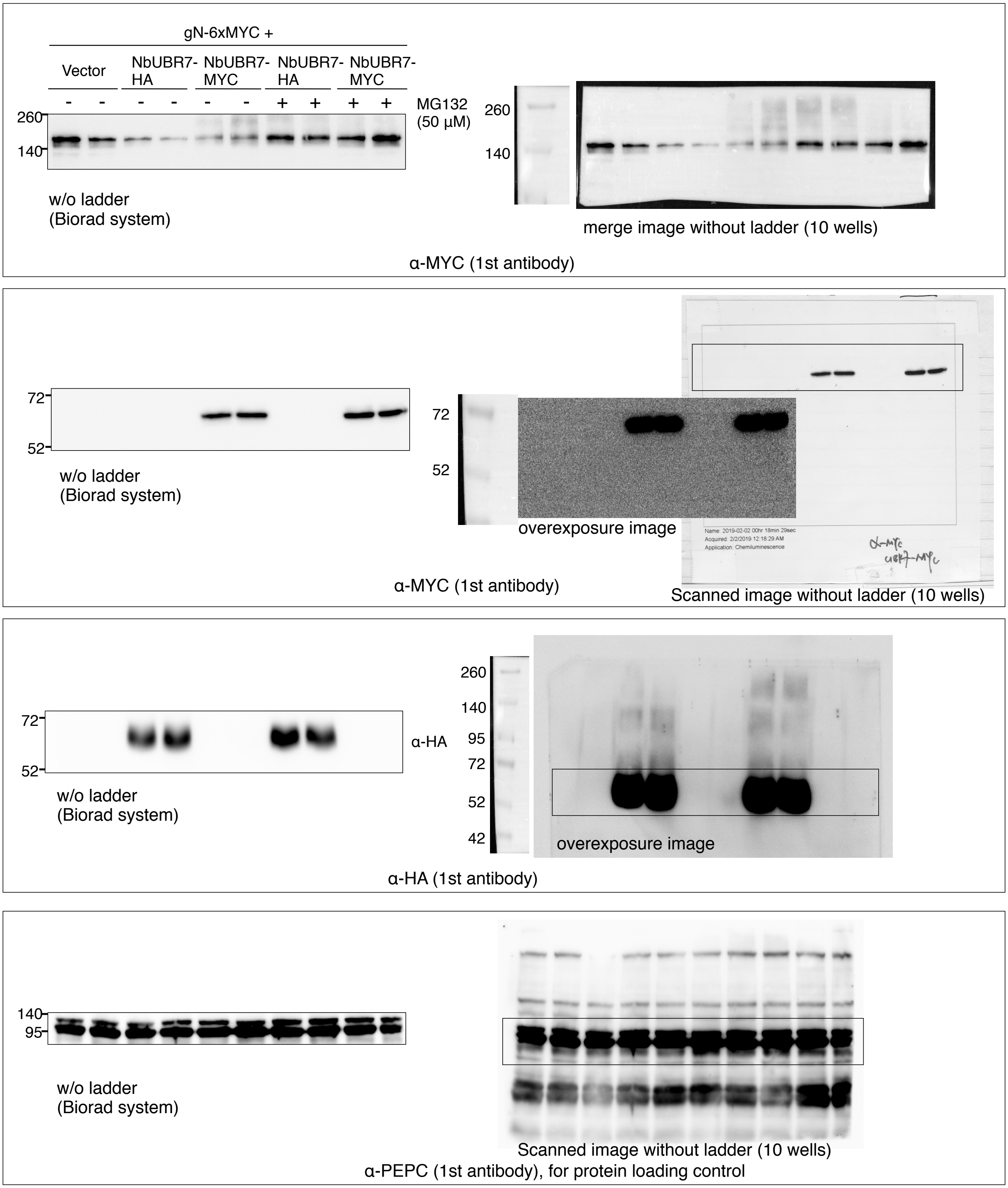

Note: 10 protein samples were loading into the 10 wells, and there is no additional well for loading the protein marker. Therefore, we evaluate the the size of the protein according to the protein marker in another gel that was run in parallel in the same tank. Some gel images were overexposed to show the outline of the blot. The corresponding cropped areas are indicated.

Fig. 5b

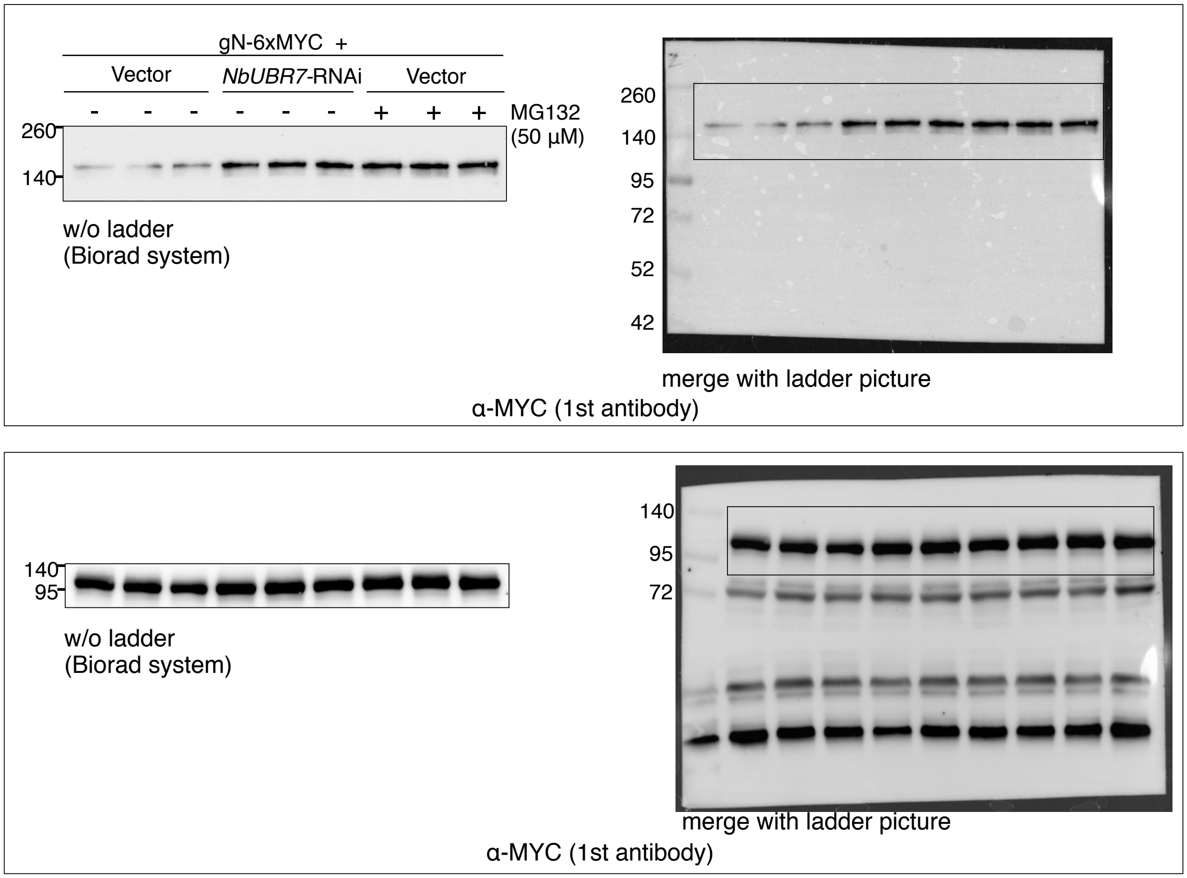

Fig. 5c

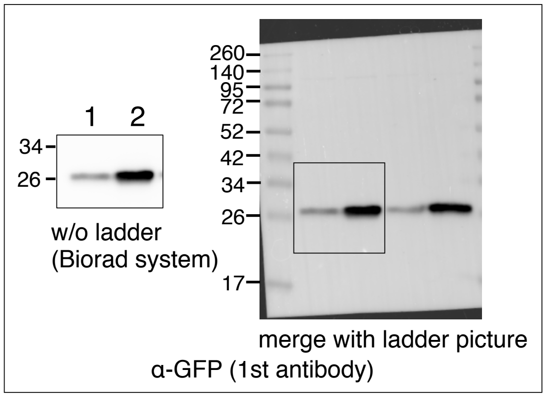

Fig. 5d

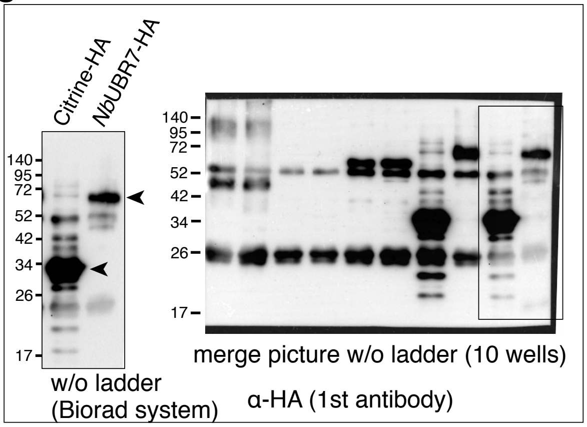

Fig. 5c

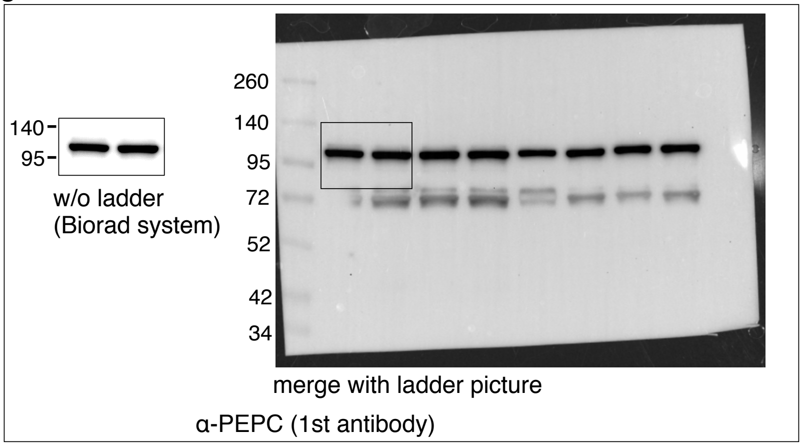

Note: 10 protein samples were loading into the 10 wells, and there is no additional well for loading the protein marker. Therefore, we evaluate the the size of the protein according to the protein marker in another gel that was run in parallel in the same tank. Some gel images were overexposed to show the outline of the blot. The corresponding cropped areas are indicated.

Figure 6b

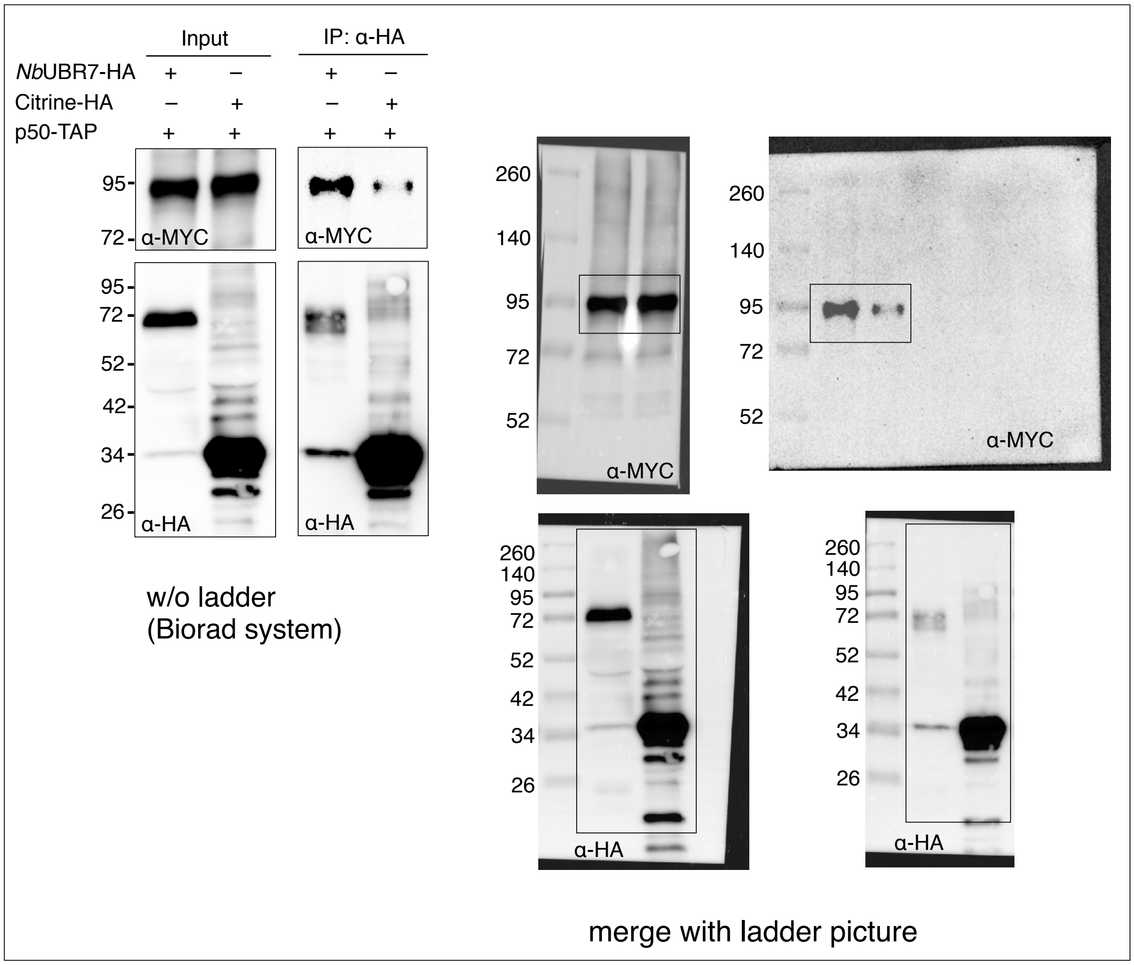

Input
IP: α-HA
NbUBR7-HA + – + –
Citrine-HA – + – +
p50-TAP + + + +
95
72
α-MYC
α-MYC
95
72
52
42
34
26
α-HA
α-HA
w/o ladder
(Biorad system)
260
140
95
72
52
α-MYC
260
140
95
72
52
α-MYC
260
140
95
72
52
42
34
26
α-HA
260
140
95
72
52
42
34
26
α-HA
merge with ladder picture

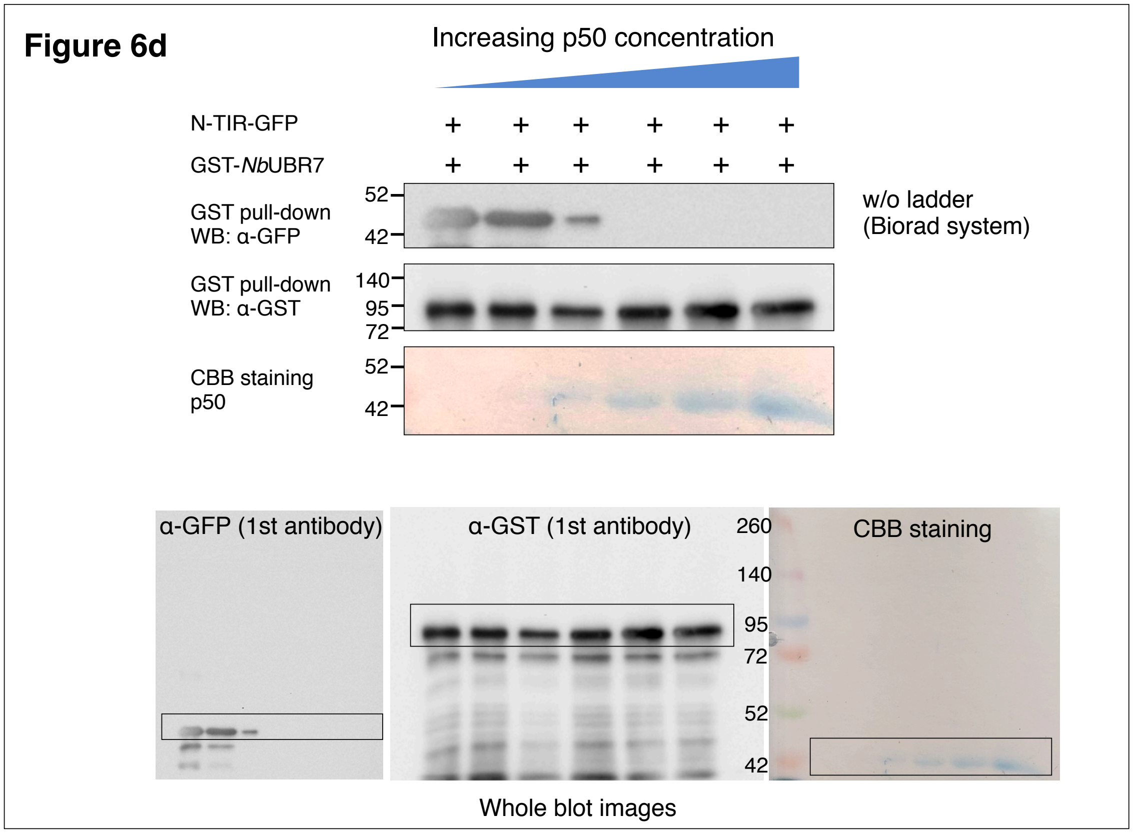

Figure 6d
Increasing p50 concentration
N-TIR-GFP + + + + + +
GST-NbUBR7 + + + + + +
GST pull-down
WB: α-GFP
52
42
GST pull-down
WB: α-GST
140
95
72
CBB staining
p50
52
42
w/o ladder
(Biorad system)
α-GFP (1st antibody)
α-GST (1st antibody)
260
140
95
72
52
42
CBB staining
Whole blot images

Supplementary Fig. 1a

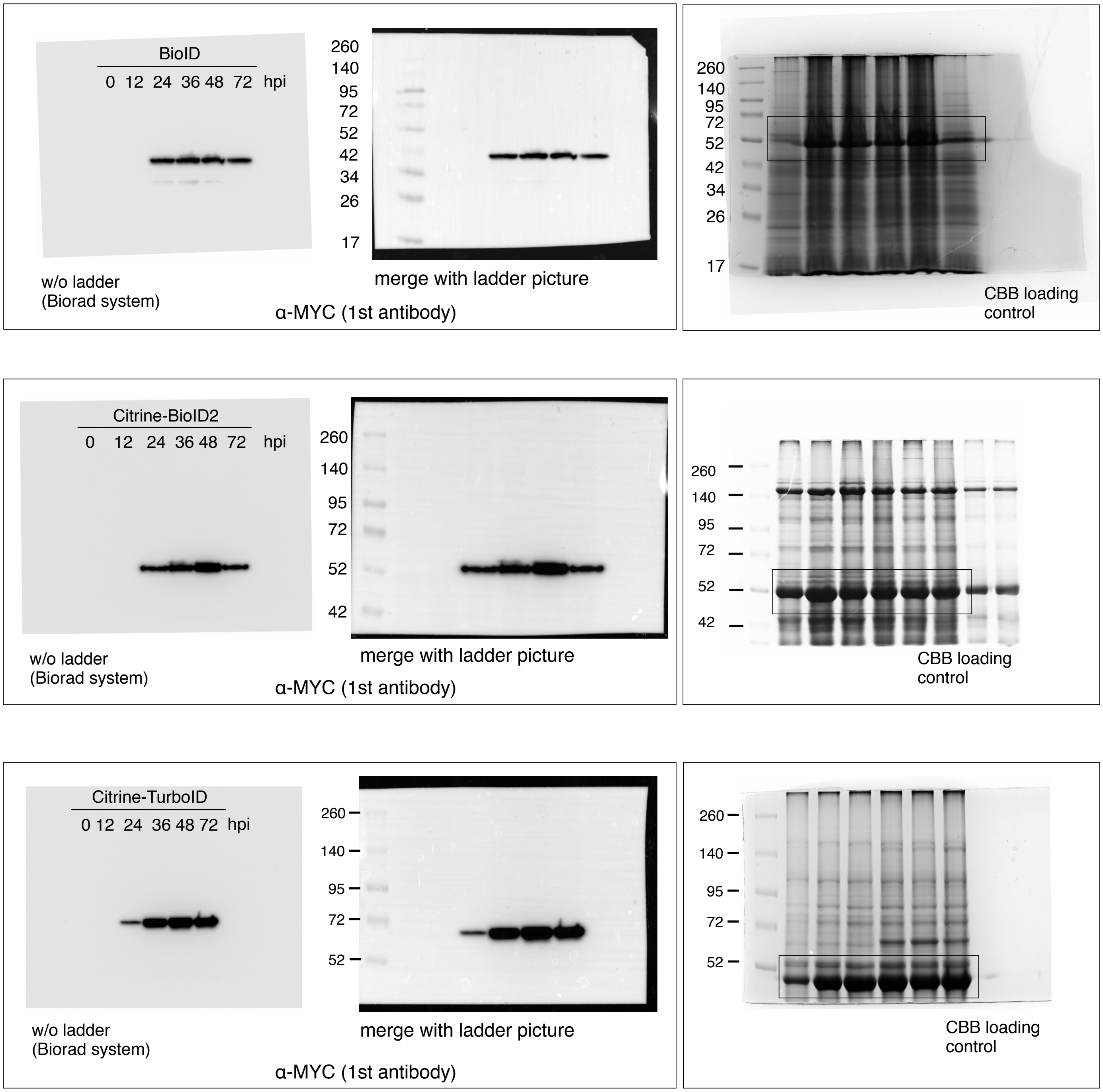

Supplementary Fig. 1b

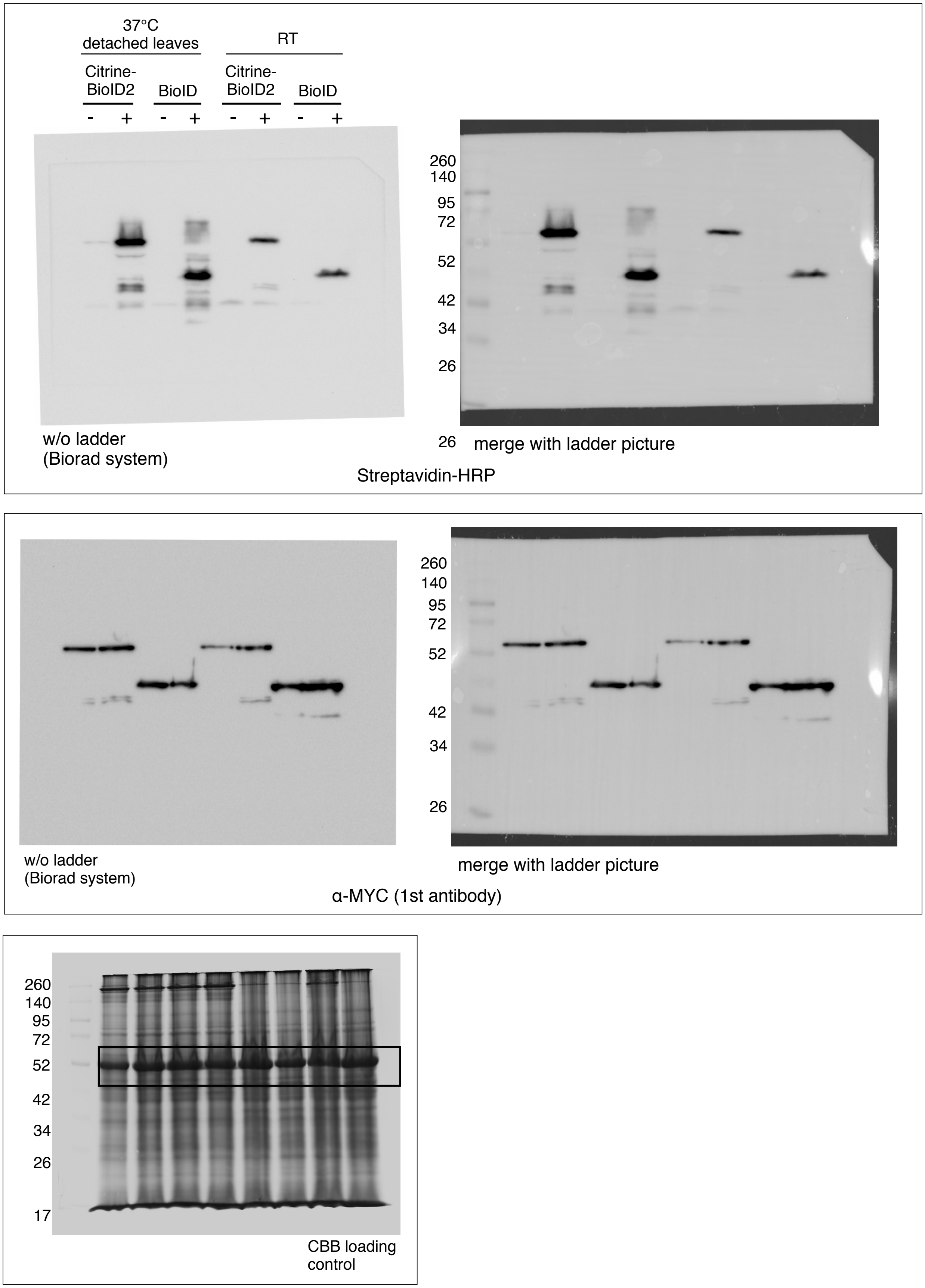

Supplementary Fig. 1b

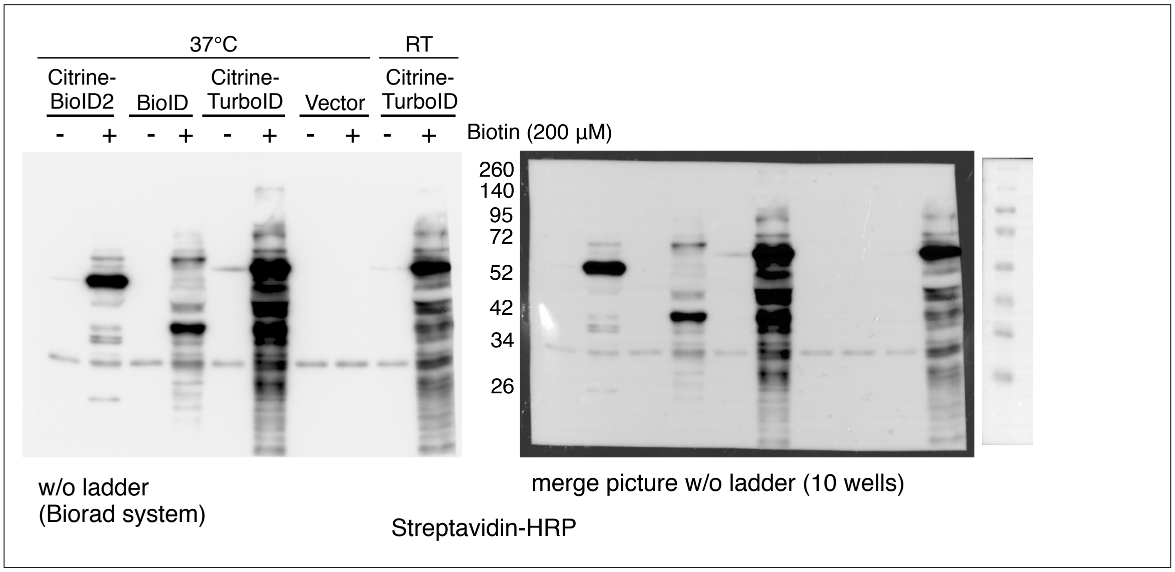

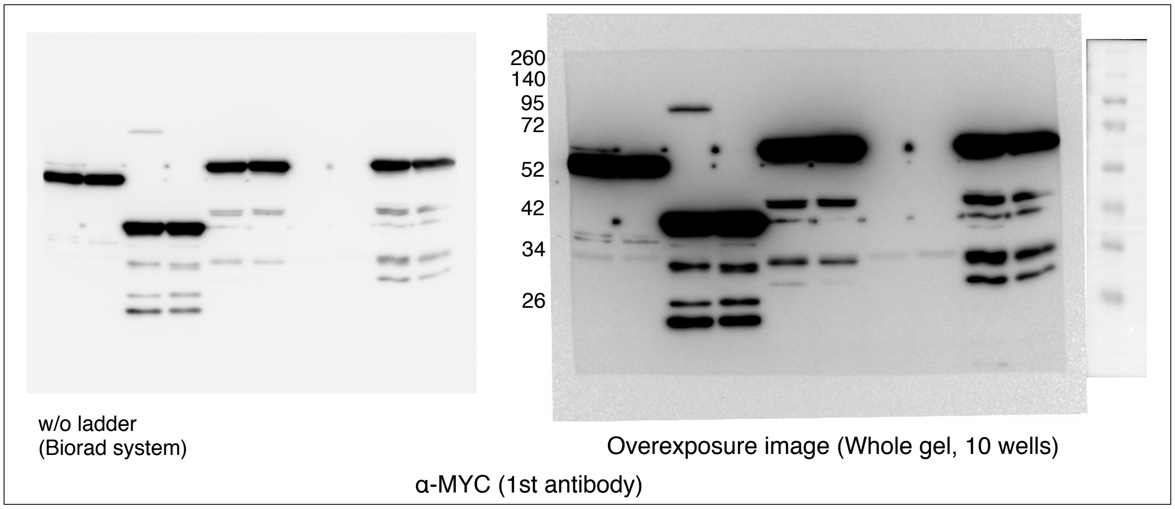

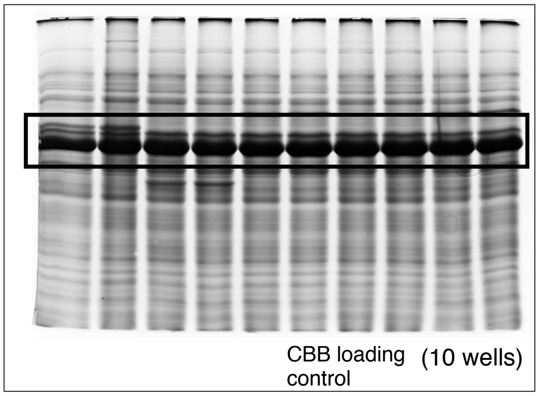

Note: 10 protein samples were loading into the 10 wells, and there is no additional well for loading the protein marker. Therefore, we evaluate the the size of the protein according to the protein marker in another gel that was run in parallel in the same tank. Some gel images were overexposed to show the outline of the blot. The corresponding cropped areas are indicated.

Supplementary Fig. 1c

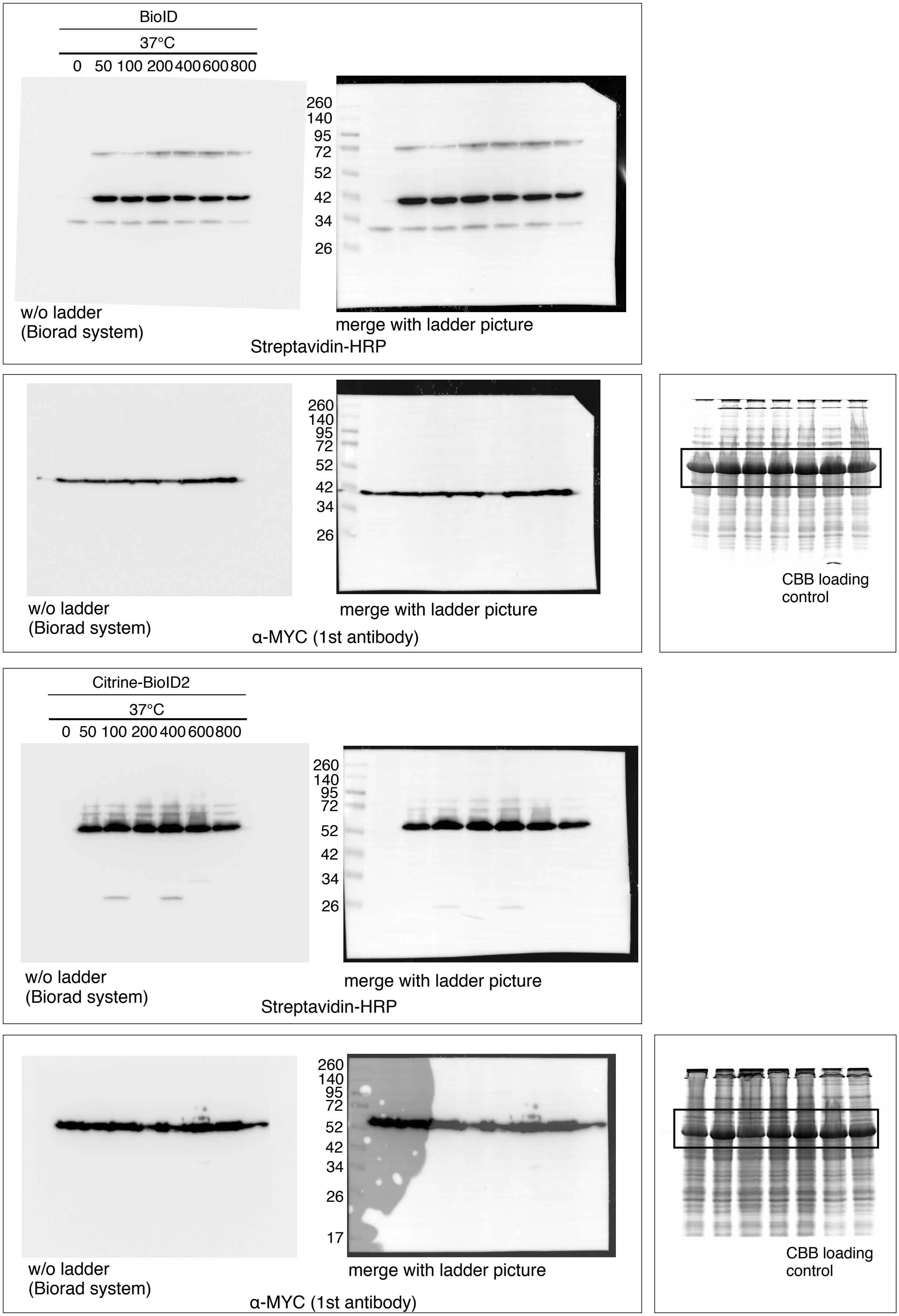

Supplementary Fig. 1c

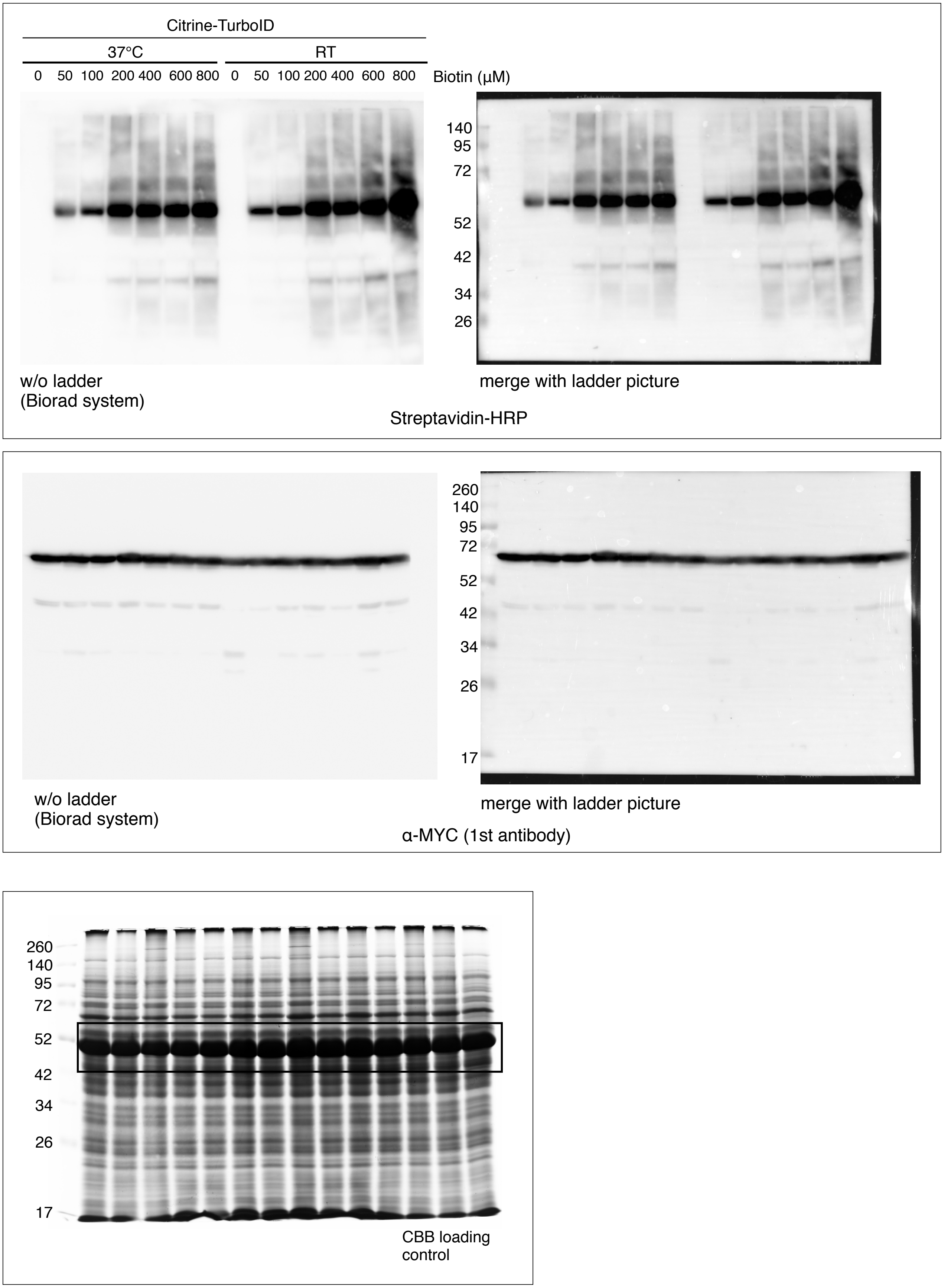

Supplementary Fig. 1d

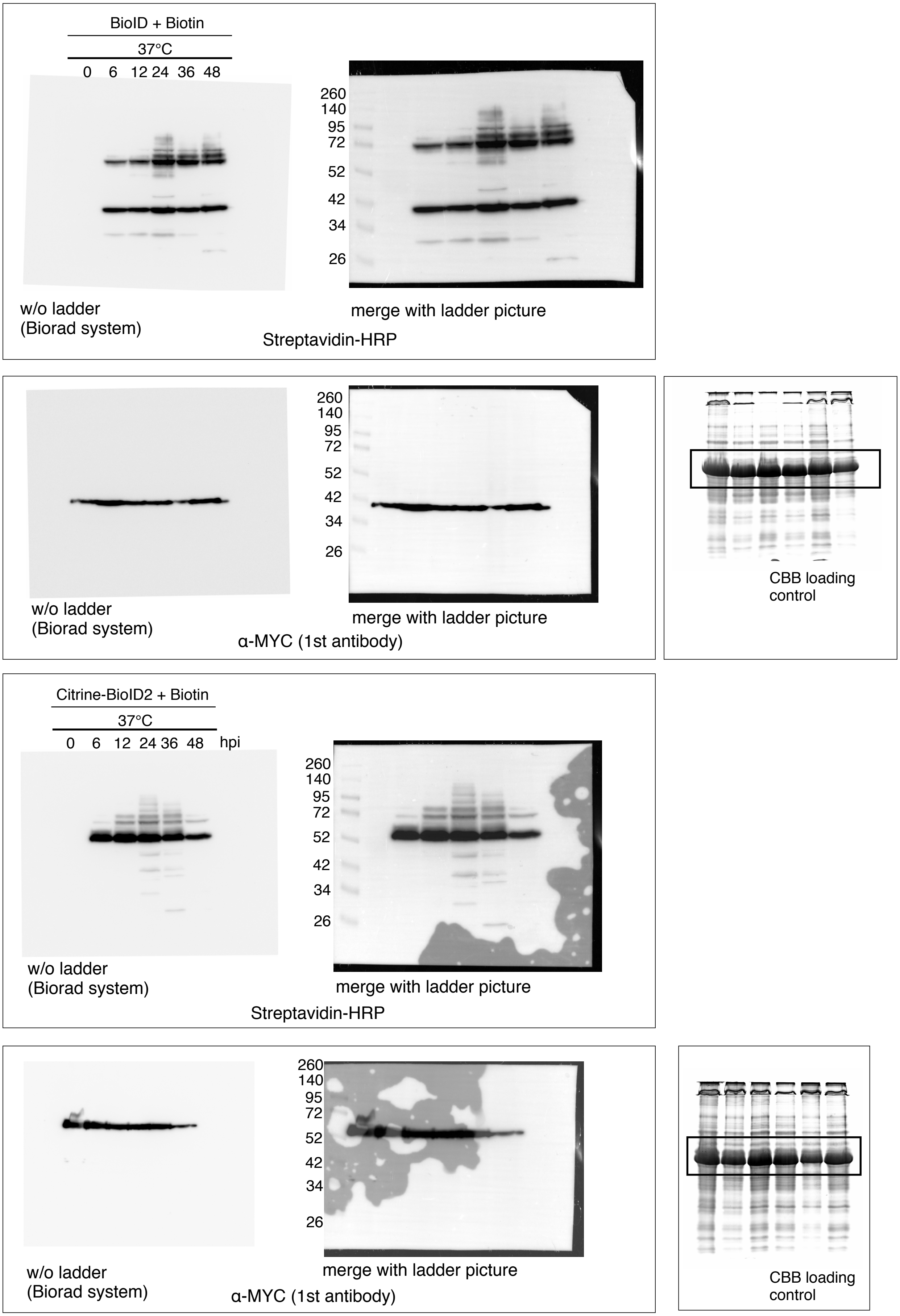

Supplementary Fig. 4b

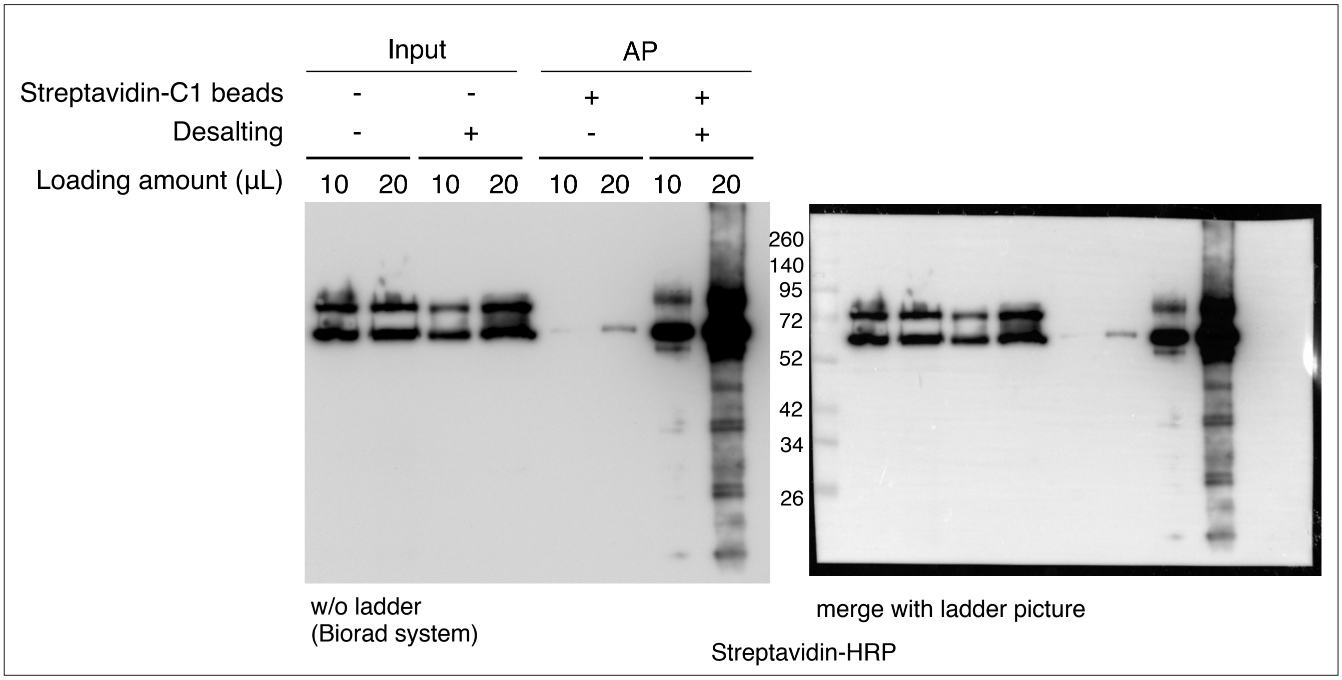

Supplementary Fig. 4d

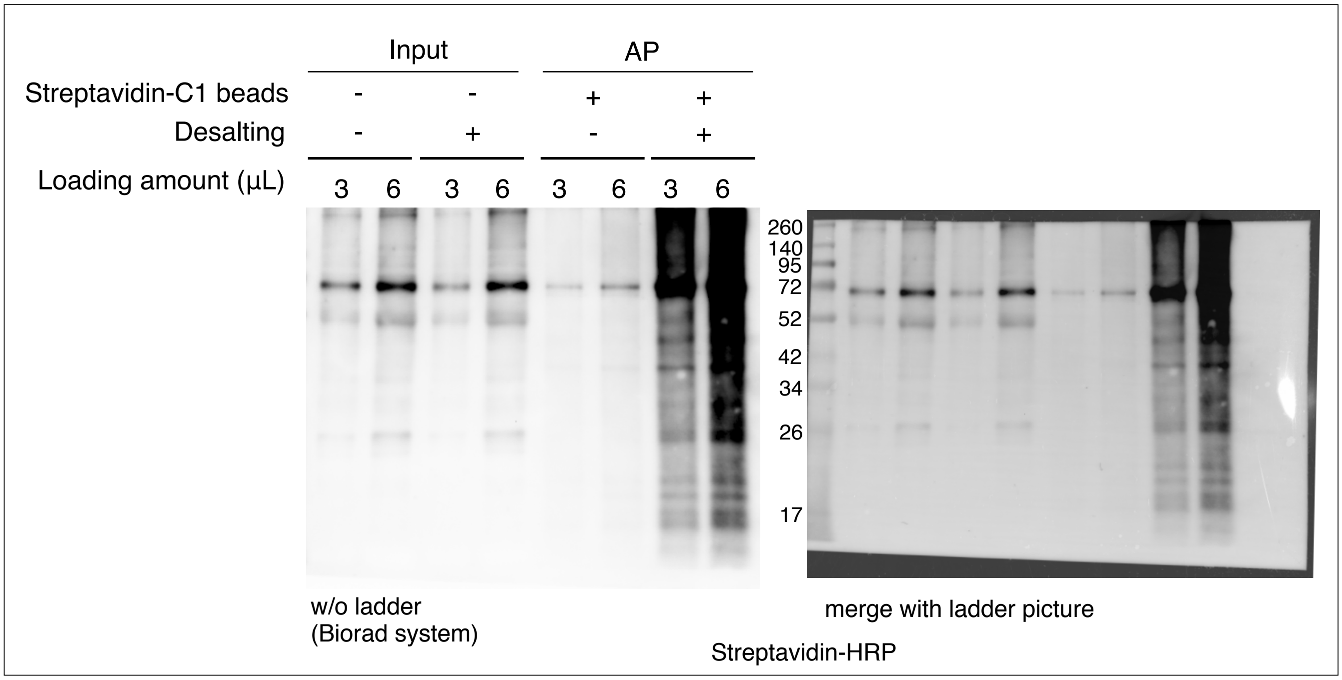

Supplementary Fig. 5c

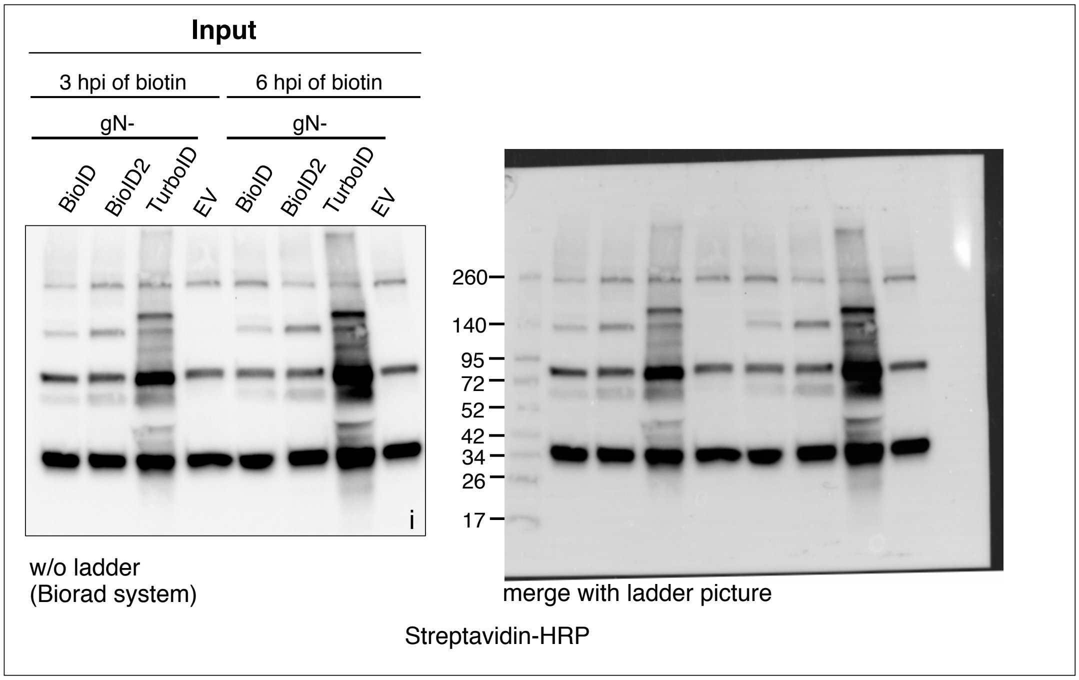

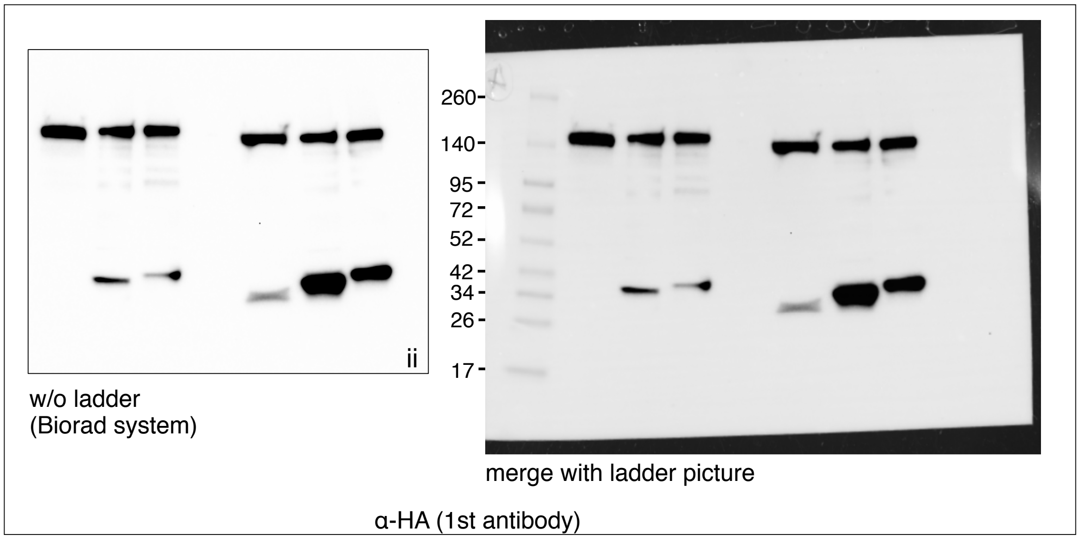

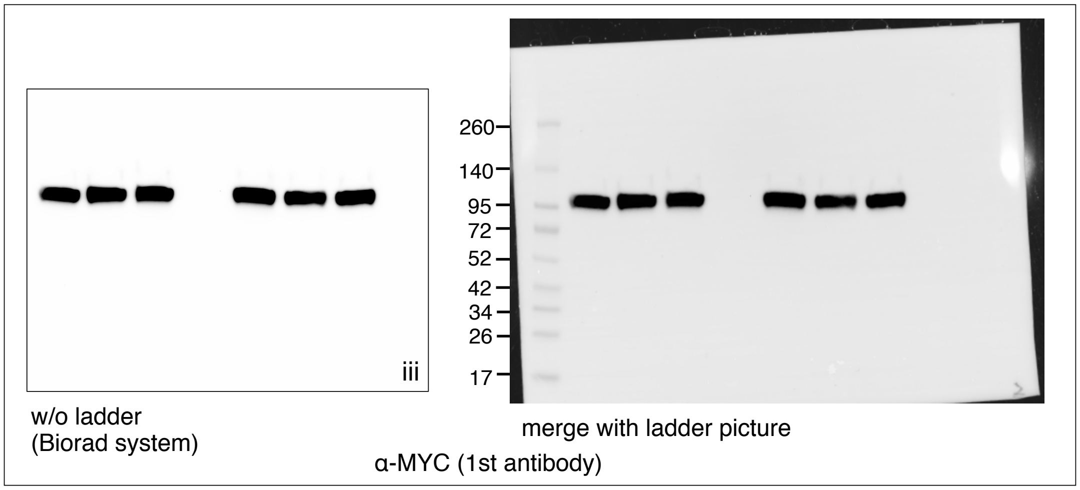

Supplementary Fig. 5c

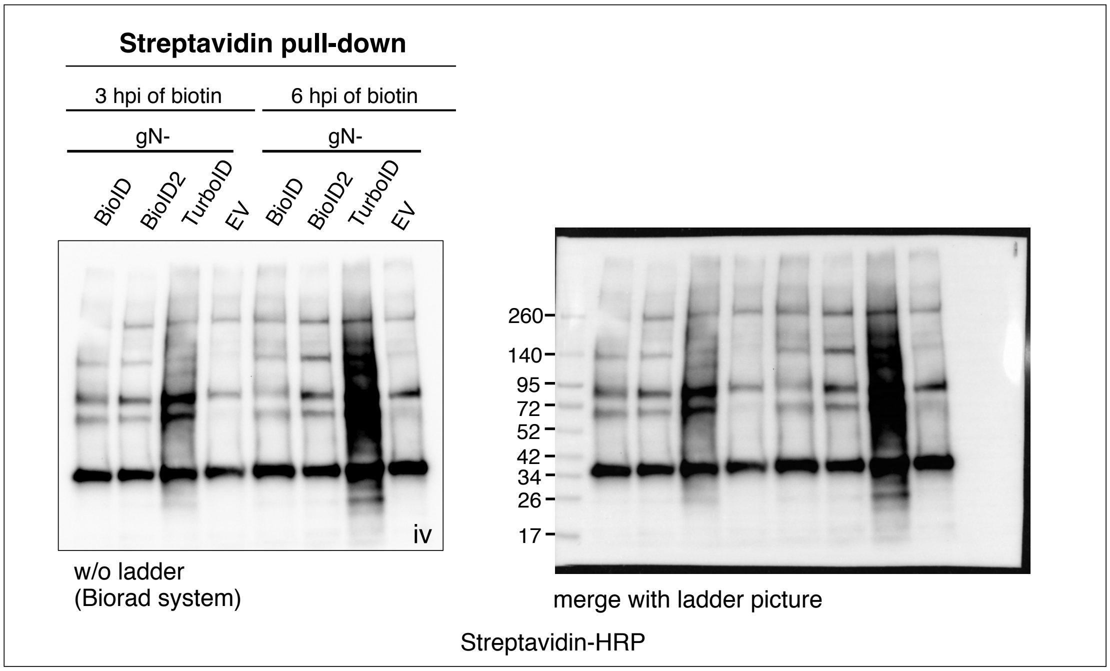

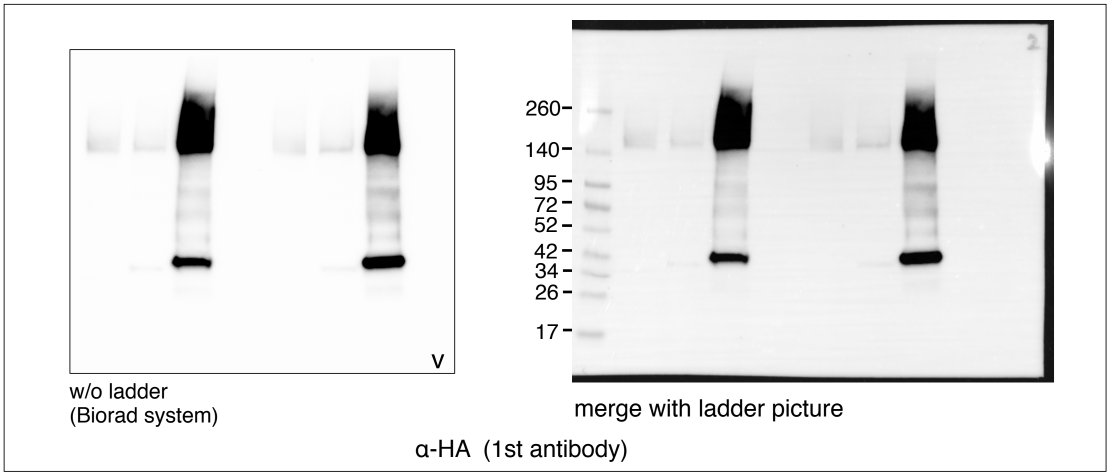

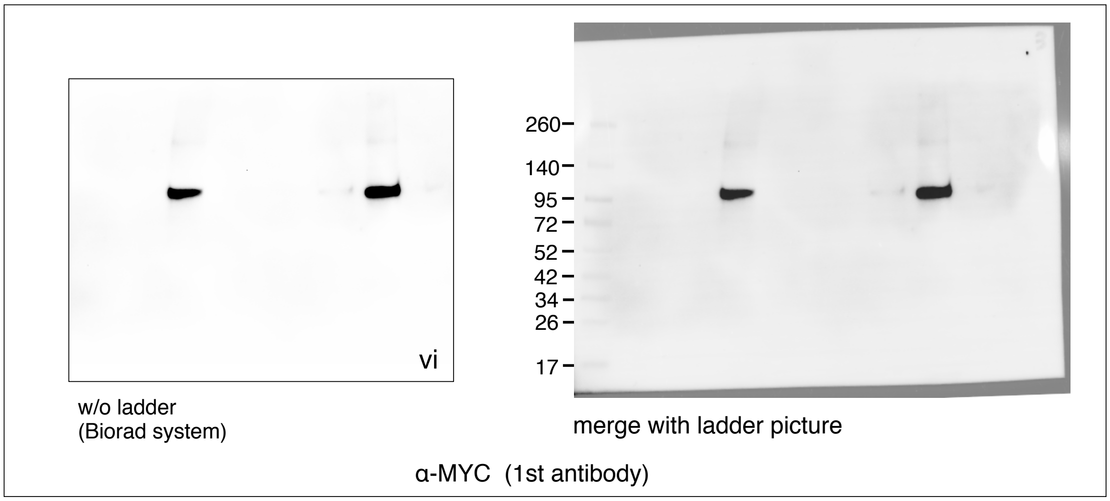

Supplementary Fig. 5d

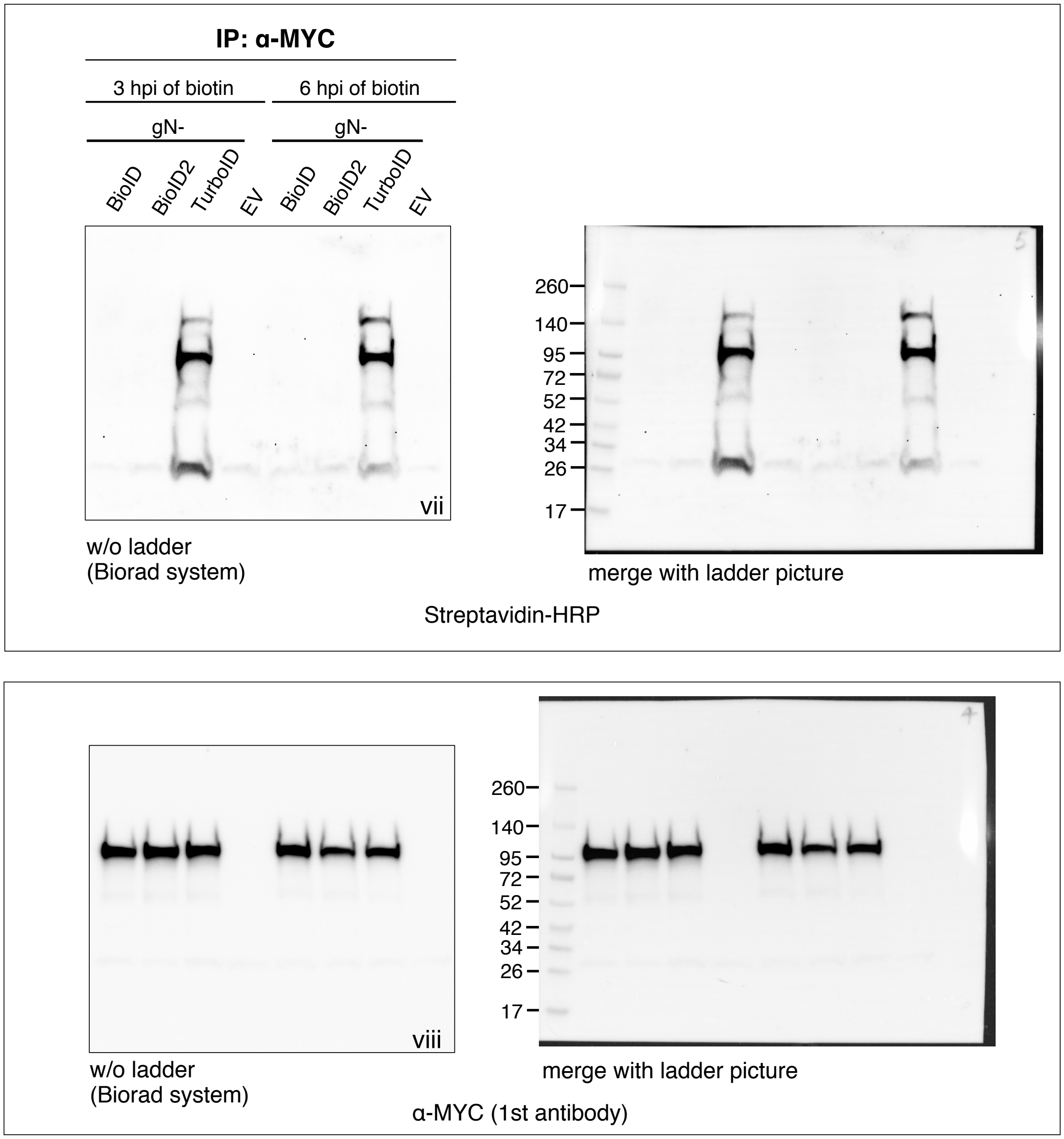

Supplementary Fig. 6b

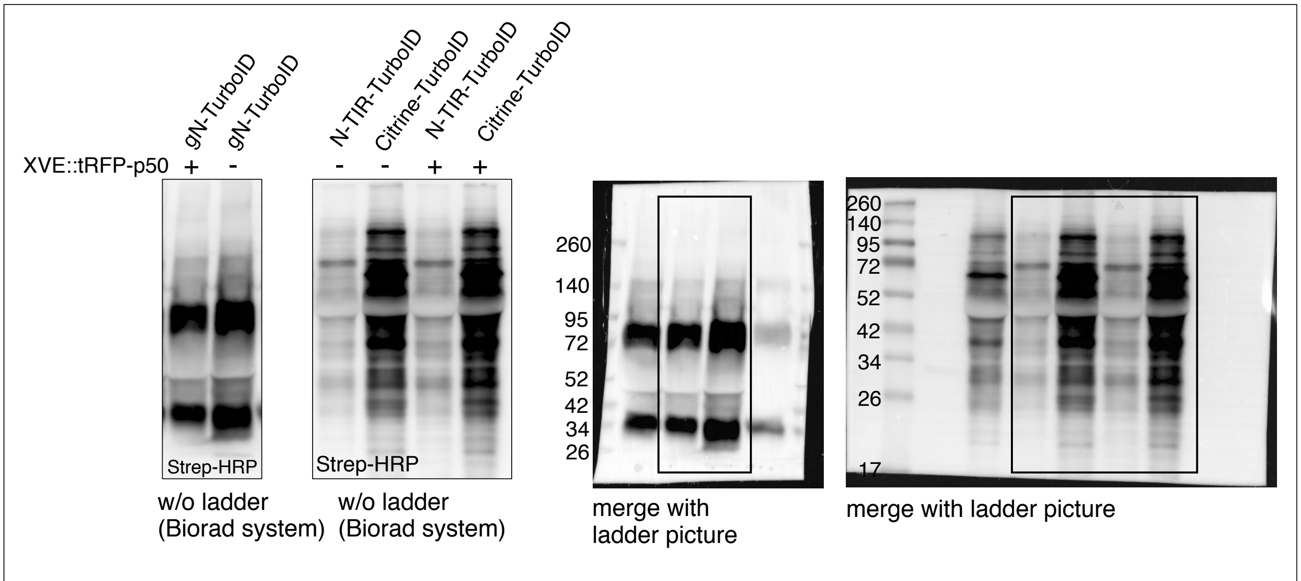

Streptavidin-HRP

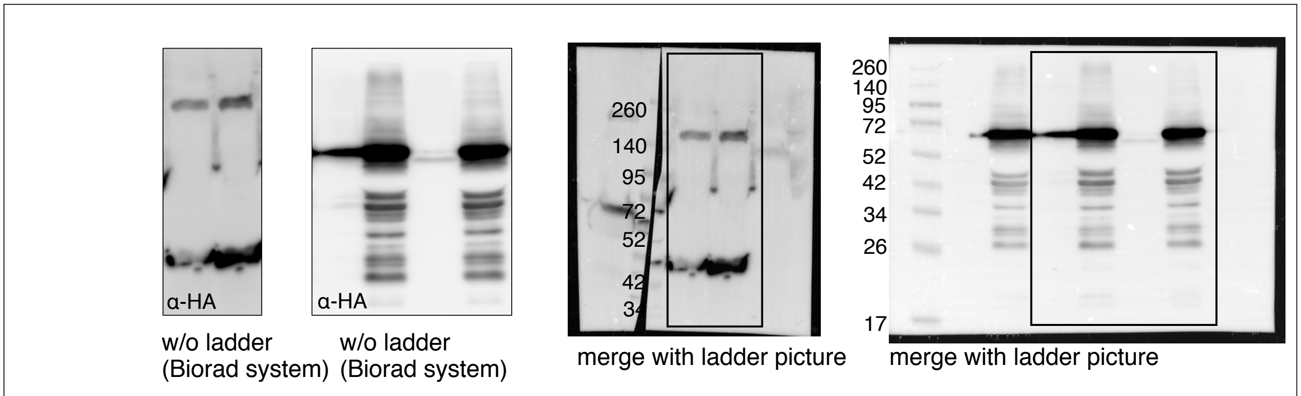

α-HA (1st antibody)

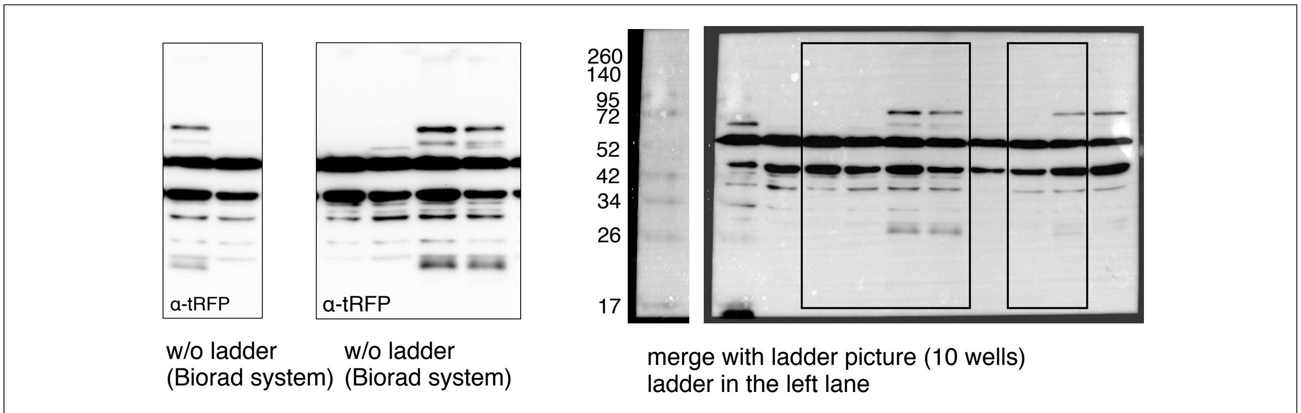

α-tRFP (1st antibody)

Supplementary Fig. 7

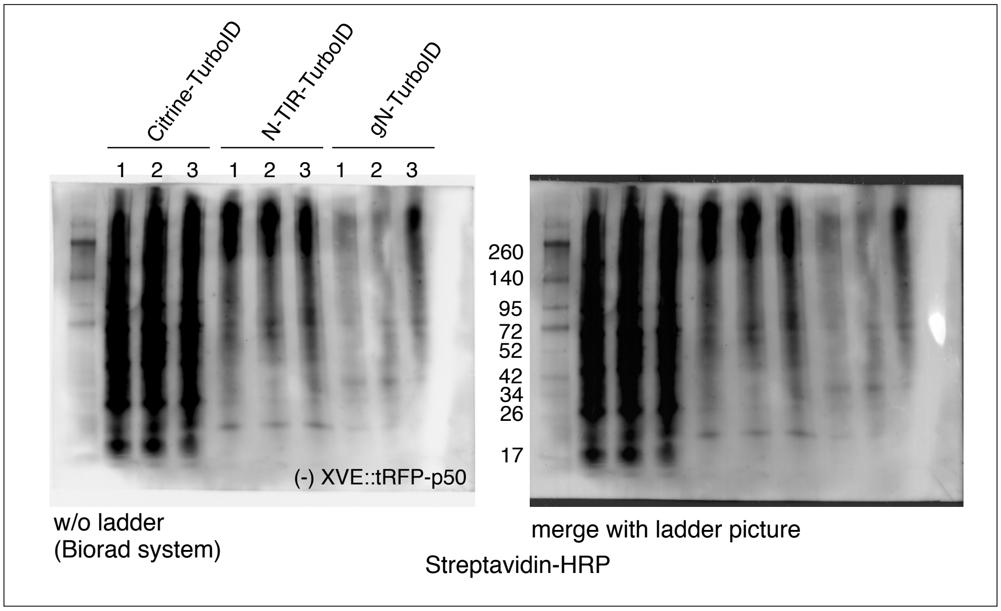

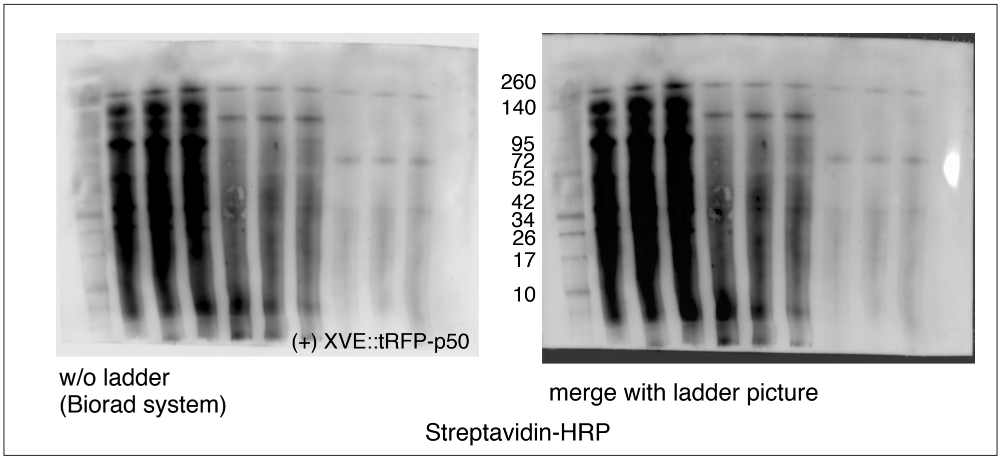

Supplementary Fig. 9b

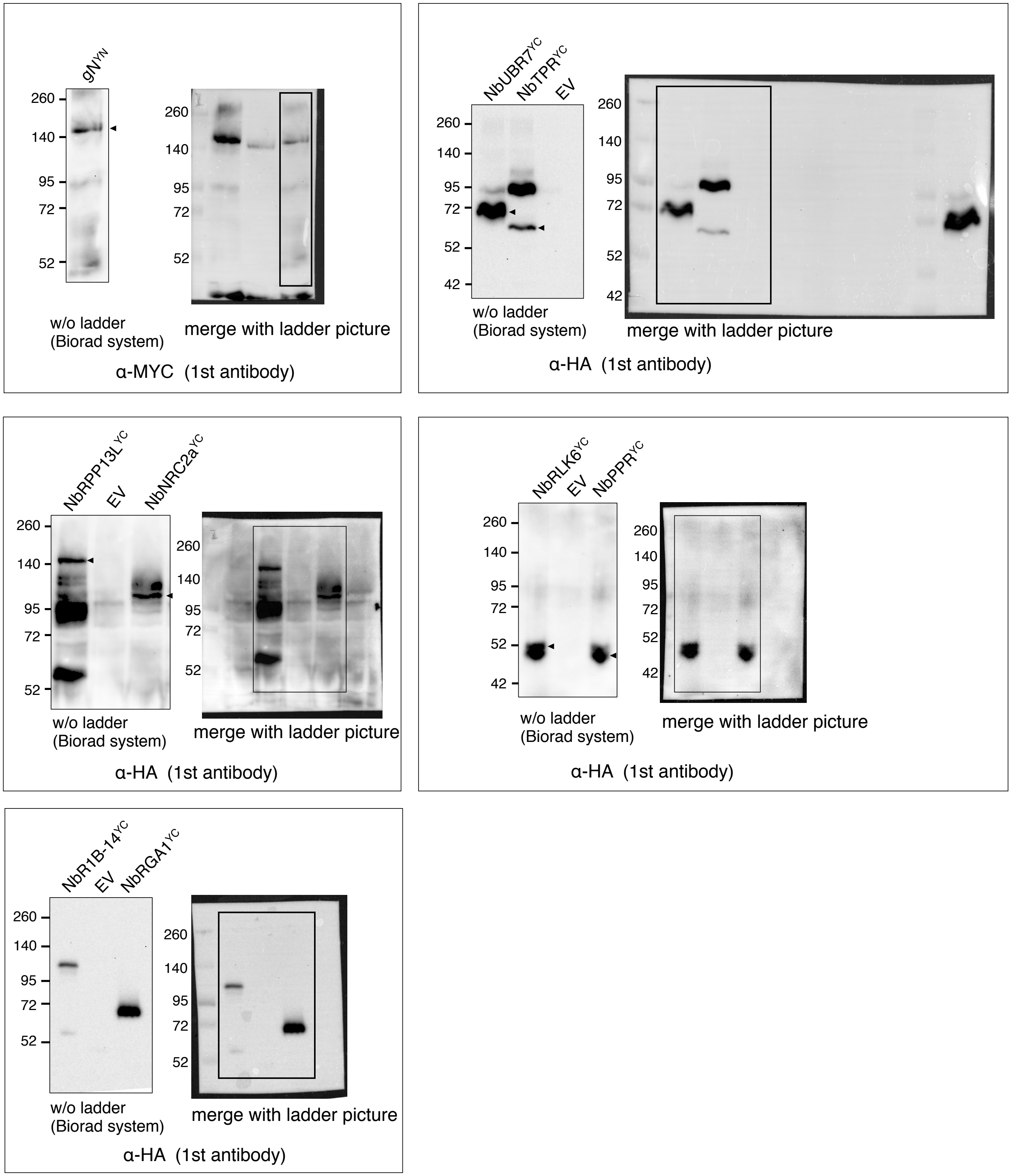

Supplementary Fig. 13

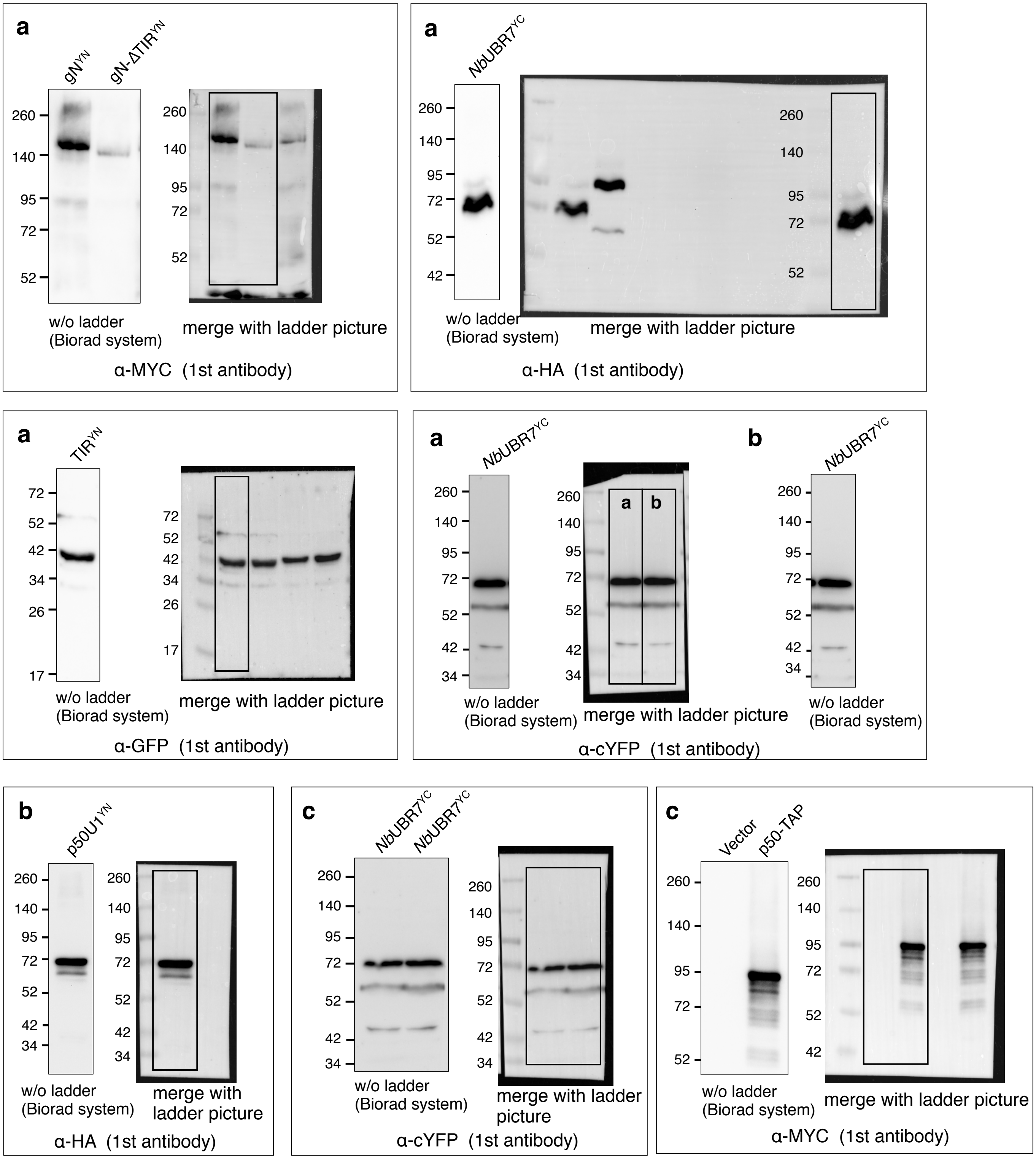

Supplement: Supplementary file 6 — Source Data [file 41467_2019_11202_MOESM6_ESM.pdf]
